# A data-driven semi-parametric model of SARS-CoV-2 transmission in the United States

**DOI:** 10.1371/journal.pcbi.1011610

**Published:** 2023-11-08

**Authors:** John M. Drake, Andreas Handel, Éric Marty, Eamon B. O’Dea, Tierney O’Sullivan, Giovanni Righi, Andrew T. Tredennick

**Affiliations:** 1 Odum School of Ecology, University of Georgia, Athens, Georgia, United States of America; 2 Center for the Ecology of Infectious Diseases, University of Georgia, Athens, Georgia, United States of America; 3 College of Public Health, University of Georgia, Athens, Georgia, United States of America; 4 Western EcoSystems Technology, Inc., Laramie, Wyoming, United States of America; Georgia State University, UNITED STATES

## Abstract

To support decision-making and policy for managing epidemics of emerging pathogens, we present a model for inference and scenario analysis of SARS-CoV-2 transmission in the USA. The stochastic SEIR-type model includes compartments for latent, asymptomatic, detected and undetected symptomatic individuals, and hospitalized cases, and features realistic interval distributions for presymptomatic and symptomatic periods, time varying rates of case detection, diagnosis, and mortality. The model accounts for the effects on transmission of human mobility using anonymized mobility data collected from cellular devices, and of difficult to quantify environmental and behavioral factors using a latent process. The baseline transmission rate is the product of a human mobility metric obtained from data and this fitted latent process. We fit the model to incident case and death reports for each state in the USA and Washington D.C., using likelihood Maximization by Iterated particle Filtering (MIF). Observations (daily case and death reports) are modeled as arising from a negative binomial reporting process. We estimate time-varying transmission rate, parameters of a sigmoidal time-varying fraction of hospitalized cases that result in death, extra-demographic process noise, two dispersion parameters of the observation process, and the initial sizes of the latent, asymptomatic, and symptomatic classes. In a retrospective analysis covering March–December 2020, we show how mobility and transmission strength became decoupled across two distinct phases of the pandemic. The decoupling demonstrates the need for flexible, semi-parametric approaches for modeling infectious disease dynamics in real-time.

## Author summary

Due to the time-varying nature of numerous drivers of disease transmission, flexible, semi-parametric models of disease transmission are necessary to faithfully represent the complex, hidden dynamic processes that drive epidemics of emerging pathogens like SARS-CoV-2. Adequate models are essential to guide policy decisions. We present a data-driven semi-parametric model of SARS-CoV-2 transmission that embeds a latent process within a mechanistic compartmental model. The latent process sub-model captures temporal variation in precautionary behaviors that cannot be easily measured. We show that temporal variation in transmission strength is best explained by mobility early in the pandemic and by this latent process later in the pandemic. The model is flexible and incorporates known biological parameters and disease transmission processes.

## Introduction

The COVID-19 pandemic in the United States has challenged the capabilities of conventional infectious disease transmission models [1, 2]. Yet, models are critical for guiding policy decisions, updating situational awareness, and retrospectively evaluating the key drivers of transmission and the effectiveness of interventions [3, 4]. The complex interactions of a highly transmissible disease, seasonal forcing, evolving public health messaging and guidance, the rise of new genetic variants, and the long duration of the COVID-19 pandemic have made it difficult to effectively model how the mechanistic drivers of the force of infection changed over time [5]. Lack of adequate data about key behavioral factors, such as the adoption of individual protective behaviors like mask-wearing, adds to the difficulty.

For example, the initial phase of the pandemic was marked by stay-at-home orders across the nation [6]. Human mobility dropped drastically from pre-pandemic levels, slowing the spread of SARS-CoV-2 [7]. Publicly available cell phone mobility data allowed modelers to explicitly estimate the impact of human movement—a proxy for person-to-person contact—in mechanistic models of disease transmission [5, 8, 9]. However, when states began to re-open in April 2020, mobility data became a weaker correlate of transmission strength [4, 10]. Precautionary measures such as masking and social-distancing in public spaces weakened the relationship between mobility and transmission risk [4]. But no consistent and reliable data exist to capture the dynamics of all precautionary behaviors over time. Carefully designed and constrained models can infer these latent dynamics, allowing for more accurate situational awareness and forecasting [4].

**Fig 1:**
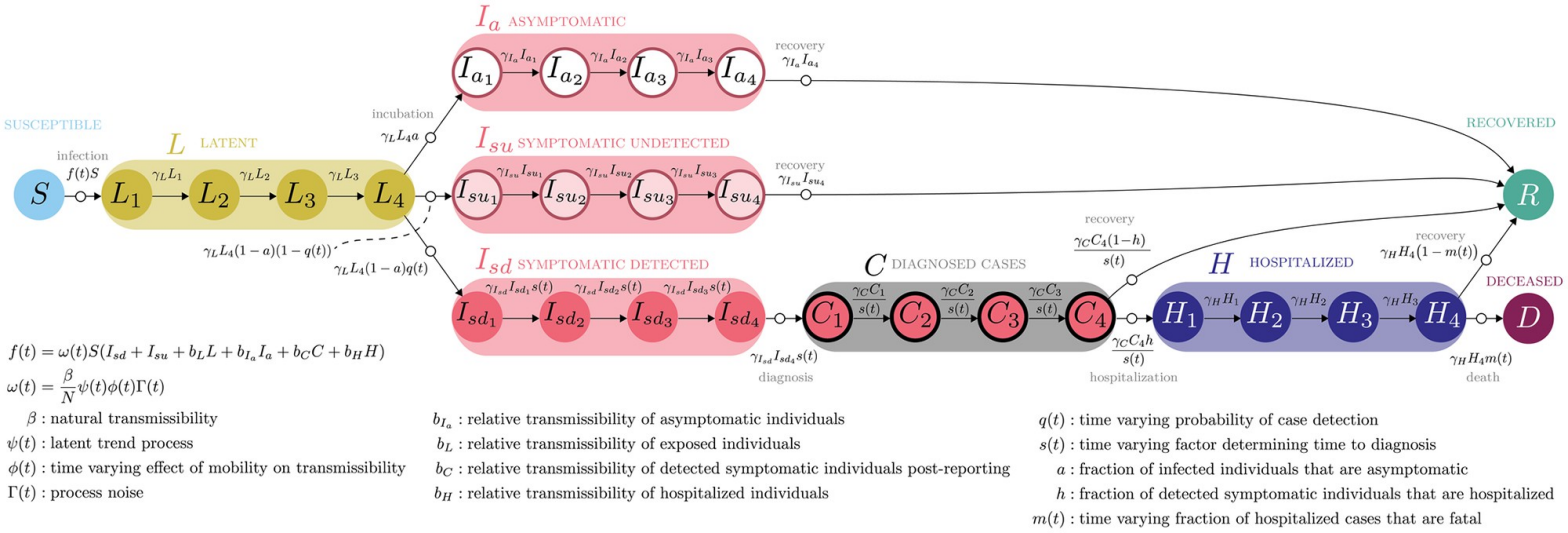
Compartmental model of SARS-CoV-2 transmission.

Here, we present a data-driven semi-parametric compartmental model of SARS-CoV-2 transmission. The stochastic model includes compartments for latent (exposed but pre-symptomatic), asymptomatic, detected and undetected symptomatic individuals, and hospitalized cases (Fig 1). The model also includes time-varying rates of detection probability, diagnosis time, and mortality, all of which add realism to the model, improve identifiability, and enable stronger inference on remaining dynamics. In our model, transmission rate is allowed to differ among asymptomatic, pre-symptomatic, and symptomatic individuals. Force of infection is a function of the number of individuals in each compartment, the relative mobility of individuals, and a latent process. The latent process is a time-dependent spline function (see Materials and methods) representing all of the processes influencing the force of infection that cannot be measured. The model was developed in real-time during 2020 and fitted jointly to daily case and deaths data from each U.S. state and the District of Columbia (hereafter collectively referred to as “US states”) from the date of first case notification in each state to December 31, 2020. For this publication, case and death reports were retrieved from the Johns Hopkins University Coronavirus Resource Center [11] on July 18, 2022, meaning any revisions to the data due to time-lags or changes in inclusion criteria for 2020 were applied. Human mobility data were obtained from Unacast [12]. We restricted our analysis to March 1, 2020 to December 31, 2020, a logical endpoint for retrospective analysis because the model does not include vaccination, and vaccines began to be administered in late December 2020.

To demonstrate the usefulness of our semi-parametric model, we perform a retrospective analysis showing that transmission strength (effective reproduction number) and mobility became decoupled over time. By “decoupled”, we mean two time-varying variables are correlated in one period and not correlated in another. This conclusion could not be arrived at with traditional parametric models. Correlation analysis shows that relative mobility among US states was highly correlated over time, regardless of pandemic phase. To the contrary, however, transmission strength became uncorrelated among states as different states had different levels of adherence to precautionary behaviors [13]. We then show how the relative mobility trend and the latent process trade off in importance over time, so that transmission alternately synchronized with or decoupled from mobility. These dynamics can only be captured with a flexible, data-driven modeling approach when key underlying processes cannot be completely measured.

## Results

**Fig 2:**
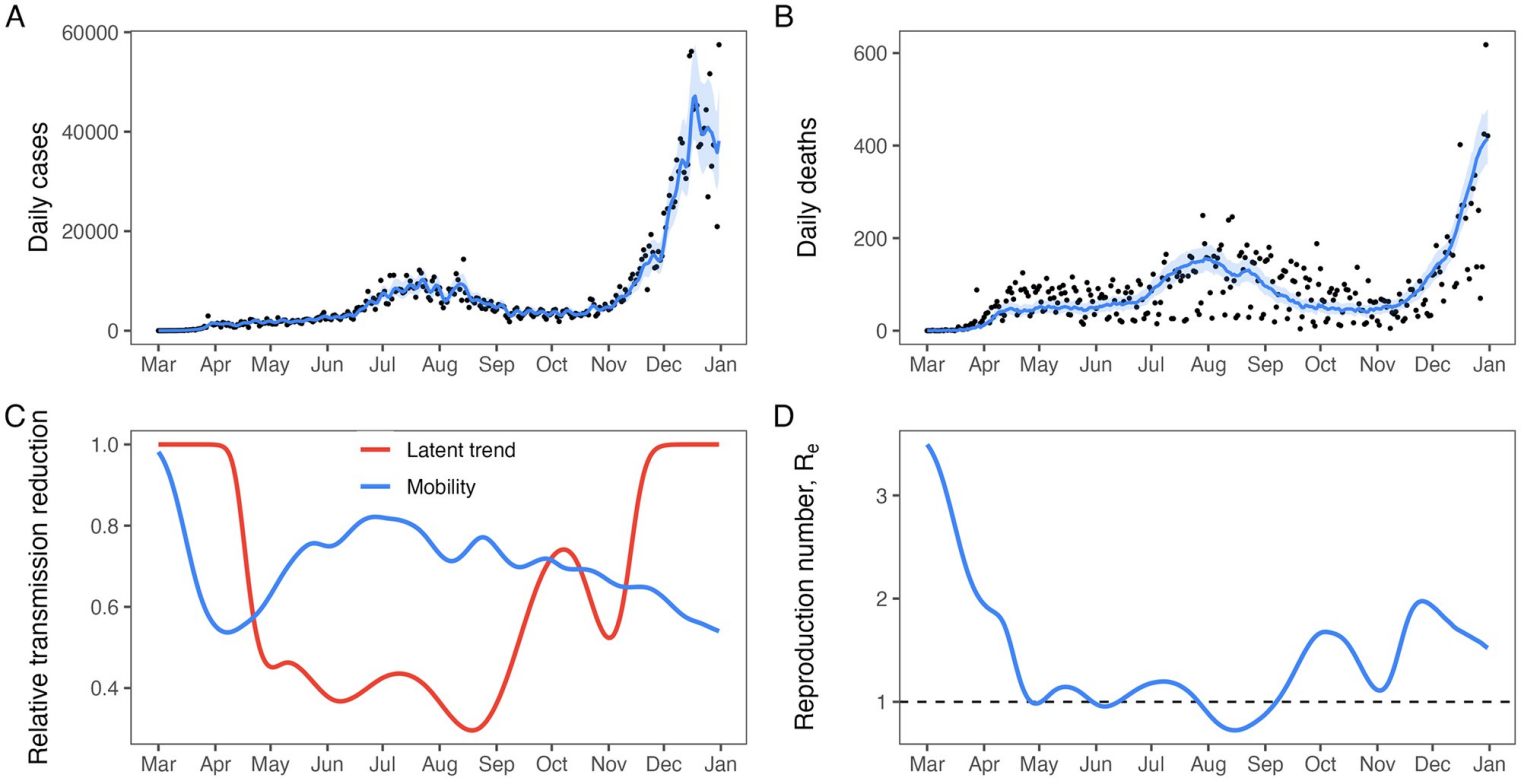
Model fits and estimated quantities for California. A comparison of estimated and observed (A) COVID-19 daily cases and (B) COVID-19 daily deaths show the course of the COVID-19 pandemic in California from March–December 2020. In A and B, lines show the median estimated state variable, ribbons show the 95% prediction interval, and points are noisy, recorded observations. In panel (C), the estimated relative mobility trend (blue line) and the estimated latent trend (red line) are shown to vary considerably over time resulting in dramatic fluctuations in the force of infection, giving rise to the multiple waves infection shown in (A) and (B). These fluctuations in the force of infection are illustrated by (D) the estimated effective reproduction number, which crossed the critical boundary at *R*_*e*_ = 1 on numerous occasions during 2020.

The model estimated daily cases and daily deaths that closely match the observed data although allowing for reporting errors resulting from aggregation, reporting delays, weekend effects, and other anomalies (Fig 2A and 2B; model fits for all states are shown in Section C in S1 Appendix). Model performance varied by state and by response variable. The model for 45 states (out of 51 total) had mean absolute scaled errors for new cases less than or equal to one (Fig B in S1 Appendix), meaning that the semi-parametric model performed better than a non-mechanistic model comprising a random walk model with weekly periodicity. However, only 30 states had mean absolute scaled errors for new deaths less than or equal to one (Fig B in S1 Appendix), meaning either the process for modeling the transition from hospitalized cases to deaths or the observation model could be improved. The smoothed trajectories of daily deaths capture the general trends of the data well (Fig 2B and Section C in S1 Appendix.), which suggests the observation model is the limiting factor in our model. This can be seen in Fig 2B, where the fitted trajectory passes through the cloud of data points but fails to capture cyclical reporting patterns. Cyclical reporting patterns are identified, for example, by large numbers of daily deaths being followed by reports of near 0 daily deaths in the middle of the time series (July—September, Fig 2B).

**Fig 3:**
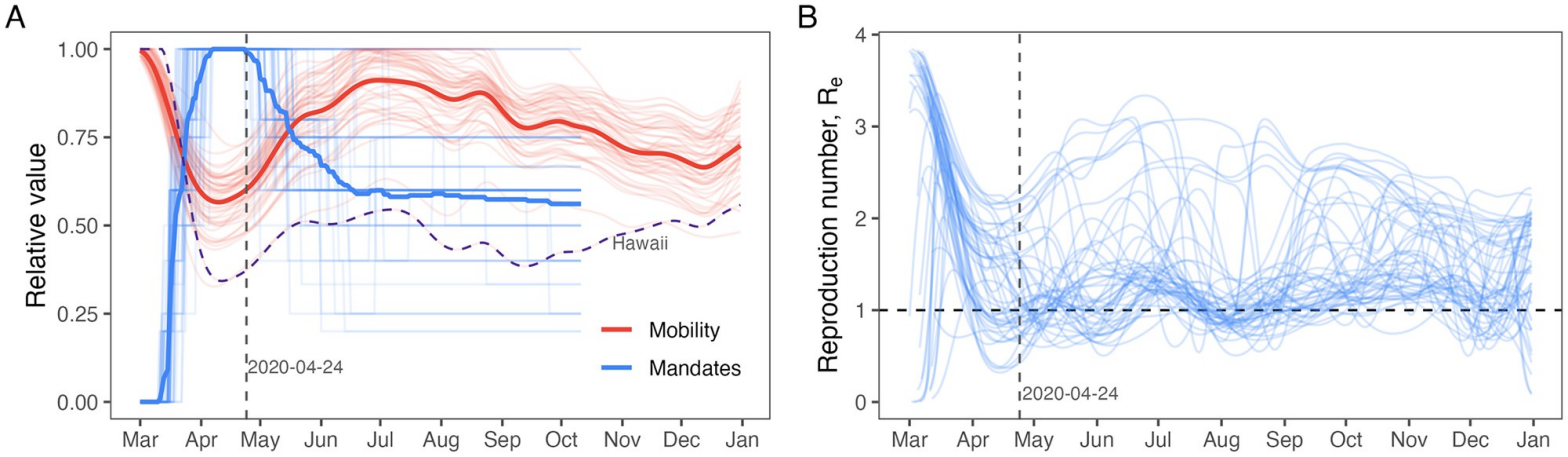
Drivers of force of infection over time. (A) The time series of the relative number of public health mandates (blue lines) and relative mobility (red lines) for each state shows how public health messaging and individual behavior diverge after April 24, 2020 (shown as a vertical dashed line). Relative number of health mandates was calculated as the total number of health mandates issued in each 50 states at each time, divided by the maximum observed over all times in each state. Each light line is a single state. Heavy lines are the average relative mobility and relative number of mandates across all states. The dashed purple line shows the mobility over time for Hawaii, which was the lowest among all states. Before April 24, 2020, public health mandates increased while mobility decreased in all states. After April 24, 2020, public health mandates began to expire and mobility increased, but states began to diverge in their responses, as seen by the larger spread in the light lines after April 24. In panel (B), the estimated effective reproduction number, $R_{e}\left(t\right)$, for each state over time reflects the changes in public health mandates and mobility. Before April 24, $R_{e}\left(t\right)$ declined to below the critical value of 1 (dashed horizontal line) for almost all states. After April 24, $R_{e}=1$ is much more dynamic and variable across states, reflecting the differences in mobility and public health messaging. Analysis of these patterns suggest two distinct phases of the pandemic: Phase 1 before April 24 and Phase 2 after April 24. Critically, even as mobility increased in Phase 2 to near pre-pandemic levels, $R_{e}\left(t\right)$ typically remained near 1.

We also estimated a latent process that, when combined with mobility, uniquely defines transmission strength (Fig 2C). We used the next-generation matrix approach [14] to numerically calculate the time-varying effective reproduction number (Materials and methods), which is a function of mobility, the estimated latent process, and state variables (Figs 2D and 3B).

Analysis of these patterns identified two distinct phases of the early pandemic associated with the time series of relative mobility, the cumulative number of state-issued public health mandates nationwide, and the date at which states began “re-opening” (per CDC definitions: https://www.cdc.gov/museum/timeline/covid19.html). On April 24, 2020, states began re-opening and allowing public health mandates to expire, which unsurprisingly coincided with increasing average mobility (Fig 3A). We use this date to notionally partition two phases of the pandemic from March 1, 2020 to April 24, 2020 (Phase 1) and from April 25, 2020 to December 31, 2020 (Phase 2). In Phase 1, we understand transmission to be almost entirely driven by human-to-human contact. The sharp reduction in human mobility compared with the pre-pandemic baseline resulted in dramatically lowering the effective reproduction number, $R_{e}\left(t\right)$ [4, 8, 15]. In Phase 2, precautionary behaviors like mask wearing and social distancing in public played a much greater role in determining $R_{e}\left(t\right)$ than mobility [4].

**Fig 4:**
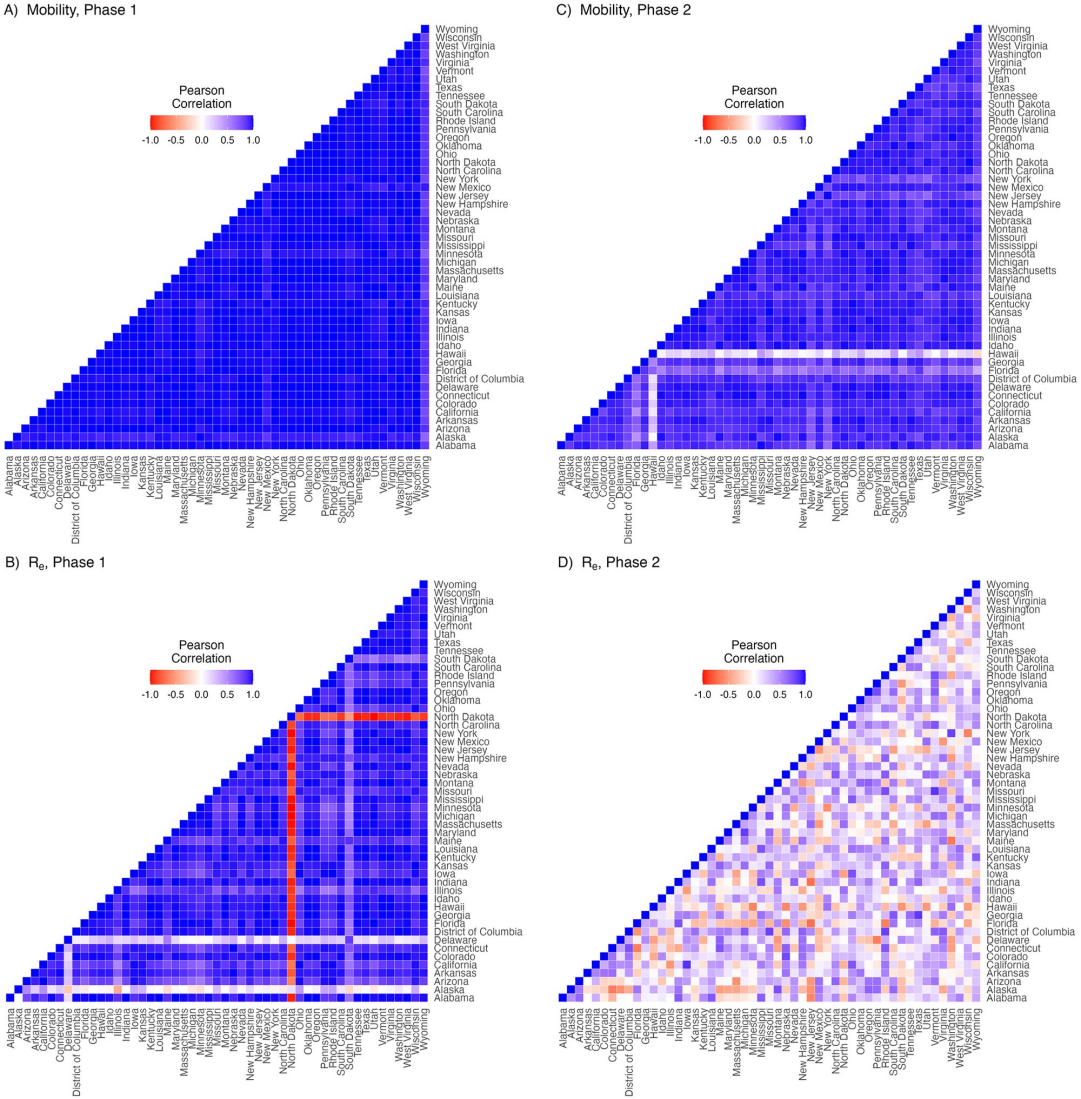
Between-state correlations in mobility and force of infection. Pairwise cross correlations among time series of the (A) relative mobility covariate for each pair of states and (B) among time series of $R_{e}\left(t\right)$ for each pair of states in Phase 1 of the pandemic (March 1, 2020 to April 24, 2020) show that mobility and force of infection were highly correlated across states in Phase 1 of the pandemic. This indicates a tight coupling between mobility and force of infection in the Phase 1. Panels C and D visualize the same pairwise cross correlations during Phase 2 of the pandemic (April 24, 2020 to December 31, 2020), which shows that mobility remains correlated among states while force of infection is uncorrelated. This suggests that mobility is not the main driver of disease dynamics in Phase 2.

Our estimates of $R_{e}\left(t\right)$ display the decoupling of transmission strength from mobility between the two phases (Fig 3B). For instance, pairwise correlations of mobility (mean of the absolute value of Pearson’s *ρ* across all pairs = 0.98, SD = 0.03) and $R_{e}\left(t\right)$ (mean |*ρ*| = 0.86, SD = 0.22) are high among states in the first phase of the pandemic (Fig 4A and 4B). In the second phase of the pandemic, mobility remained highly correlated among states (mean |*ρ*| = 0.88, SD = 0.16; Fig 4C) while $R_{e}\left(t\right)$ became uncorrelated among states (mean |*ρ*| = 0.32, SD = 0.24; Fig 4D).

**Fig 5:**
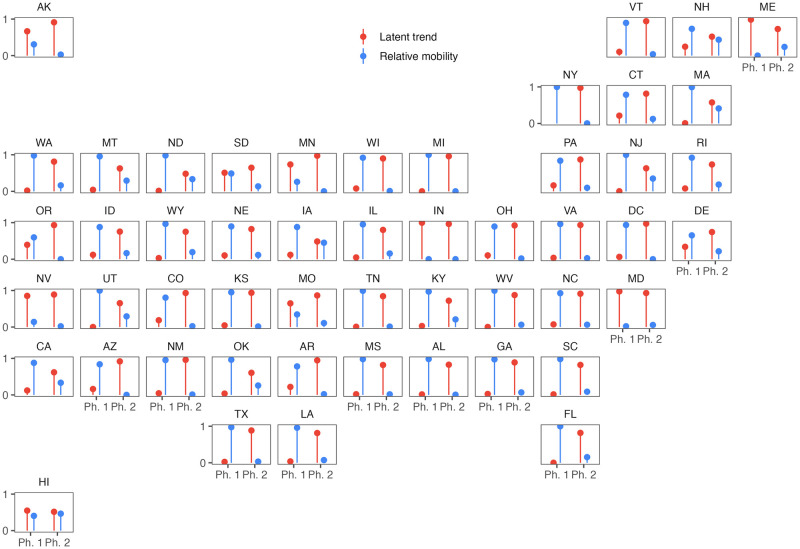
Drivers of temporal variance in force of infection. Comparisons of the amount of temporal variance in $R_{e}\left(t\right)$ that can be explained by mobility (blue bars) and the latent process (red bars) in the two phases of the pandemic shows that, for most states, the temporal variance of $R_{e}\left(t\right)$ switches from being explained by mobility in Phase 1 to being explained by the latent process in Phase 2. The temporal variation in $R_{e}\left(t\right)$ is mostly explained by the latent trend in both phases for several states (e.g., Nevada). Individual panels for each states are arranged by approximate geographic coordinates for each state.

Variance partitioning showed that mobility explained most of the temporal variation of $R_{e}\left(t\right)$ in the first phase of the pandemic and that the latent trend explained most of the variation in $R_{e}\left(t\right)$ in the second phase of the pandemic for nearly all states (Fig 5). The latent process was an important model component for some states in Phase 1 of the pandemic (e.g., Nevada (NV), Fig 5). There were no states for which mobility remained more important than the latent process in determining the temporal variation of $R_{e}\left(t\right)$ in Phase 2 of the pandemic (Fig 5). This is especially noteworthy since relative mobility in Hawaii was the lowest among all states throughout 2020 (Fig 3A). Taken together, these lines of evidence show that $R_{e}\left(t\right)$ decoupled (i.e., not temporally related) from mobility over the course of the pandemic, a feature of the pandemic that we attribute to “societal learning” as the population adopted increasingly nuanced and localized approaches to reducing transmission [16].

## Discussion

The COVID-19 pandemic spurred innovation in epidemiological modeling because models and forecasts of how the pandemic might unfold were in high demand. However, models that cannot reproduce the complex outcomes of nonlinear dynamical systems are not useful for situational awareness, forecasting, or scenario analysis under any but the shortest time horizons [9]. This does not mean that a good model must include mechanistic descriptions of all relevant processes. But, models do need to be able to reproduce observed outcomes (i.e., case, hospitalization, and death reports). We presented a flexible modeling approach that is rooted in the mechanisms of disease transmission and included a latent trend to represent unmeasurable factors that influence transmission. In so doing, we show the decoupling of SARS-CoV-2 transmission from social distancing as other precautionary behaviors became more prevalent.

Our semi-parametric model mostly performed better than a benchmark random walk model for cases. This benchmark random walk model, which is strictly statistical and lacks mechanistic interpretation, was included in another study that compared twenty-eight COVID-19 forecasting models [17]. The benchmark random walk model was found to have intermediate forecasting skill in comparison with the twenty-seven other models [17], making it a good baseline for comparison with our semi-parametric model. The semi-parametric model fits for six states had mean absolute scaled errors (MASE) greater than one (Fig B in S1 Appendix), indicating worse fit than the benchmark random walk model with weekly periodicity. These six states tended to have case reports that oscillated between 0 and 100s in the later weeks of 2020 (Section C in S1 Appendix), likely due to lags in reporting. Because our semi-parametric model did not include an observation process with reporting lags or periodicity, the model performed worse for those states where reporting oscillations were dramatic. The same issue (report lags and periodicity in reporting) resulted in death fits from our model being worse than the benchmark random walk model for 21 states. We suggest that model performance might be improved by including a more complex observation model to account for cyclical variation in reporting [5].

The better fit of the benchmark random walk model in some situations does not invalidate the utility of our model. The benchmark model, a random walk with weekly periodicity, does not include any epidemiology. Therefore, while the random walk model may generate slightly better one-step-ahead predictions in some situations, it does not yield any scientific insight. Our semi-parametric model did perform better than the random walk model for many states and the filtered trajectories of new cases and deaths matched the observations well, even when MASE ≥ 1 (Fig B in S1 Appendix).

The results of variance partitioning suggest that a purely mechanistic model would have performed just as well as our semi-parametric model in the early stages of the pandemic, when human mobility was the main driver of transmission strength. However, our model performed well across all stages of the pandemic because mobility was allowed to drive transmission early in the pandemic and then trade-off with the latent process in latter stages. The flexibility of the time-varying latent process is critical because the intensity of precautionary behaviors varied over time. Some analyses have sought to address this issue using additional data streams such as the presence of mask mandates over space and time [18]. In contrast, our flexible time-evolving process avoids the need for additional data streams.

Other modelers have used temporal random effects to estimate time-varying transmission strength [4, 19]. For example, Fox et al. [4] modeled time-varying transmission as a function of two time-specific random walks and a normally distributed temporal random effect. Modeling discrete change-points in transmission strength is another approach [20]. But the first- and second-order discontinuities of change-points hardly reflect the way that behaviors change over time and their influence on the ebb and flow of transmission. Our spline approach achieves the desired outcome—letting the relationship between mobility and transmission change over time—while also directly quantifying the contribution of mobility and other forces to moderating transmission. Indeed, our approach is more flexible because the number of basis functions used to estimate the B-spline can also be fitted or, if needed, trimmed to the minimum number possible for computationally efficient model fitting.

We estimated maximum $R_{e}\left(t\right)$ values in range of 1.52–3.84 across all 50 states. Highest $R_{e}\left(t\right)$ occurred at the start of the epidemic (Fig 3B and Section D in S1 Appendix). These estimates are similar to an $R_{0}$ estimated from meta-analysis, 3.38 ±1.40 [21]. Our estimates are lower than those reported for some individual cities and municipalities, probably because our state-level model is too coarse to capture super-spreading events that happen at local scales [22]. For example, Fox et al. [4] reported a 7-day average $R_{e}\left(t\right)$ of 5.8 at the start of the epidemic in Austin, Texas and Hasan et al. [22] estimated $R_{e}\left(t\right)$ greater than 5 in the Jakarta–Depok region of Indonesia.

Our analysis has implications for modeling the next pandemic. First, as reported by others, adherence to precautionary behaviors is not static and is not always measurable [4, 23]. This means that modeling approaches need built-in flexibility to estimate latent trends, such as the time-dependent spline function we used. Second, incorporating latent trends makes situational awareness easier (i.e., answering the question “What is the current trend in the force of infection?”), but it makes forecasting harder. Estimating trends in transmission is easier with a data-driven model because the data have a large influence on model dynamics and summary quantities such as $R_{e}\left(t\right)$. Forecasting is more difficult, however, because the latent trend must be specified for future times at which (by definition) no data exist. This means that latent, smooth trends that were specifically designed to fluctuate over time must be, in practice, extrapolated in some fashion. New methods are arising to handle forecasting with latent processes when the underlying function is periodic [24]. But the transmission dynamics of an emerging pathogen are not periodic, meaning subjective choices must be made to specify the latent process at future times if forecasting is the objective. One possibility is to use the estimated number of cases to predict hospitalizations and/or deaths, but only up to future time points within the typical duration between case notification, hospitalization, and death. Such forecasts would be relatively short-term by definition.

Flexible, semi-parametric models of disease transmission are an effective way to faithfully represent complex, hidden, and time-evolving transmission in outbreaks of emerging pathogens [4, 5, 25]. Human behavior and disease transmission are inherently linked and each generates feedback to the other. We believe it will never be possible to perfectly and fully represent human behavior. Nonetheless, carefully crafted models, such as the model we presented and others [4, 5, 19], can infer the impact of human behavior when fitted to data. The semi-parametric model we have presented is capable of using more input data (e.g., survey data on mask-wearing) or less (e.g., no mobility data), making it flexible enough to perform statistical inference regardless of how much information about covariates is available. This feature could be of substantial value to models of the ongoing COVID-19 pandemic, where information is accumulating over time, as well as future pandemics, where initial modeling efforts will also be hobbled by the lack of information.

## Materials and methods

### Data

We fit the model to daily case and death reports for each state in the USA collated by the Center for Systems Science and Engineering at Johns Hopkins University [11]. We did not smooth the reported data but we did redefine all negative case and death reports as NA-values. Time-series of case and death reports for each state used in this analysis are shown in Section C of S1 Appendix. Data were downloaded on 2022-07-18, meaning any revisions to data from 2020 were applied. For example, data sets were often back-filled after reporting lags and some states changed the inclusion criteria for a death to be considered primarily a cause of COVID-19. We do not specifically account for these revisions or changes in data over time in this analysis.

Data on human mobility were downloaded from Unacast (https://www.unacast.com/). The data were pre-aggregated by Unacast, and were the difference in daily distance traveled relative to baseline distance (Janurary 2020), averaged over individuals in each state. We converted the data to a relative difference in mobility by adding 1 to each value with a positive sign (greater than baseline) and subtracting the absolute value from 1 for all values with a negative sign (less than baseline). We then fit a smoothing spline through the time series to generate smooth covariate for modeling. The fitted splines for each state are shown in the Section D of S1 Appendix.

### Model

The model comprises susceptible, pre-symptomatic, asymptomatic, symptomatic, diagnosed, hospitalized, deceased, and recovered persons (Table 1). The infectious compartments (pre-symptomatic, asymptomatic, symptomatic, diagnosed, and hospitalized persons) differ in their transmissibility and are thus defined by their impacts on population-level transmission rather than clinical symptoms. To reflect realistic distributions of movement through compartments, the *L*, *I*_*a*_, *I*_*sd*_, *I*_*su*_, *C*, and *H* compartments are internally split into four stages using the linear chain trick [26] (Fig A in S1 Appendix). Transitions between compartments were modeled using the Euler multinomial approximation given the size of the “donating” compartment and the specified or fitted rate of transition, as implemented in the R [27] package **pomp** [28] version 2.7.1.0.

**Table 1:** State variable symbols and definitions in the model.

Symbol	Definition
***S***	Uninfected and susceptible individuals. Susceptible individuals can become infected by individuals in the *L*, *I*_*a*_, *I*_*su*_, *I*_*sd*_, *C*, and *H* stages.
***L***	Individuals with latent infections who do not yet show symptoms. Those individuals can be infectious. At the end of the *L* stage, a fraction moves into the *I*_*a*_ stage, another fraction moves into the *I*_*su*_ stage, and the remainder into the *I*_*sd*_ stage.
***I*_*a*_**	Individuals who are infected and asymptomatic.
***I*_*su*_**	Individuals who are infected and symptomatic, but are undetected.
***I*_*sd*_**	Individuals who are infected and symptomatic, and are detected. Individuals in this compartment will be diagnosed and move to *C*.
***C***	Individuals who have been diagnosed as cases. Those individuals are likely isolated and have reduced infectiousness. A fraction of individuals in the *C* stage will naturally recover, without the need for hospitalization. The remainder move into the *H* stage.
***H***	Individuals who have been hospitalized. Those individuals are likely isolated and have reduced infectiousness. A fraction of individuals in the *H* stage will recover, the remainder will die.
***R***	Recovered/removed individuals. Those individuals have recovered and are immune.
***D***	Individuals who died from the infection.

Ignoring the internal splits of multi-stage compartments, the stochastic model is defined as the set of difference equations

$$\begin{array}{cc} S(t+1)-S(t) & =-{\text{n}}_{1}\\ L(t+1)-L(t) & ={\text{n}}_{1}-{\text{n}}_{2}-{\text{n}}_{3}-{\text{n}}_{4}\\ I_{a}\left(t+1\right)-I_{a}\left(t\right) & ={\text{n}}_{2}-{\text{n}}_{5}\\ I_{su}\left(t+1\right)-I_{su}\left(t\right) & ={\text{n}}_{3}-{\text{n}}_{6}\\ I_{sd}\left(t+1\right)-I_{sd}\left(t\right) & ={\text{n}}_{4}-{\text{n}}_{7}\\ C(t+1)-C(t) & ={\text{n}}_{7}-{\text{n}}_{8}-{\text{n}}_{9}\\ H(t+1)-H(t) & ={\text{n}}_{8}-{\text{n}}_{10}-{\text{n}}_{11}\\ R(t+1)-R(t) & ={\text{n}}_{5}+{\text{n}}_{6}+{\text{n}}_{9}+{\text{n}}_{10}\\ D(t+1)-D(t) & ={\text{n}}_{11} \end{array}$$

with

$$\begin{array}{cc} \left(\begin{array}{c} {\text{n}}_{S}\\ {\text{n}}_{1} \end{array}\right) & ∼\text{EM}(S\left(t\right),(\begin{array}{c} e^{-f(t)\Delta t}\\ 1-e^{-f(t)\Delta t} \end{array}))\\ \left(\begin{array}{c} {\text{n}}_{L}\\ {\text{n}}_{2}\\ {\text{n}}_{3}\\ {\text{n}}_{4} \end{array}\right) & ∼\text{EM}(L\left(t\right),(\begin{array}{c} e^{-\gamma_{L}\Delta t}\\ a(1-e^{-\gamma_{L}\Delta t})\\ \left(1-q\left(t\right)\right)\left(1-a\right)(1-e^{-\gamma_{L}\Delta t})\\ q\left(t\right)\left(1-a\right)(1-e^{-\gamma_{L}\Delta t}) \end{array}))\\ \left(\begin{array}{c} {\text{n}}_{I_{a}}\\ {\text{n}}_{5} \end{array}\right) & ∼\text{EM}(I_{a}\left(t\right),(\begin{array}{c} e^{-\gamma_{I_{a}}\Delta t}\\ 1-e^{-\gamma_{I_{a}}\Delta t} \end{array}))\\ \left(\begin{array}{c} {\text{n}}_{I_{su}}\\ {\text{n}}_{6} \end{array}\right) & ∼\text{EM}(I_{su}\left(t\right),(\begin{array}{c} e^{-\gamma_{I_{su}}\Delta t}\\ 1-e^{-\gamma_{I_{su}}\Delta t} \end{array}))\\ \left(\begin{array}{c} {\text{n}}_{I_{sd}}\\ {\text{n}}_{7} \end{array}\right) & ∼\text{EM}(I_{sd}\left(t\right),(\begin{array}{c} e^{-\gamma_{I_{sd}}\Delta t}\\ 1-e^{-\gamma_{I_{sd}}\Delta t} \end{array}))\\ \left(\begin{array}{c} {\text{n}}_{C}\\ {\text{n}}_{8}\\ {\text{n}}_{9} \end{array}\right) & ∼\text{EM}(C\left(t\right),(\begin{array}{c} e^{-\gamma_{C}\Delta t}\\ s{\left(t\right)}^{-1}h(1-e^{-\gamma_{C}\Delta t})\\ s{\left(t\right)}^{-1}\left(1-h\right)(1-e^{-\gamma_{C}\Delta t}) \end{array}))\\ \left(\begin{array}{c} {\text{n}}_{H}\\ {\text{n}}_{10}\\ {\text{n}}_{11} \end{array}\right) & ∼\text{EM}(H\left(t\right),(\begin{array}{c} e^{-\gamma_{H}\Delta t}\\ \left(1-m\left(t\right)\right)(1-e^{-\gamma_{H}\Delta t})\\ m\left(t\right)(1-e^{-\gamma_{H}\Delta t}) \end{array})), \end{array}$$

where n_*X*_ is the number of individuals that remain in each compartment *X*; *f*(*t*) is the force of infection at time *t*; *γ*_*L*_, $\gamma_{I_{a}}$, $\gamma_{I_{su}}$, $\gamma_{I_{sd}}$, *γ*_*C*_, and *γ*_*H*_ are the transition rates out of each respective compartment; *q*(*t*) is the time varying probability of case detection; *s*(*t*) is a time varying factor determining time to diagnosis; *a* is the fraction of infected individuals that are symptomatic; *h* is the fraction of detected symptomatic individuals that are hospitalized; and *m*(*t*) is the time varying fraction of hospitalized cases that are fatal. EM stand for the Euler Multinomial process described by He et al. [29] with the rates (e.g., *e*^−*f*(*t*)Δ*t*^) described and stochastic noise descriptions excluded. See He et al. [29] for more details.

The force of infection *f*(*t*) was modeled as:

$$\begin{array}{cc} f(t)=\; & \omega (t)\times S(t)\;\times \\ & (I_{sd}\left(t\right)+I_{su}\left(t\right)+b_{L}L+b_{I_{a}}I_{a}\left(t\right)+b_{C}C\left(t\right)+b_{h}H\left(t\right)), \end{array}$$

where *b*_*L*_, $b_{I_{a}}$, *b*_*C*_, and *b*_*H*_ are relative transmissibility factors for each respective compartment. The time-dependent transmission rate is a function of mobility, a latent trend, and process noise as:

$$\begin{array}{c} \omega \left(t\right)=\frac{\beta }{N}\psi \left(t\right)\phi \left(t\right)\Gamma \left(t\right), \end{array}$$

where *N* is the constant total population size of the state, *ψ*(*t*) is relative human mobility at time *t*, and *ϕ*(*t*) is the latent trend of relative transmission strength, specified as the spline function

$$\begin{array}{c} \text{logit}\;(\phi (t))=\sum_{i=1}^{K}g_{i}\xi_{i_{t}}, \end{array}$$

where *K* is the number of basis functions, **g** is the vector of spline coefficients, and ***ξ*** is the matrix of basis functions. The number of basis functions, *K*, was defined as the number of days in each time-series divided by 21 (one function every 21 days [3 weeks]). Process noise Γ(*t*) was modeled as gamma-distributed white noise (temporally uncorrelated noise) with mean 1 and variance *σ*^2^ [30].

Time-varying detection probability (*q*(*t*)) was modeled as a Hill function starting at 10% (*q*_min_) on day 0 and increasing to a maximum possible of 40% (*q*_max_), reaching that half way point of 25% on the 30th day (*q*_half_) since the first case notification:

$$\begin{array}{c} q\left(t\right)=q_{\text{min}}+\frac{q_{\text{max}}\times q_{\text{r}}^{t}}{q_{\text{r}}^{q_{\text{half}}}+q_{\text{r}}^{t}}, \end{array}$$

where *q*_r_ is the rate of increase of the Hill function, which is set to 1.1, and *t* is the day since first case notification (see Section A of S1 Appendix).

Time-varying decrease in time-to-diagnosis (1/*s*(*t*) days) was similarly defined as:

$$\begin{array}{c} s\left(t\right)=1+\frac{s_{\text{max}}\times s_{\text{r}}^{t}}{s_{\text{r}}^{s_{\text{half}}}+s_{\text{r}}^{t}}, \end{array}$$

where *s*_max_ is the maximum decrease in days to diagnosis, *s*_half_ is the day on which the decrease in days to diagnosis is half-way between 0 and *s*_max_ (set to day 30), and *s*_r_ is rate of increase of Hill function, set to 1.1 (see Section A of S1 Appendix). Note that *s*(*t*) is multiplied by $\gamma_{I_{sd}}$ to increase the diagnosis rate, and that *γ*_*C*_ is divided by *s*(*t*) to slow the rate from *C* to *H*, increasing the amount of time in the *C* compartment to account for reduced time in the *I*_*sd*_ compartment.

Time-varying mortality fraction (*m*(*t*)) was also specified as a Hill function:

$$\begin{array}{c} m\left(t\right)=m_{\text{base}}+\frac{m_{\text{min}}\times m_{\text{r}}^{t}}{m_{\text{r}}^{m_{\text{half}}}+m_{\text{r}}^{t}}, \end{array}$$

where *m*_base_ is the baseline fraction of hospitalizations that result in death, *m*_min_ is the minimum fraction of hospitalizations that result in death, *m*_half_ is the days since first case notification at which *m*(*t*) is halfway between *m*_base_ and *m*_min_, and *m*_r_ is the Hill function rate of change, fixed to 1.

We calculated the effective reproduction number ($R_{e}\left(t\right)$) using the next-generation matrix approach [14]. Assuming that all of the time dependent functions, *S*, *ω*, *s*, *q*, are changing slowly over the course of an individual’s infection, the expected number of new cases each case generates, $R_{e}\left(t\right)$, may be approximated as:

$$\begin{array}{cc} R_{e}\left(t\right)\approx \; & S\left(t\right)\cdot \omega \left(t\right)\cdot 4\cdot [b_{L}/\gamma_{L}+\left(1-a\right)\;\times \\ & (q\left(t\right)\left(b_{I_{sd}}/\left(s\gamma_{I_{sd}}\right)+b_{C}s/\gamma_{C}+hb_{H}/\gamma_{H}\right)\;+\\ & \left(1-q\left(t\right)\right)b_{I_{su}}/\gamma_{I_{su}})+ab_{I_{a}}/\gamma_{I_{a}}]. \end{array}$$

This equation may be arrived at by assuming that all time-varying parameters are fixed to the value at time *t* over the course of an infection and multiplying the expected residence time in each infectious stage by the transmission rate, the number of susceptible individuals, and the probability of a newly infected individual experiencing it.

We assumed that new, daily case reports arise from a negative binomial distribution whose central tendency is captured by the flow of individuals from *I*_*sd*4_ to *C*_1_. We denote this quantity by *C*_new_, which accumulates over the course of one day in the simulation model and resets to zero at the end of each day (the model is simulated at a time step of 1/20 days). Similarly, we assumed that new, daily death reports arise from a negative binomial distribution whose central tendency is captured by the flow of individuals from *C*_4_ to *D*. We denote this quantity by *D*_new_, which accumulates over the course of one day in the simulation model and resets to zero at the end of each day. Then, for both new cases and deaths, we modeled the observation process as:

$$\begin{array}{c} \text{cases}\left(t\right)∼\text{NB}(C_{\text{new}}\left(t\right),\theta_{C})\\ \text{deaths}\left(t\right)∼\text{NB}(D_{\text{new}}\left(t\right),\theta_{D}) \end{array}$$

where *θ*_*C*_ and *θ*_*D*_ are the negative binomial (NB) dispersion parameters for cases and deaths, respectively. Note that cases(*t*) and deaths(*t*) are the observed number of cases or deaths reported on day *t*.

### Model fitting

We fit the model using Maximization by Iterated particle Filtering (MIF). Observations (daily case and death reports) were modeled as arising from a negative binomial reporting process (see above). We estimated 24 to 26 parameters for each state: the baseline fraction of hospitalized cases that result in death (*m*_base_), the minimum fraction of hospitalized cases that result in death (*m*_min_), the number of days since first case notification at which *m*(*t*) is half-way between *m*_base_ and *m*_min_ (*m*_half_), a parameter accounting for extra-demographic process noise (*σ*), two negative binomial dispersion parameters (*θ*_*c*_ and *θ*_*d*_), the initial size of the latent and infectious classes (*L*(*t* = 0), *I*_*a*_(*t* = 0), *I*_*su*_(*t* = 0), *I*_*sd*_(*t* = 0)), and 14 to 16 coefficients (*g*_*i*_ for *i* ∈ (1, 2, 3, …, 14(16)) specifying the latent trend spline. The number of B-spline coefficients depended on the length of the time series for each state. The estimated latent trend multiplied by the relative mobility covariate multiplied by $R_{0}$ (fixed to 7 for all states) specifies the time-varying transmission rate. All other parameters were fixed at the values reported in Table 2.

**Table 2:** Fixed model parameters.

Parameter definition	Parameter symbol	Value	Source
Reproduction number	$R_{0}$	7	[31]
Baseline transmission rate of symptomatic individuals	*β*	$R_{0}\times 0.1\times \frac{1}{N}$	Assumption
Fraction of infected individuals that are asymptomatic	*a*	0.18	[32]
Fraction of diagnosed cases that are hospitalized	*h*	0.12	[33]
Relative transmissibility of *L* to *I*_*su*/*sd*_	*b*_*L*_	0.12	[34]
Relative transmissibility of *I*_*a*_ to *I*_*su*/*sd*_	$b_{I_{a}}$	0.5	[34]
Relative transmissibility of *C* to *I*_*su*/*sd*_	*b*_*C*_	0.27	Assumption
Relative transmissibility of *H* to *I*_*su*/*sd*_	*b*_*H*_	4.5 × 10^−5^	[35]
Duration of time in *L* stages	1/*γ*_*L*_	4 days	[34]
Duration of time in *I*_*a*_ stage	$1/\gamma_{I_{a}}$	3.5 days	[34]
Duration of time in *I*_*su*_ stages	$1/\gamma_{I_{su}}$	6 days	[34]
Duration of time in *I*_*sd*_ stage	$1/\gamma_{I_{sd}}$	$0.5\times 1/\gamma_{I_{su}}$	Assumption
Duration of time in *C* stages	1/*γ*_*C*_	$0.5\times 1/\gamma_{I_{su}}$	Assumption
Duration of time in *H* stages	1/*γ*_*H*_	6 days	[36]
Minimum detection probability	*q*_min_	0.1	Assumption
Maximum detection probability	*q*_max_	0.4	Assumption
Day at which detection probability is halfway between *q*_min_ and *q*_max_	*q*_half_	30	Assumption
Rate of increase from *q*_min_ to *q*_max_	*q*_r_	1.1	Assumption
Maximum factor by which diagnosis speed increases	*s*_max_	1.0	[37]
Day at which diagnosis speed-up factor (*s*) is halfway between 0 and *s*_max_	*s*_half_	30	Assumption
Rate of increase from 0 to *s*_max_	*s*_*r*_	1.1	Assumption
Initial size of susceptible pool	*S*(*t* = 1)	*N*_state_	Assumption

Initial size of the susceptible compartment was set as each state’s population size minus the number of individuals in other the other compartments that we fix. It is true that the initial size of the susceptible pool will also decrease based on the number of individuals estimated to be in the latent infections compartment at *t* = 1. However, given the small size of the latent and infectious compartments relative to total population size, and the fact that total population size is a point estimate with error, we assume that our simple approach of setting *S*(*t* = 1) to each state’s population size is valid. For each state, we considered *t* = 1 as the date on which the first case is reported in that state.

We used the IF2 algorithm [38] implemented in the R [27] package **pomp** version 2.7.1.0. [28] to perform MIF. To initialize IF2, we generated 100 parameter sets from a range of parameter values using a Sobol sequence sampling design (Table 3). We then performed two rounds of MIF, each for 100 iterations with 3,500 particles and geometric cooling. For the first round of MIF we set cooling.factor = 1.0. For the second round of MIF, which continues from where the first round stopped, we set cooling.factor = 0.9. The log likelihoods of the parameter sets following MIF were calculated as the log of the mean likelihoods of 20 replicate particle filters with 5,000 particles each. At this stage, we collected all parameter sets within 10 negative log likelihood points of the maximum and sampled a new collection of 100 parameter sets, with sampling weighted in proportion to the negative log likelihood of the parameter set. All parameters in the new set were perturbed as: *p*_new_ ∼ Normal(*p*, |*p* × 0.25|). The perturbed parameter sets were then used to initialize two final rounds of MIF, each run for 50 iterations with 3,500 particles. Cooling factors were 1 in the penultimate MIF round and 0.5 in the final MIF round. At this stage, we assume the parameter set with highest log likelihood is the MLE.

**Table 3:** Estimated parameters and starting ranges for MIF estimation procedure. The expit function refers to back-transforming the parameter from the logit scale, which was used for estimation.

Parameter definition	Parameter symbol	Start range
Baseline fraction of hospitalizations that result in death	*m*_base_	[expit(-6), expit(6)]
Minimum fraction of hospitalizations that result in death	*m*_min_	[expit(-6), expit(6)]
Day at which death fraction is halfway between *m*_base_ and *m*_min_	*m*_half_	[exp(-5), exp(5)]
Extra-demogaphic process noise	*σ*	[exp(-5), exp(5)]
Case reporting dispersion	*θ*_*c*_	[exp(-5), exp(5)]
Death reporting dispersion	*θ*_*d*_	[exp(-5), exp(5)]
Initial size of latent compartment	*L*(*t* = 0)	[exp(0), exp(10)]
Initial size of asymptomatic infectious compartment	*I*_*a*_(*t* = 0)	[exp(0), exp(10)]
Initial size of undetected infectious compartment	*I*_*su*_(*t* = 0)	[exp(0), exp(10)]
Initial size of detected infectious compartment	*I*_*sd*_(*t* = 0)	[exp(0), exp(10)]
B-spline coefficients	*g*_*i*_	[-10, 10]

Following [4], we calculated smoothed posterior estimates of all time-varying states using replicate particle filtering. Specifically, we ran 500 replicate particle filters with 2,500 particles at the MLE, retaining one randomly sampled complete particle trajectory from each particle filter. The 500 trajectories generated a set of 500 smoothed posterior draws of all time-varying state variables and parameters. $R_{e}\left(t\right)$ was calculated at each time *t* for each of the 500 smoothed posteriors using the equation presented above, yielding a smoothed posterior distribution for $R_{e}\left(t\right)$. We used the median of the posterior distribution of $R_{e}\left(t\right)$ for all analyses. The estimated, time-varying latent trend was calculated using the MLEs for the B-spline coefficients.

### Correlation analysis

We calculated pairwise cross correlations of the time series of relative mobility and estimated latent trends (median of *ψ*) among all locations (states) using Pearson’s correlation coefficient. The correlations were estimated for both phases of the pandemic (see main text) using the cor() function in R.

### Variance partitioning

We split the time series of $R_{e}$ (effective reproduction number), *ψ* (latent transmission trend), and *ϕ* (relative human mobility) for each state into two periods: pre-April 24, 2020 and post-April 24, 2020 (see main text). For each period and each state, we partitioned the variance of $R_{e}$ over time among *ψ* and *ϕ*. We used the median of the smoothed posterior distributions for *ψ* and $R_{e}$. We fit two linear regressions to explain $R_{e}$ over time: an intercept only model and a full model with the mobility trend and the latent trend as additive covariates: R_e ∼ mobility + latent. Models were fitted using the lm() function in R. We extracted the sum of squared residuals for each model parameter using the anova() function in R. Proportion of variance explained by each covariate was calculated as the sum of squared errors associated with each covariate divided by the sum of squared errors of the null model.

## Supporting information

S1 Appendix Additional model details and results. S1 Appendix comprises five sections: A) graphical displays of the detection probability (*q*(*t*)) and diagnosis time (1/*s*(*t*)) functions; B) graphical display of mean absolute scaled errors for each state for new case and new death reports; C) time series of incident case and death reports for each state with model-estimated filtered trajectories overlaid; and D) time series of mobility, estimated latent trend, and effective reproduction number for each state.
(PDF)
