# Supplementary material for: A data-driven semi-parametric model of SARS-CoV-2 transmission in the United States
Source: PLoS Comput Biol. 2023 Nov 8;19(11):e1011610. doi: 10.1371/journal.pcbi.1011610 (PMC10659176; doi:10.1371/journal.pcbi.1011610)
Supplement: S1 Appendix — comprises five sections: A) graphical displays of the detection probability (q(t)) and diagnosis time (1/s(t)) functions; B) graphical display of mean absolute scaled errors for each state for new case and new death reports; C) time series of incident case and death reports for each state with model-estimated filtered trajectories overlaid; and D) time series of mobility, estimated latent trend, and effective reproduction number for each state. (PDF) [file pcbi.1011610.s001.pdf]

Supplementary Information: A data-driven semiparametric model of SARS-CoV-2 transmission dynamics in the United States

John M. Drake, Andreas Handel, Éric Marty, Eamon B. O'Dea, Tierney O'Sullivan, Giovanni Righi, and Andrew T. Tredennick

2023-11-02

Contents

A. Detection probability and diagnosis speed-up functions **2**

B. Mean absolute scaled errors (MASE) **3**

C. Time series of incident case and death reports **4**

D. Time series of mobility, estimated latent trend, and $\mathcal{R}_e$ **21**

A. Detection probability and diagnosis speed-up functions

The fraction of exposed individuals that are detected and flow into the I_{sd} compartments (q) starts at a low level of 0.1 and increases sigmoidally towards a maximum of 0.4, reaching the half way point of 0.25 on the 30th day since the first case notification. The time to diagnosis ($1/s$) decreases sigmoidally over time from 3 days on day 0 towards a minimum of 1.5 days, reaching the half way point of 2.25 days on the 30th day since the first case notification.

Both q, s follow a Hill function with Hill coefficient 1.1 and time to half maximum of 30 days:

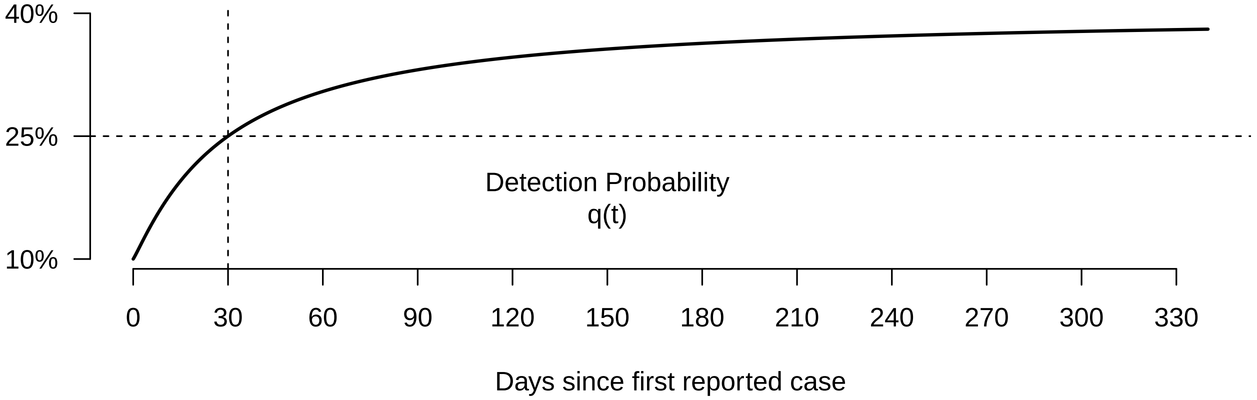

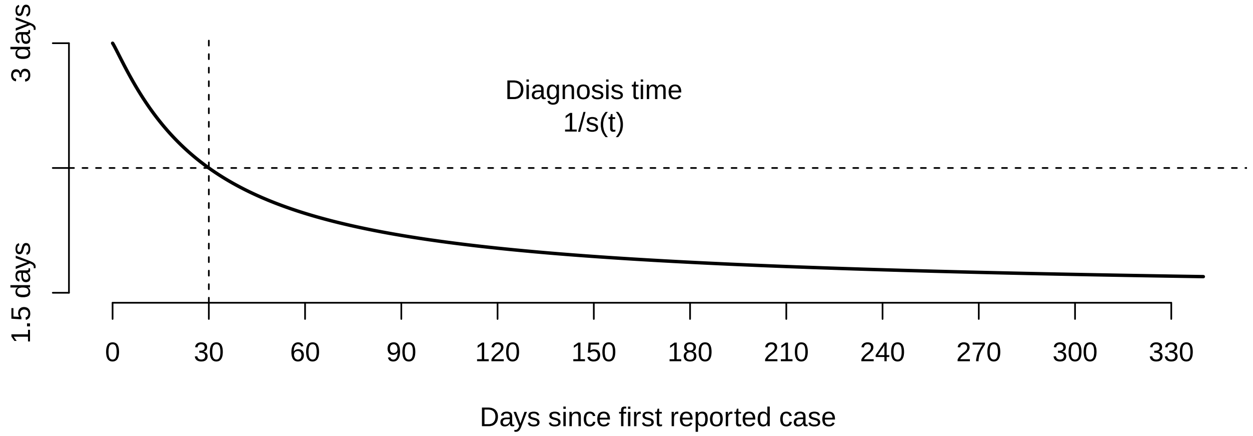

B. Mean absolute scaled errors (MASE)

The MASE is a ratio of mean absolute error (MAE) in the model to MAE of an in-sample naïve model (a random walk forecast from the last observation, adjusted for weekly seasonality). MASE values were calculated for each of the 500 particle filter replicates for each state using the **yardstick** package in R. Most states had a mean MASE less than one for cases, while MASE was greater than one for deaths for most states. Thus, our model does not always outperform the benchmarking model, but it does offer mechanistic insight that the random walk model does not.

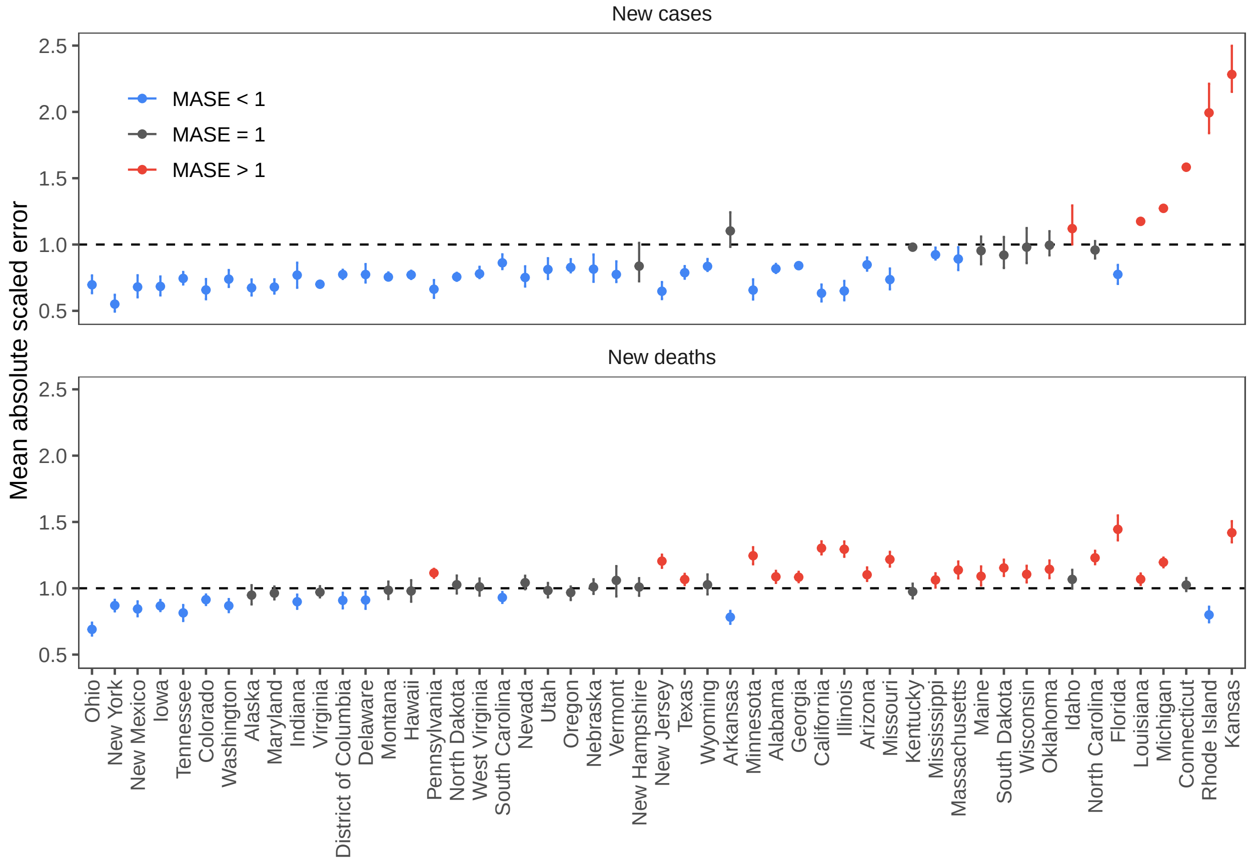

Figure A: Mean and 95% intervals of mean absolute scaled errors (MASE) for each state. MASE was calculated for each of the 500 particle filter replicates for each state, yielding a distribution of MASE values. The point shows the mean of the MASE distribution and the errorbars show the 95% interval (lower 0.025 quantile to upper 0.975 quantile). States are ranked approximately from lowest MASE to highest MASE across both cases and deaths.

C. Time series of incident case and death reports

In the following plots, orange points are the reported daily cases or deaths, white line shows the median of the smoothed filtering distributions, and the grey ribbon bounds the 95% prediction interval.

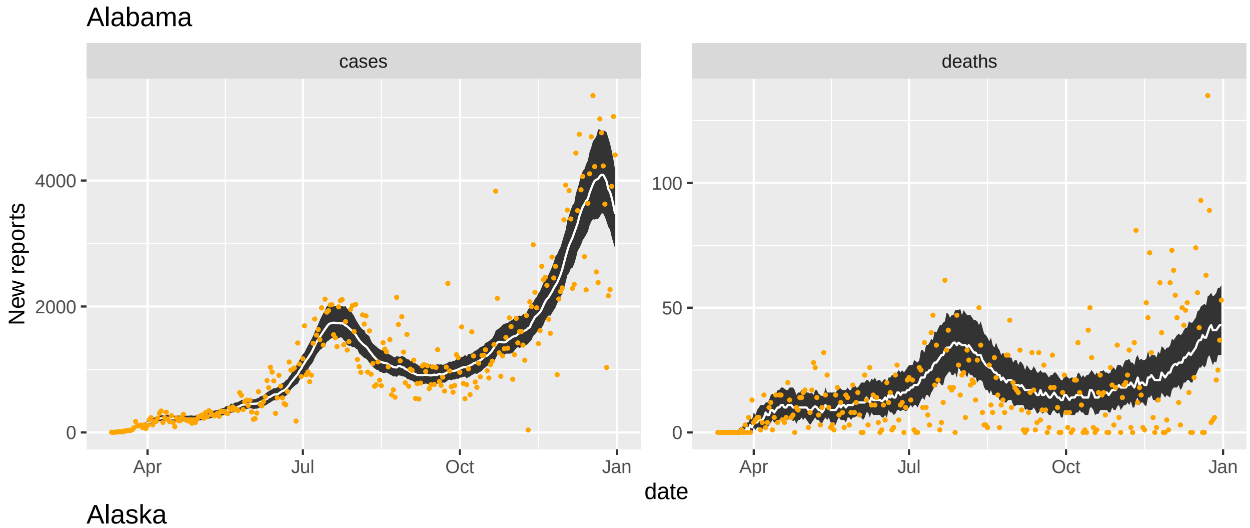

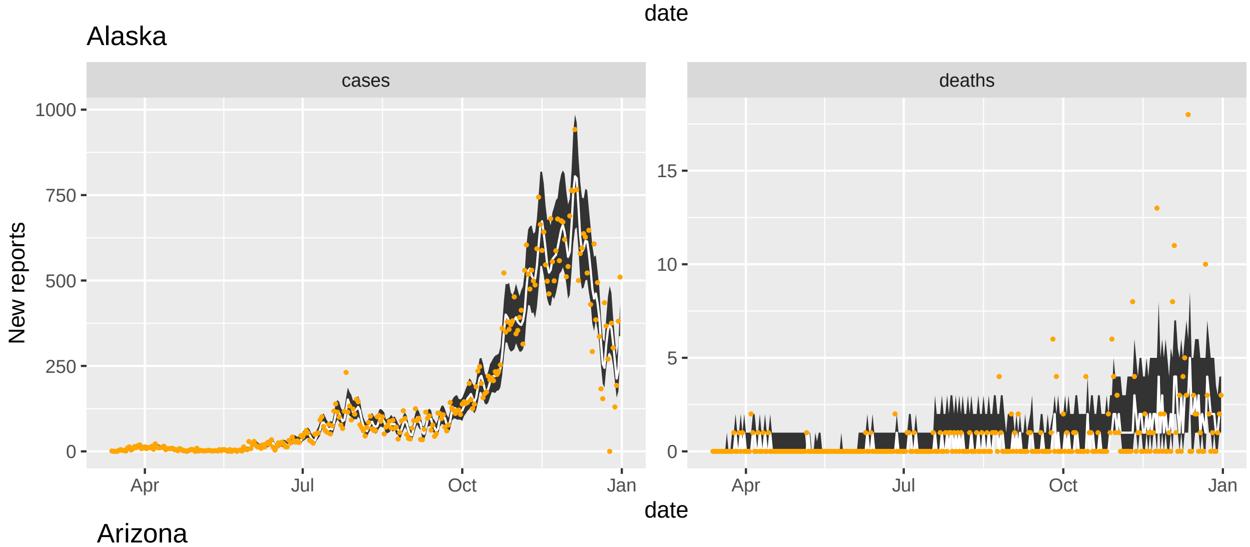

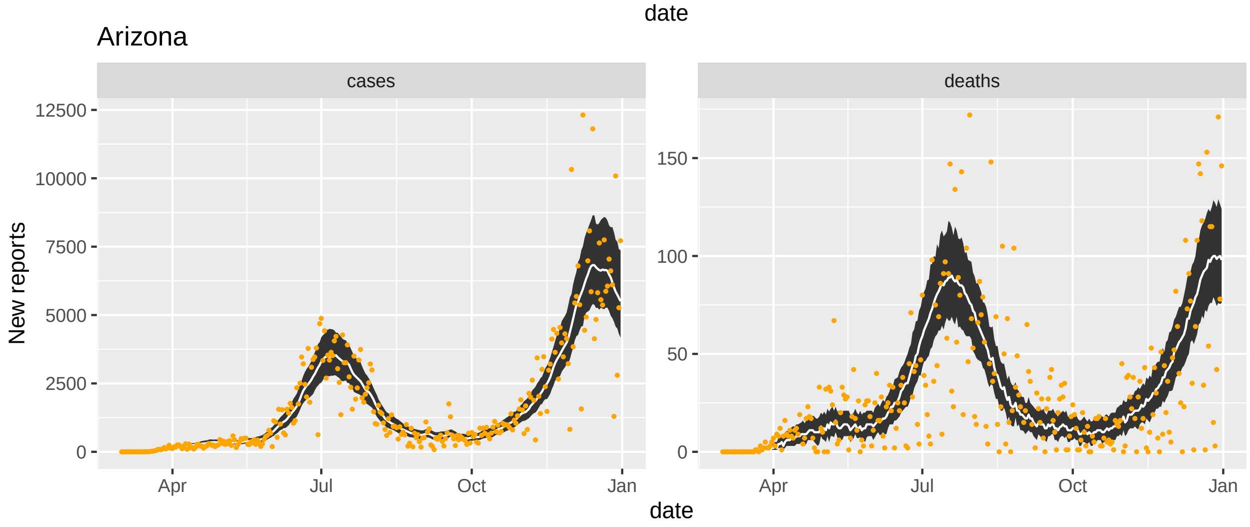

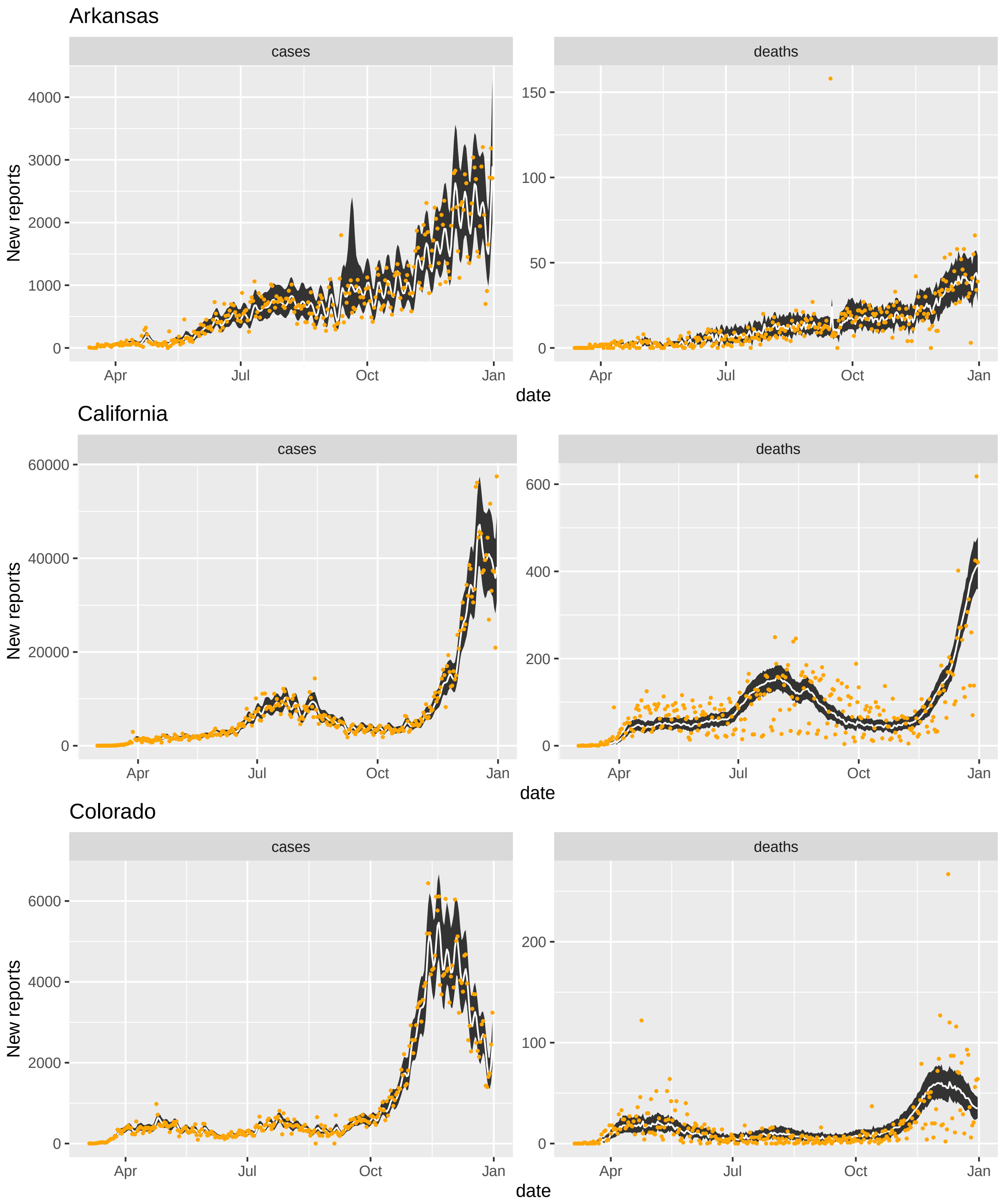
Arkansas
cases
deaths
New reports
4000
3000
2000
1000
0
150
100
50
Apr
Jul
Oct
Jan
date
California
cases
deaths
New reports
60000
40000
20000
600
400
200
Colorado
6000
4000
2000
200
100

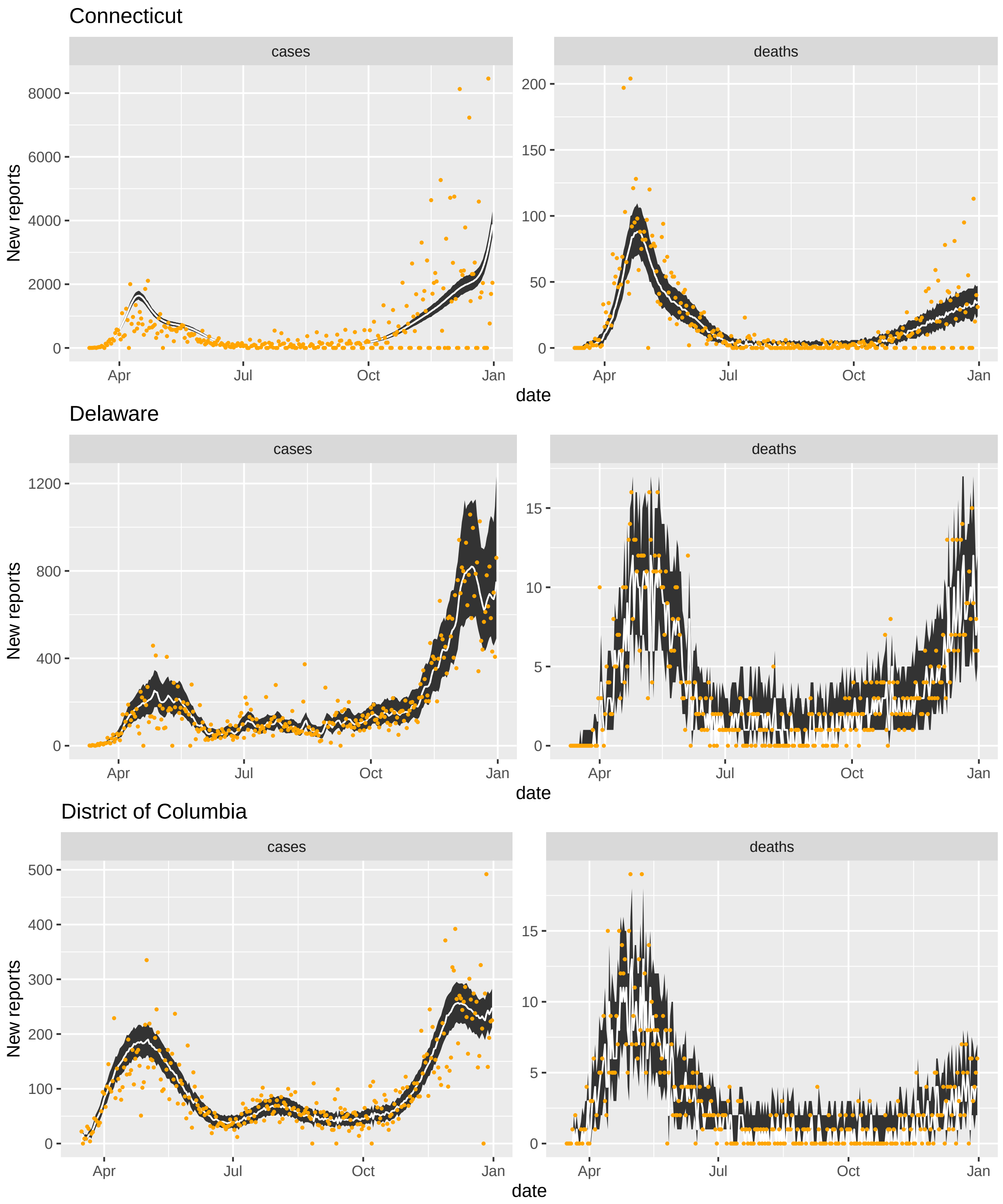
Connecticut
cases
deaths
New reports
0
2000
4000
6000
8000
0
50
100
150
200
Apr
Jul
Oct
Jan
date
Delaware
cases
deaths
New reports
0
400
800
1200
0
5
10
15
Apr
Jul
Oct
Jan
date
District of Columbia
cases
deaths
New reports
0
100
200
300
400
500
0
5
10
15
Apr
Jul
Oct
Jan
date

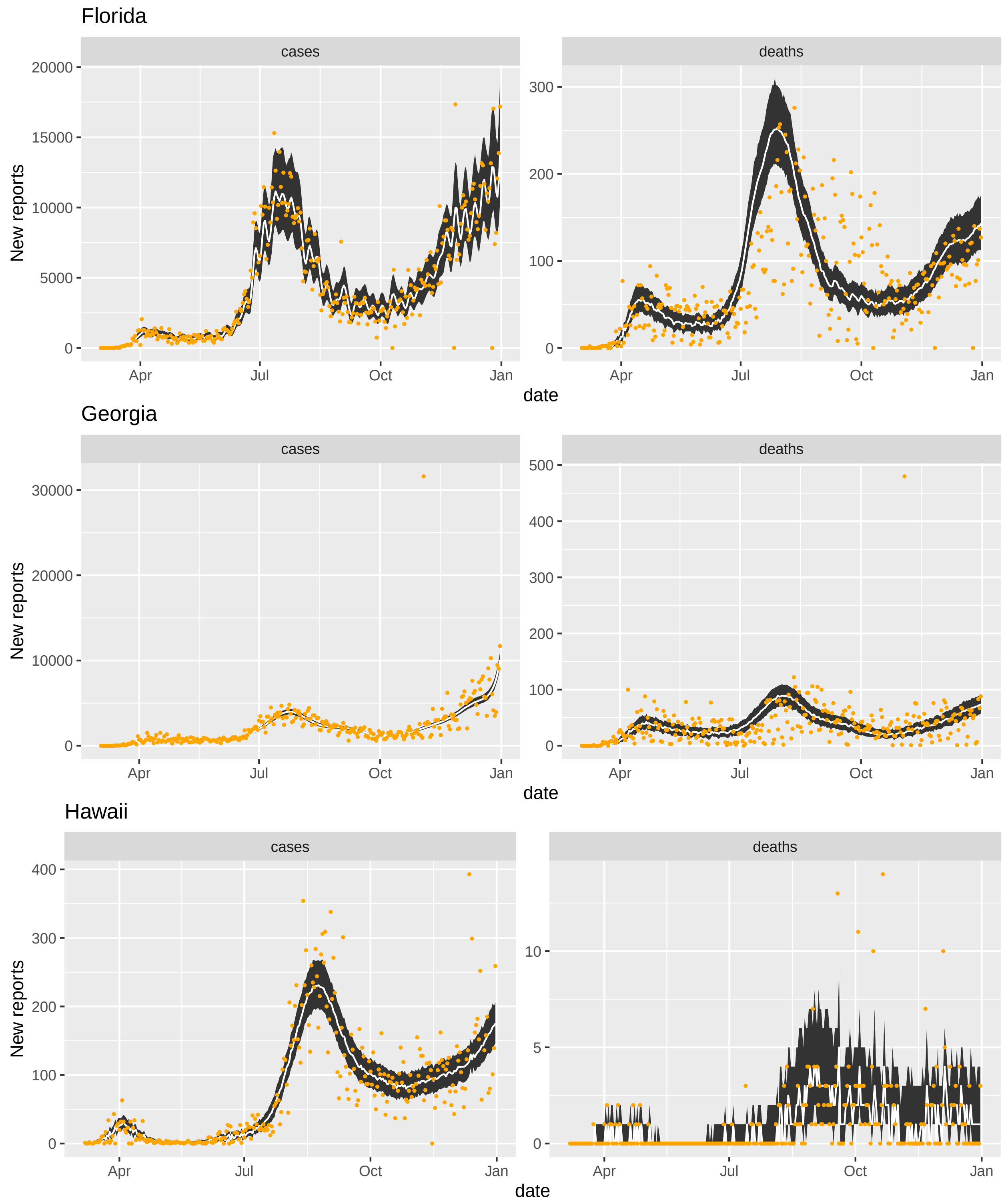
Florida
cases
deaths
New reports
20000
15000
10000
5000
0
300
200
100
0
Apr
Jul
Oct
Jan
date
Georgia
cases
deaths
New reports
30000
20000
10000
0
500
400
300
200
100
0
Apr
Jul
Oct
Jan
date
Hawaii
cases
deaths
New reports
400
300
200
100
0
10
5
0
Apr
Jul
Oct
Jan
date

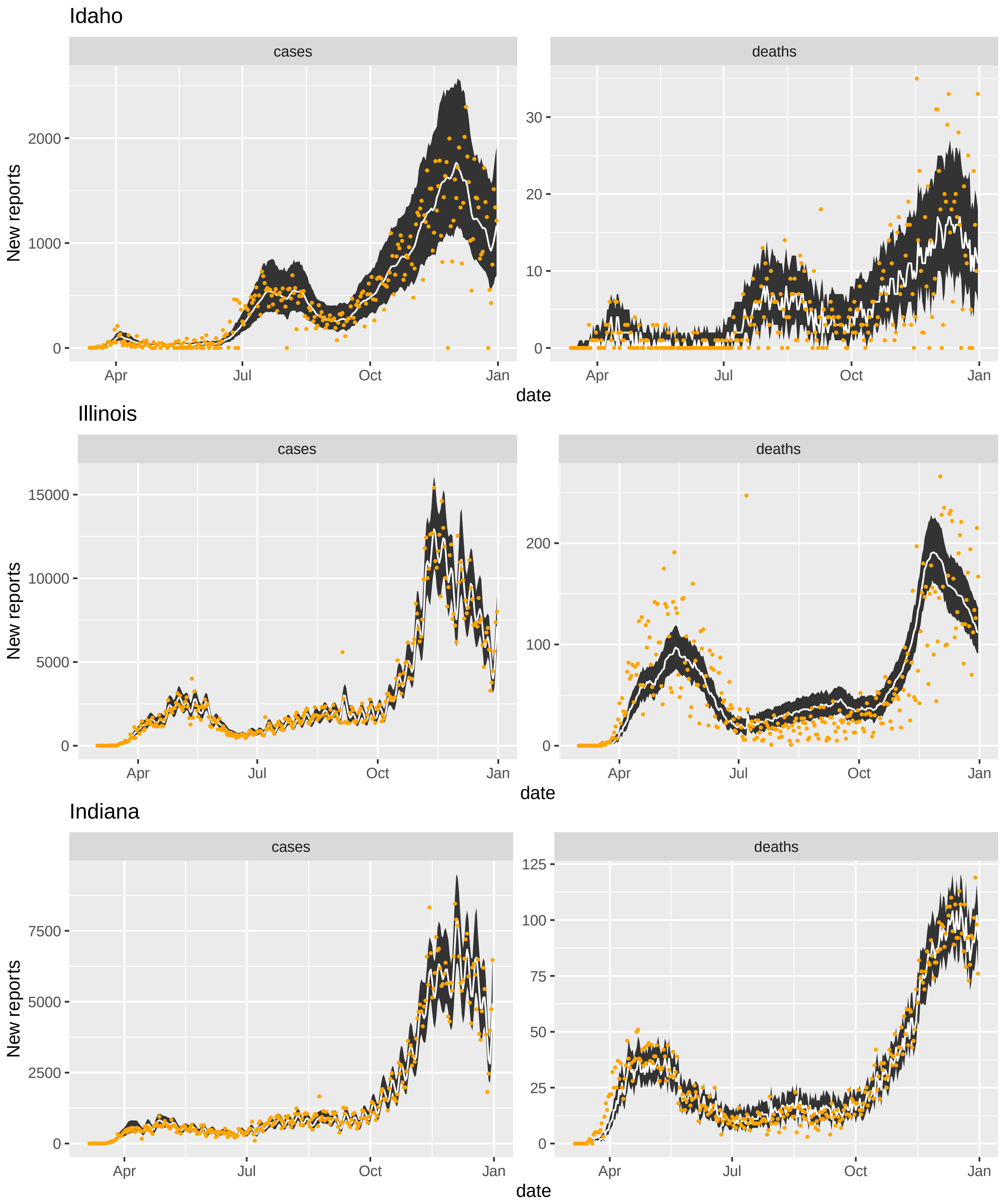
Idaho
cases
deaths
New reports
2000
1000
0
30
20
10
0
Apr
Jul
Oct
Jan
date
Illinois
cases
deaths
15000
10000
5000
0
200
100
0
Apr
Jul
Oct
Jan
date
Indiana
cases
deaths
7500
5000
2500
0
125
100
75
50
25
0
Apr
Jul
Oct
Jan
date

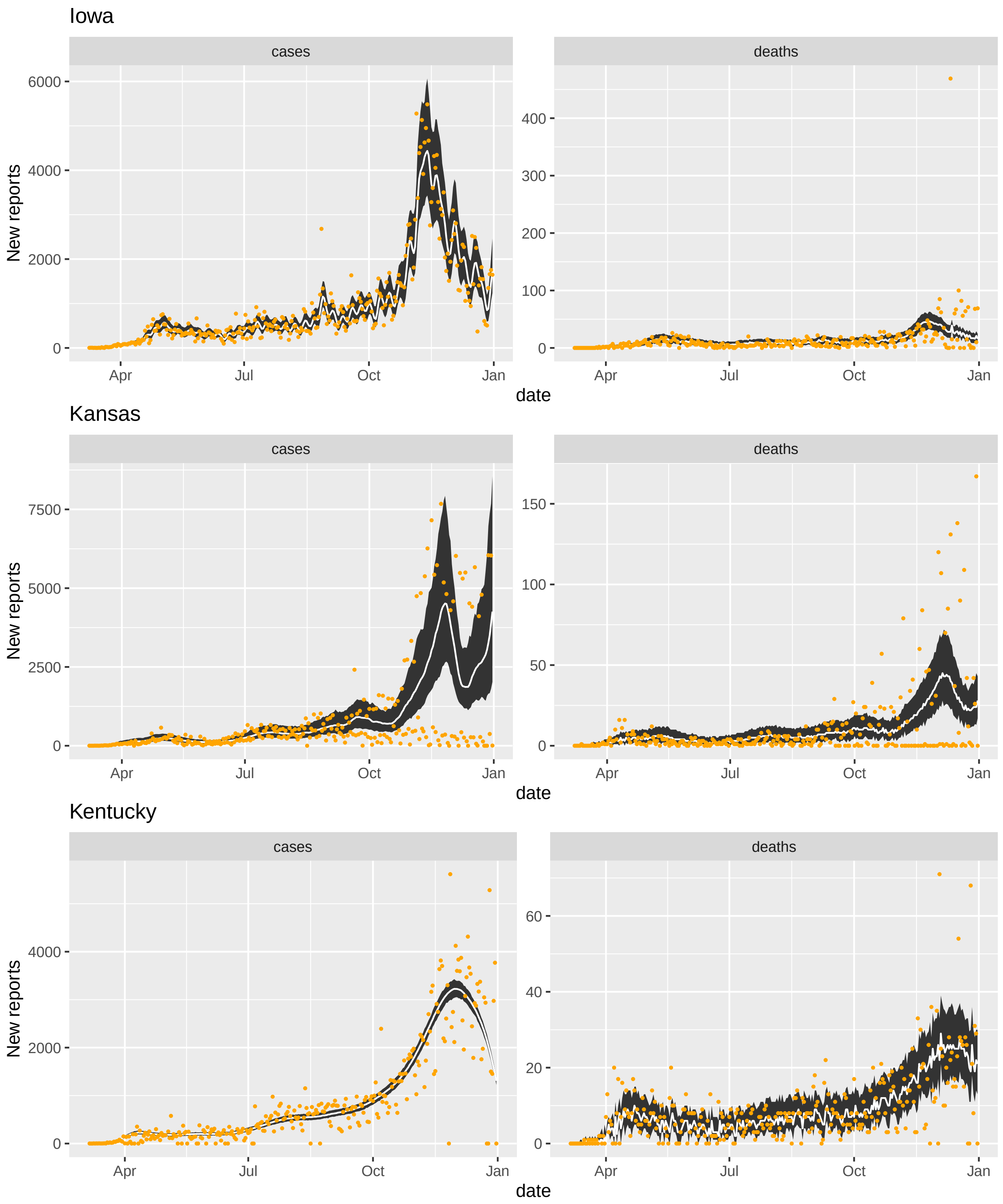
Iowa
cases
deaths
New reports
6000
4000
2000
0
400
300
200
100
0
Apr
Jul
Oct
Jan
date
Kansas
cases
deaths
New reports
7500
5000
2500
0
150
100
50
0
Apr
Jul
Oct
Jan
date
Kentucky
cases
deaths
New reports
4000
2000
0
60
40
20
0
Apr
Jul
Oct
Jan
date

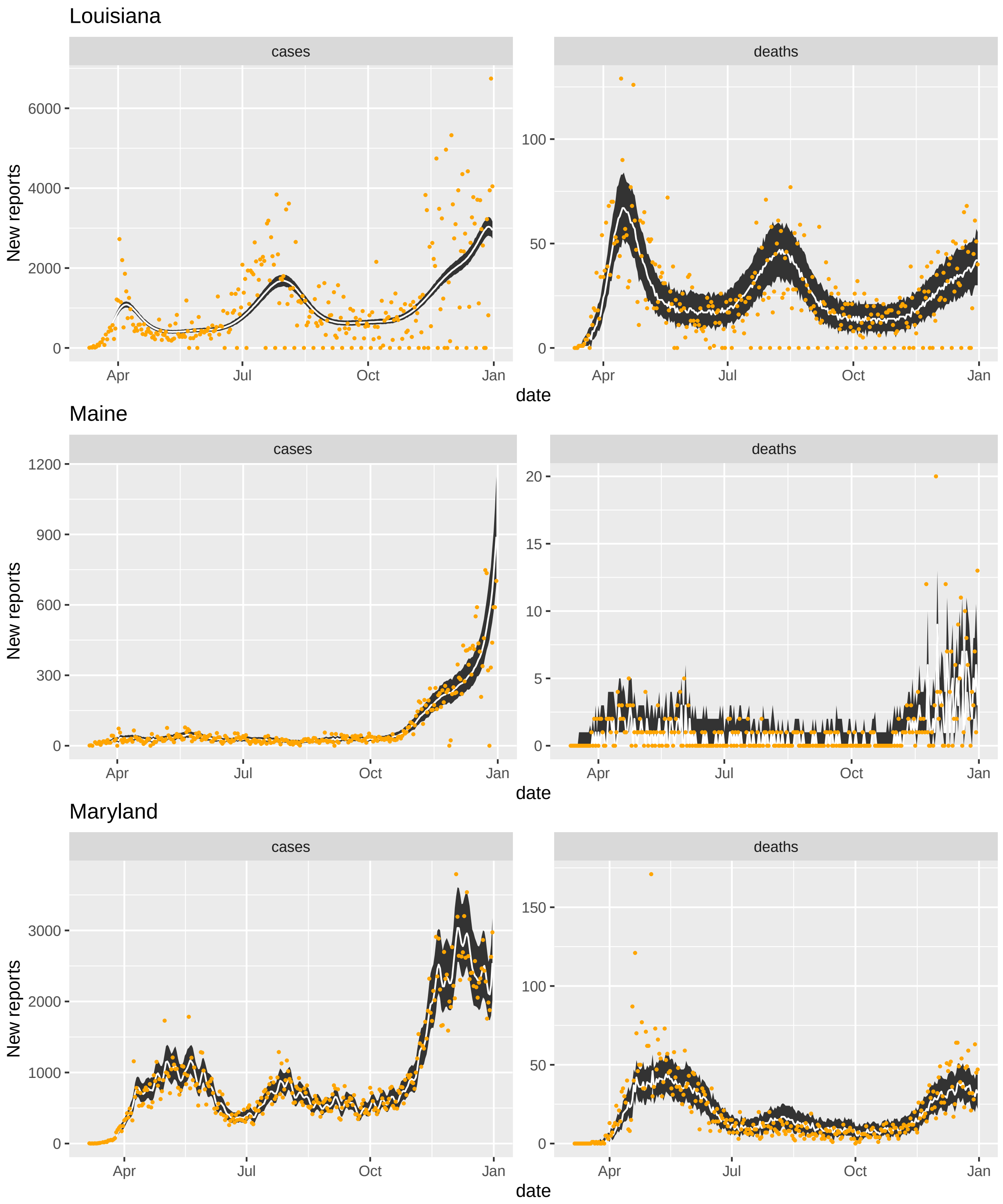
Louisiana
cases
deaths
New reports
6000
4000
2000
0
100
50
0
Apr
Jul
Oct
Jan
date
Maine
cases
deaths
1200
900
600
300
0
20
15
10
5
0
Apr
Jul
Oct
Jan
date
Maryland
cases
deaths
3000
2000
1000
0
150
100
50
0
Apr
Jul
Oct
Jan
date

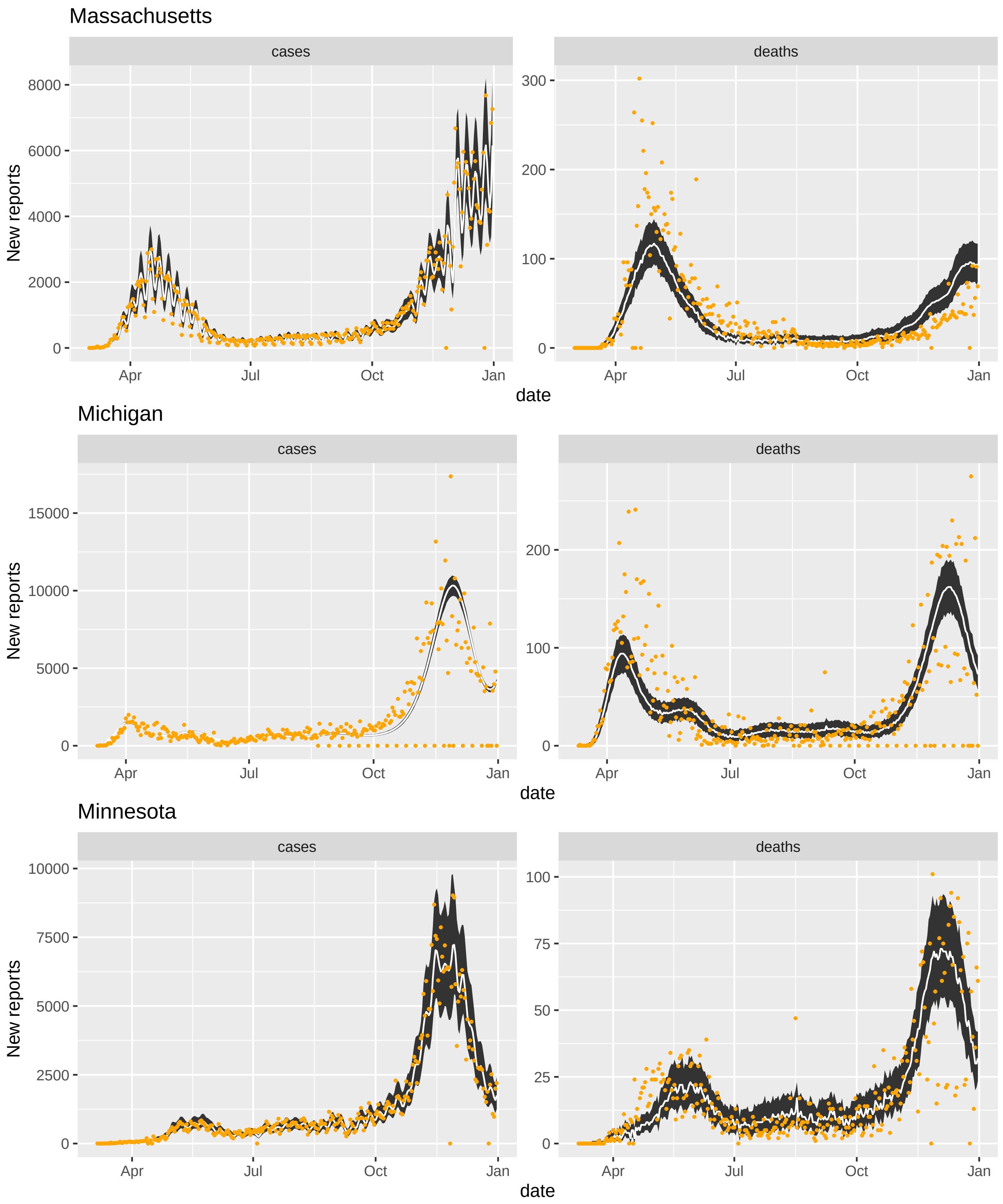
Massachusetts
cases
deaths
New reports
8000
6000
4000
2000
0
300
200
100
0
Apr
Jul
Oct
Jan
date
Michigan
cases
deaths
New reports
15000
10000
5000
0
200
100
0
Apr
Jul
Oct
Jan
date
Minnesota
cases
deaths
New reports
10000
7500
5000
2500
0
100
75
50
25
0
Apr
Jul
Oct
Jan
date

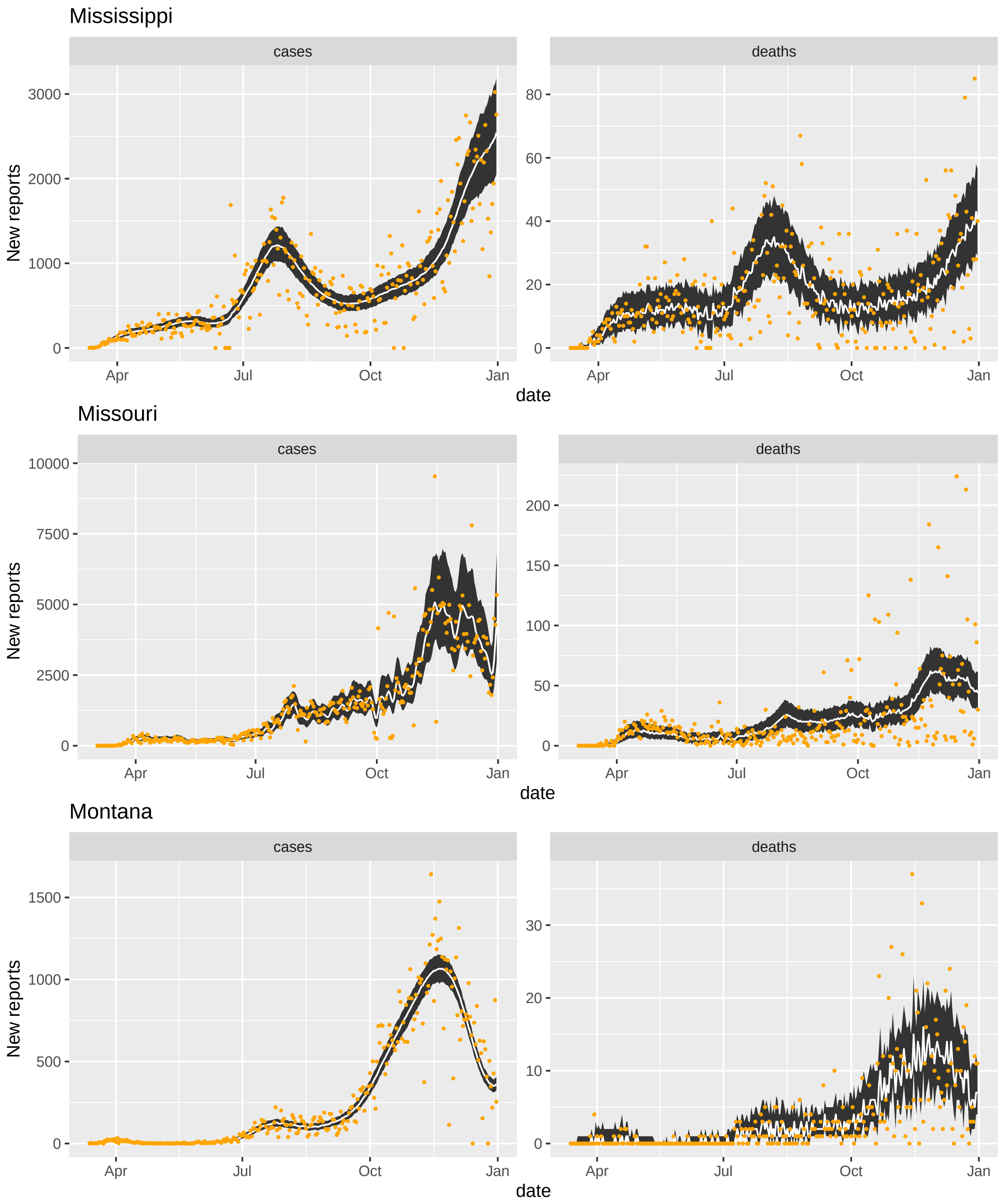
Mississippi
cases
deaths
New reports
3000
2000
1000
0
80
60
40
20
0
Apr
Jul
Oct
Jan
date
Missouri
cases
deaths
New reports
10000
7500
5000
2500
0
200
150
100
50
0
Apr
Jul
Oct
Jan
date
Montana
cases
deaths
New reports
1500
1000
500
0
30
20
10
0
Apr
Jul
Oct
Jan
date

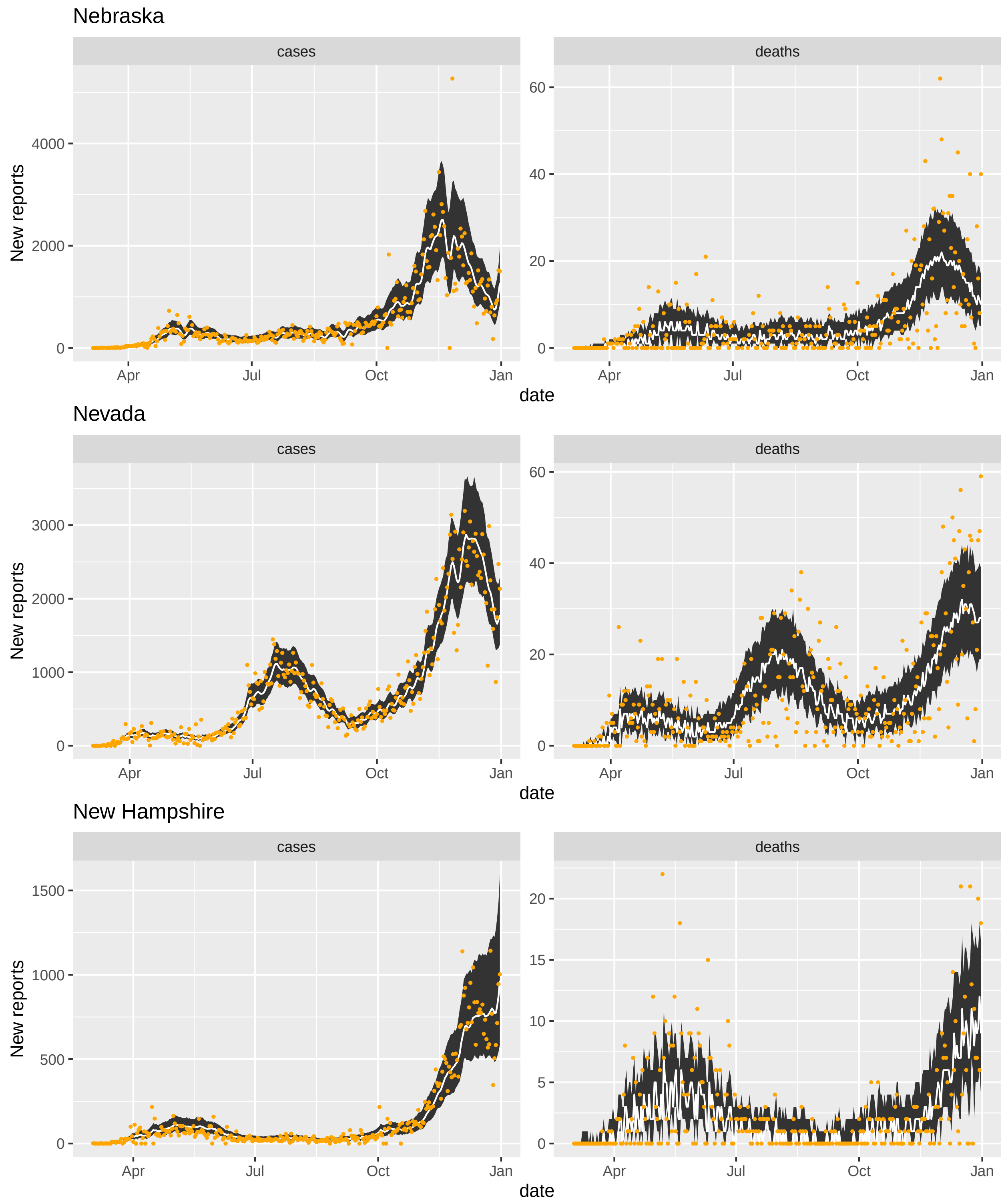
Nebraska
cases
deaths
New reports
4000
2000
0
60
40
20
Apr
Jul
Oct
Jan
date
Nevada
cases
deaths
New reports
3000
2000
1000
0
60
40
20
Apr
Jul
Oct
Jan
date
New Hampshire
cases
deaths
New reports
1500
1000
500
0
20
15
10
5
Apr
Jul
Oct
Jan
date

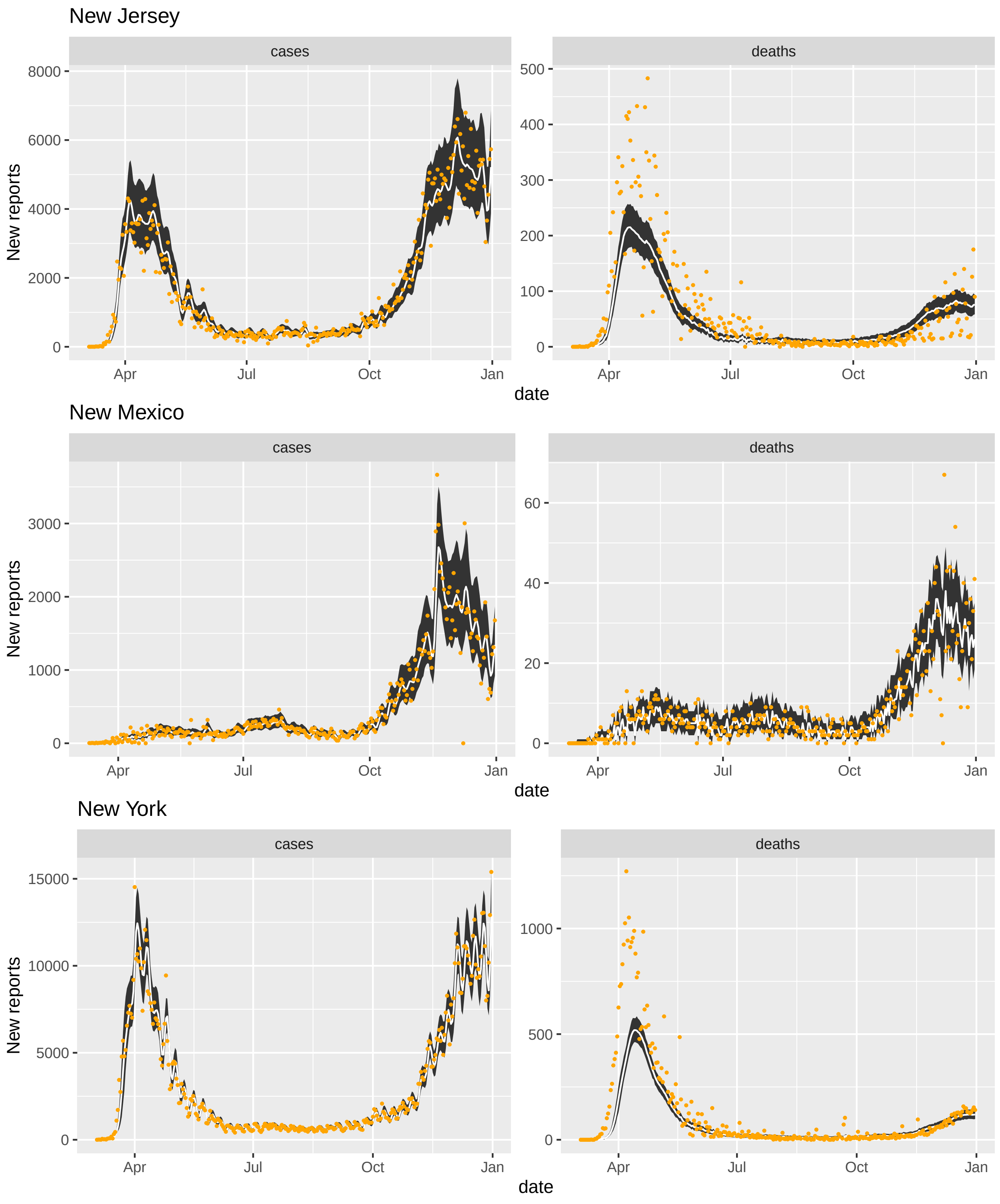
New Jersey
cases
deaths
New reports
8000
6000
4000
2000
0
500
400
300
200
100
0
Apr
Jul
Oct
Jan
date
New Mexico
cases
deaths
New reports
3000
2000
1000
0
60
40
20
0
Apr
Jul
Oct
Jan
date
New York
cases
deaths
New reports
15000
10000
5000
0
1000
500
0
Apr
Jul
Oct
Jan
date

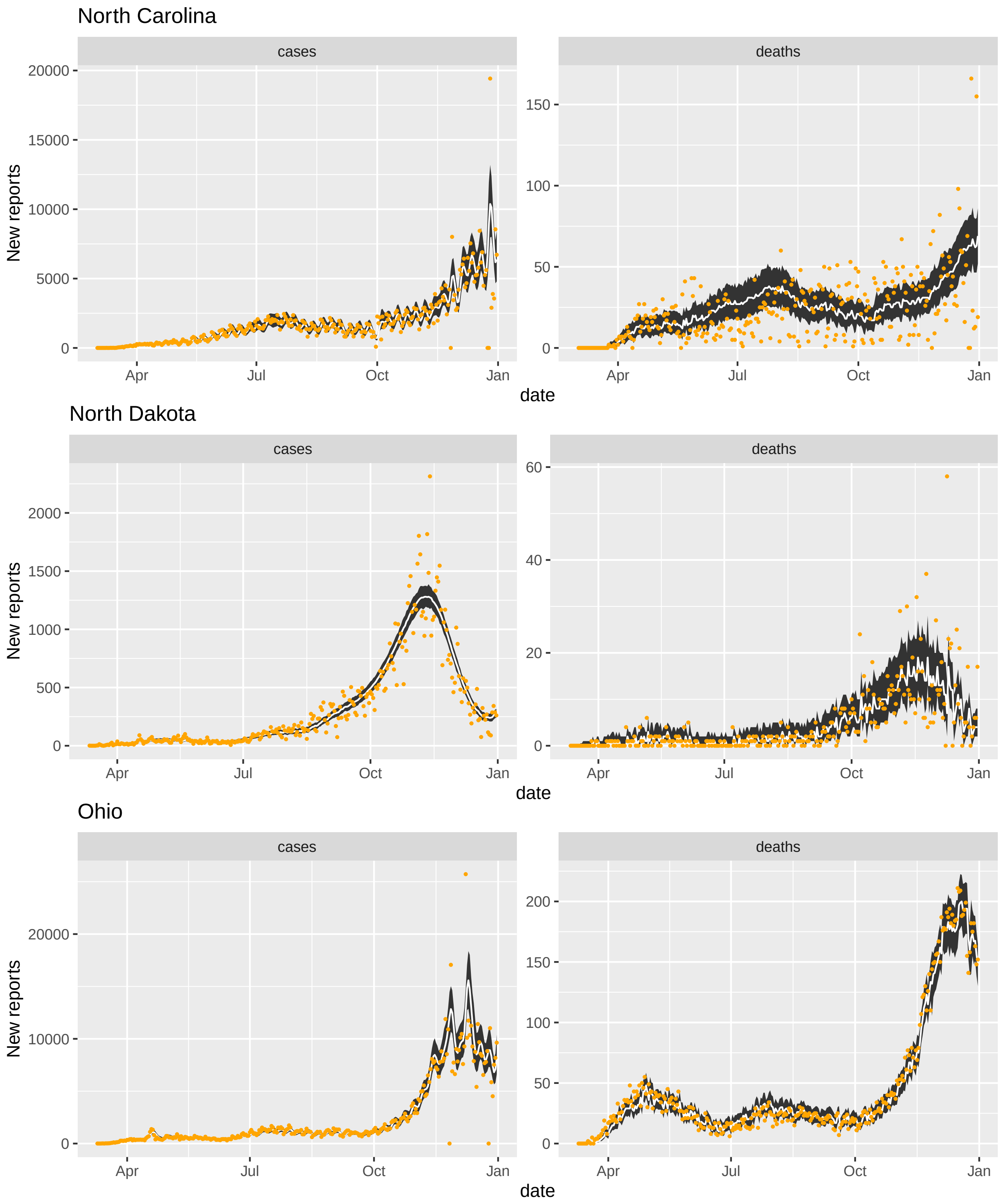
North Carolina
cases
deaths
New reports
20000
15000
10000
5000
0
150
100
50
0
Apr
Jul
Oct
Jan
date
North Dakota
cases
deaths
New reports
2000
1500
1000
500
0
60
40
20
0
Apr
Jul
Oct
Jan
date
Ohio
cases
deaths
New reports
20000
10000
0
200
150
100
50
0
Apr
Jul
Oct
Jan
date

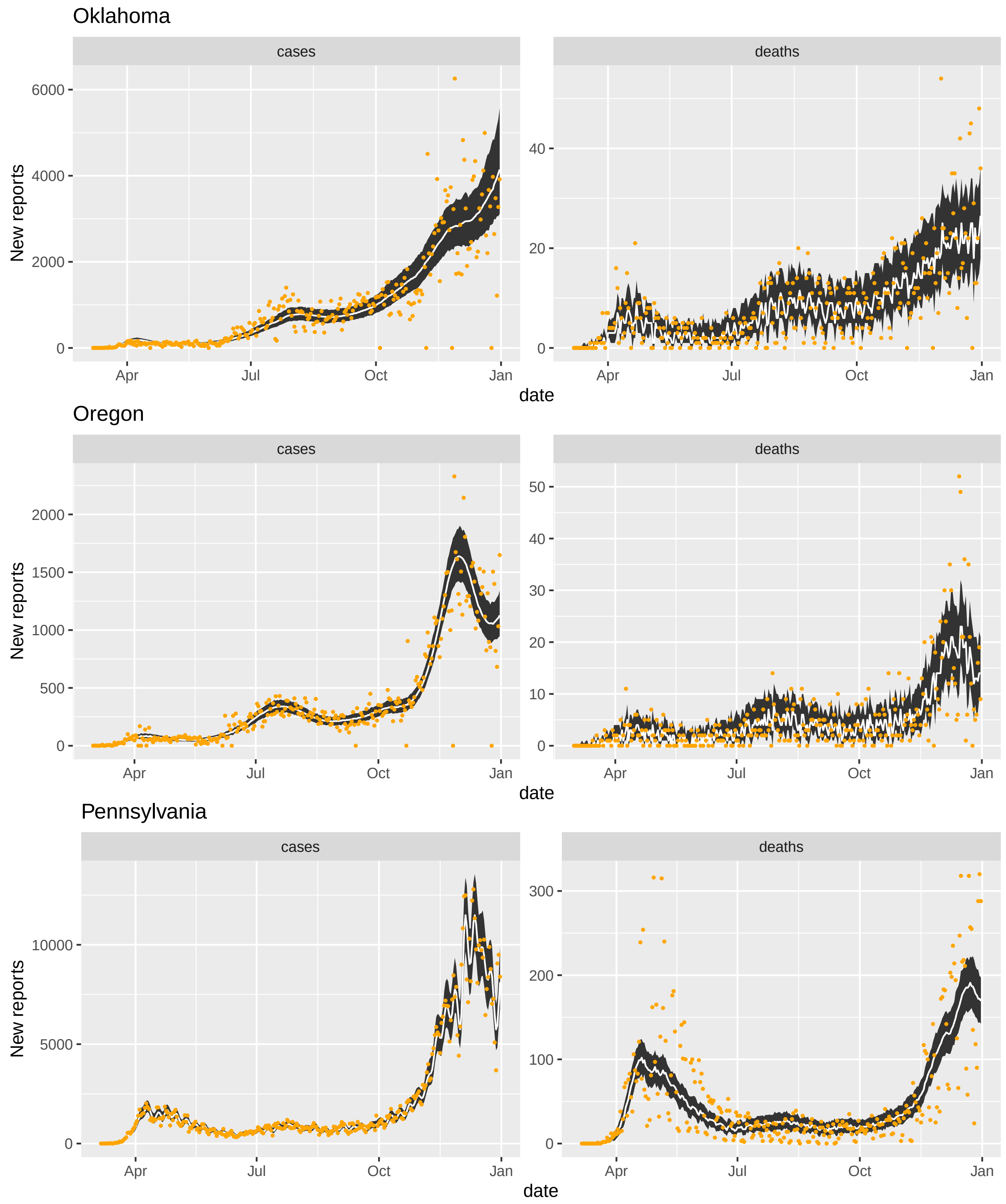

Oklahoma
cases
deaths
New reports
6000
4000
2000
0
40
20
0
Apr
Jul
Oct
Jan
date
Oregon
cases
deaths
New reports
2000
1500
1000
500
0
50
40
30
20
10
0
Apr
Jul
Oct
Jan
date
Pennsylvania
cases
deaths
New reports
10000
5000
0
300
200
100
0
Apr
Jul
Oct
Jan
date

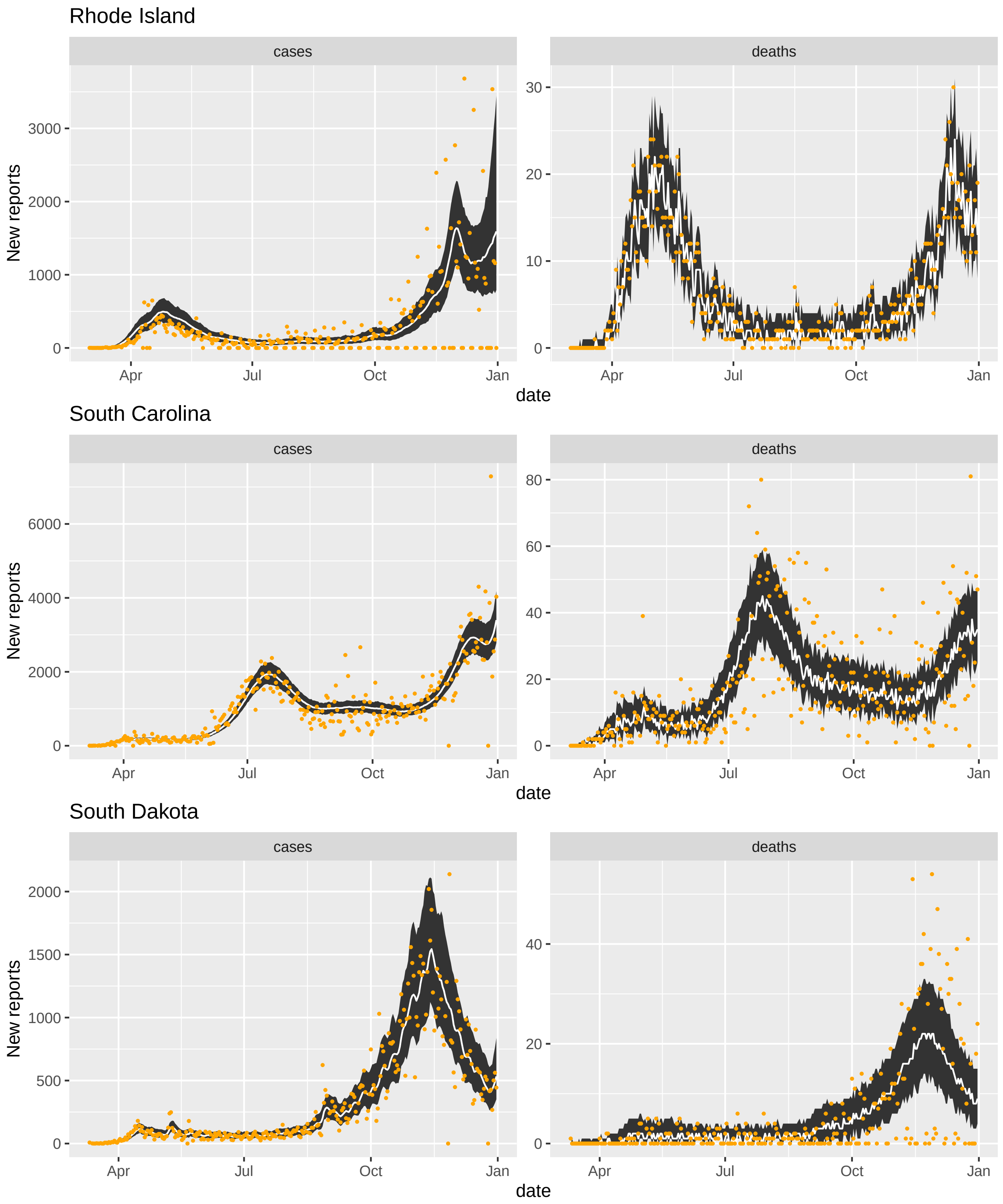

Rhode Island
cases
deaths
New reports
3000
2000
1000
0
30
20
10
Apr
Jul
Oct
Jan
date
South Carolina
cases
deaths
New reports
6000
4000
2000
80
60
40
20
South Dakota
cases
deaths
New reports
2000
1500
1000
500

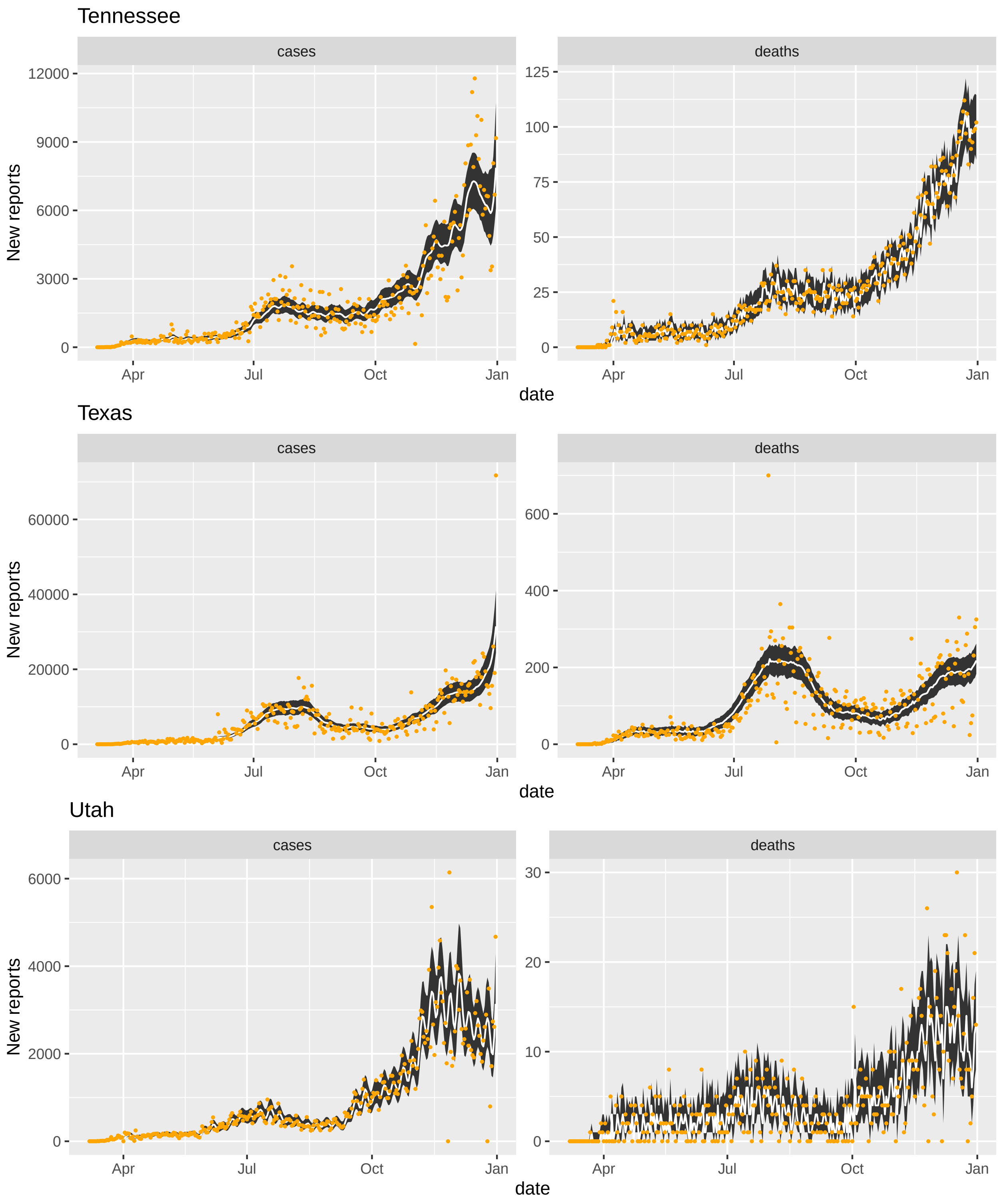
Tennessee
cases
deaths
New reports
12000
9000
6000
3000
0
125
100
75
50
25
0
Apr
Jul
Oct
Jan
date
Texas
cases
deaths
New reports
60000
40000
20000
0
600
400
200
0
Apr
Jul
Oct
Jan
date
Utah
cases
deaths
New reports
6000
4000
2000
0
30
20
10
0
Apr
Jul
Oct
Jan
date

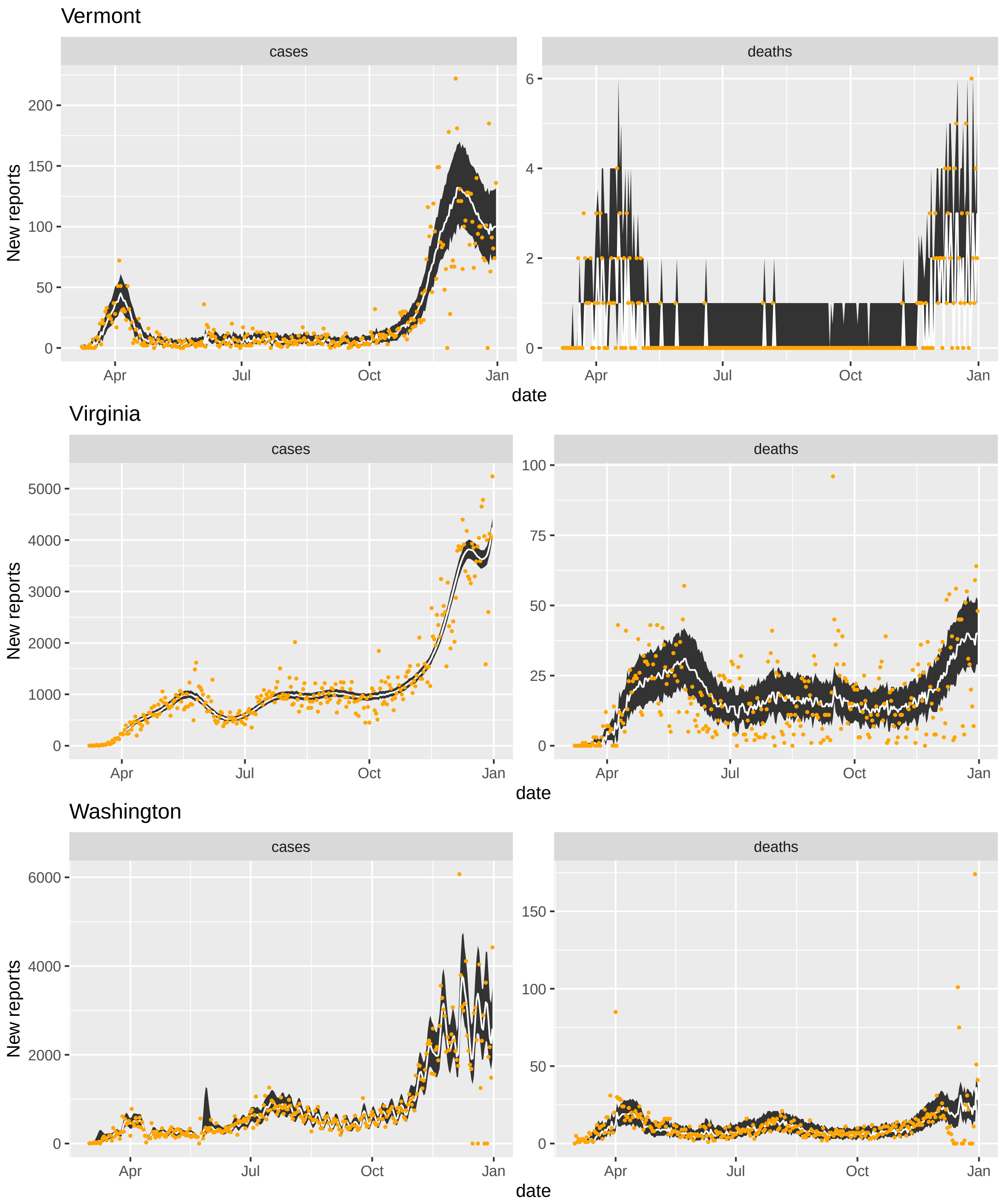
Vermont
cases
deaths
New reports
date
Virginia
cases
deaths
New reports
date
Washington
cases
deaths
New reports
date
Apr
Jul
Oct
Jan

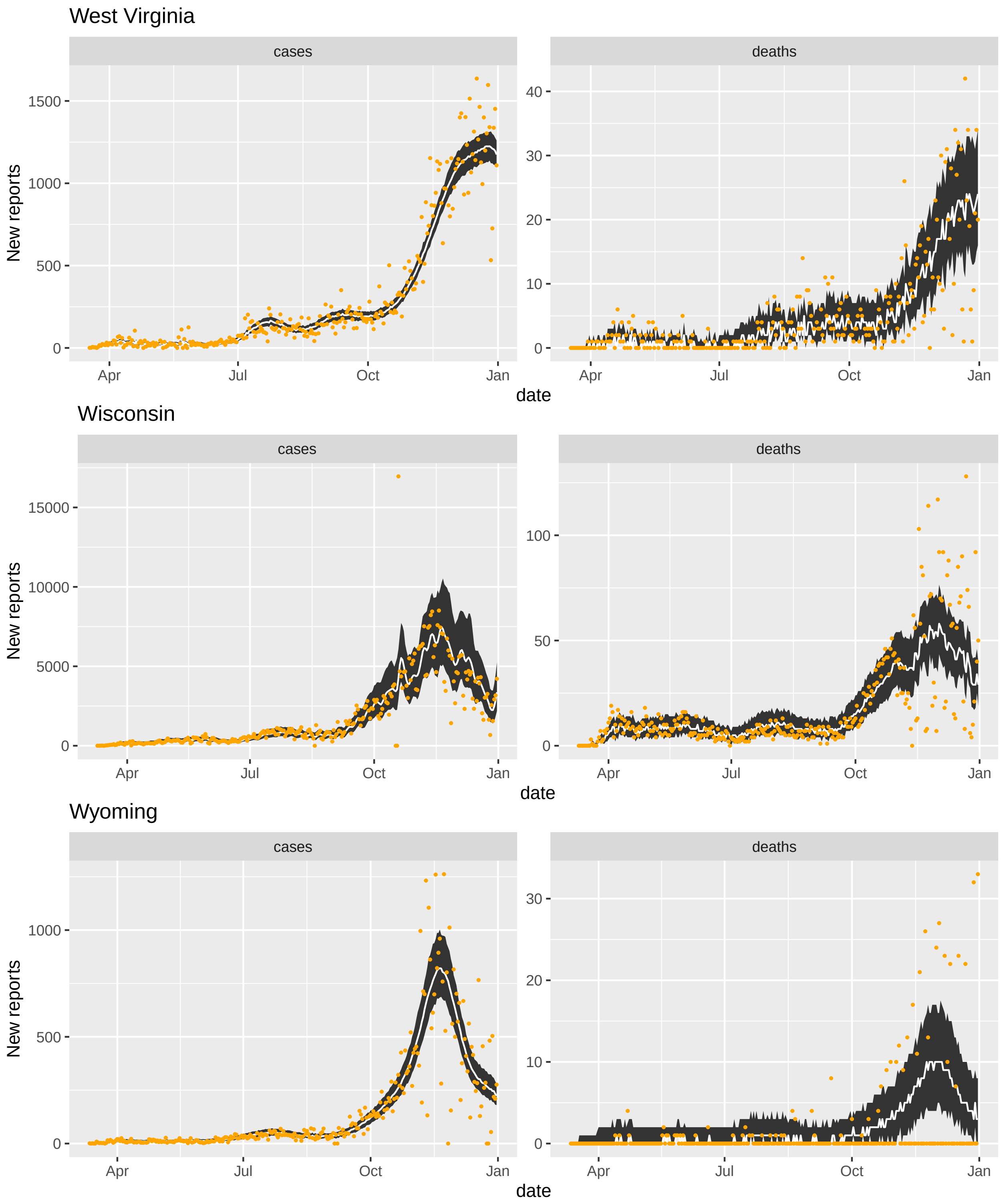
West Virginia
cases
deaths
New reports
1500
1000
500
0
40
30
20
10
0
Apr
Jul
Oct
Jan
date
Wisconsin
cases
deaths
New reports
15000
10000
5000
0
100
50
0
Apr
Jul
Oct
Jan
date
Wyoming
cases
deaths
New reports
1000
500
0
30
20
10
0
Apr
Jul
Oct
Jan
date

D. Time series of mobility, estimated latent trend, and $\mathcal{R}_e$

The following plots show the estimated latent trend, relative mobility, and estimated $\mathcal{R}_e$ over time for each state. The latent trend is estimated using the maximum likelihood parameter estimates for the g_i coefficients of the B-spline (see Materials and Methods in main text).

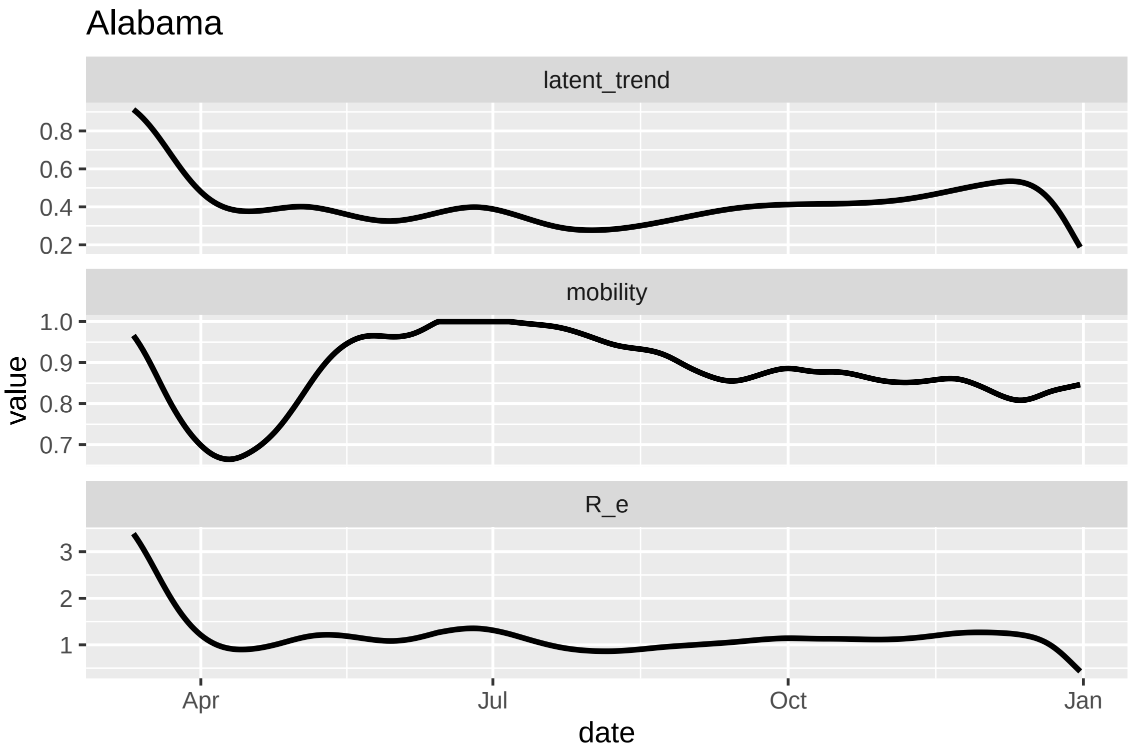

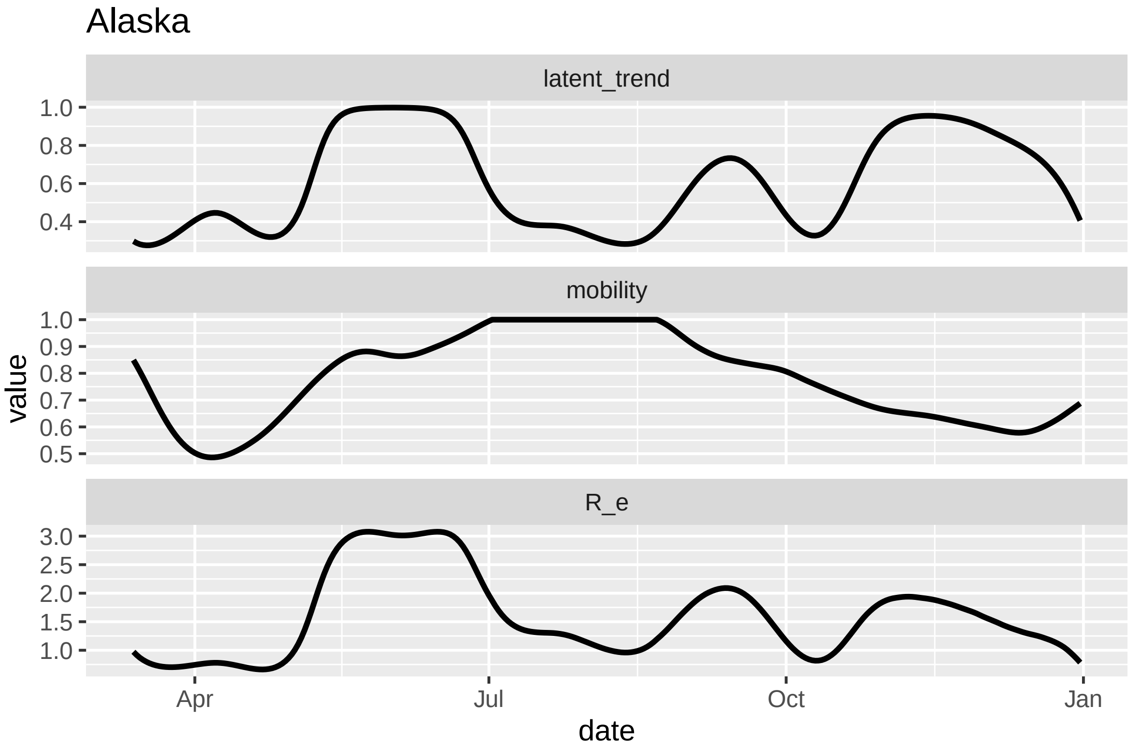

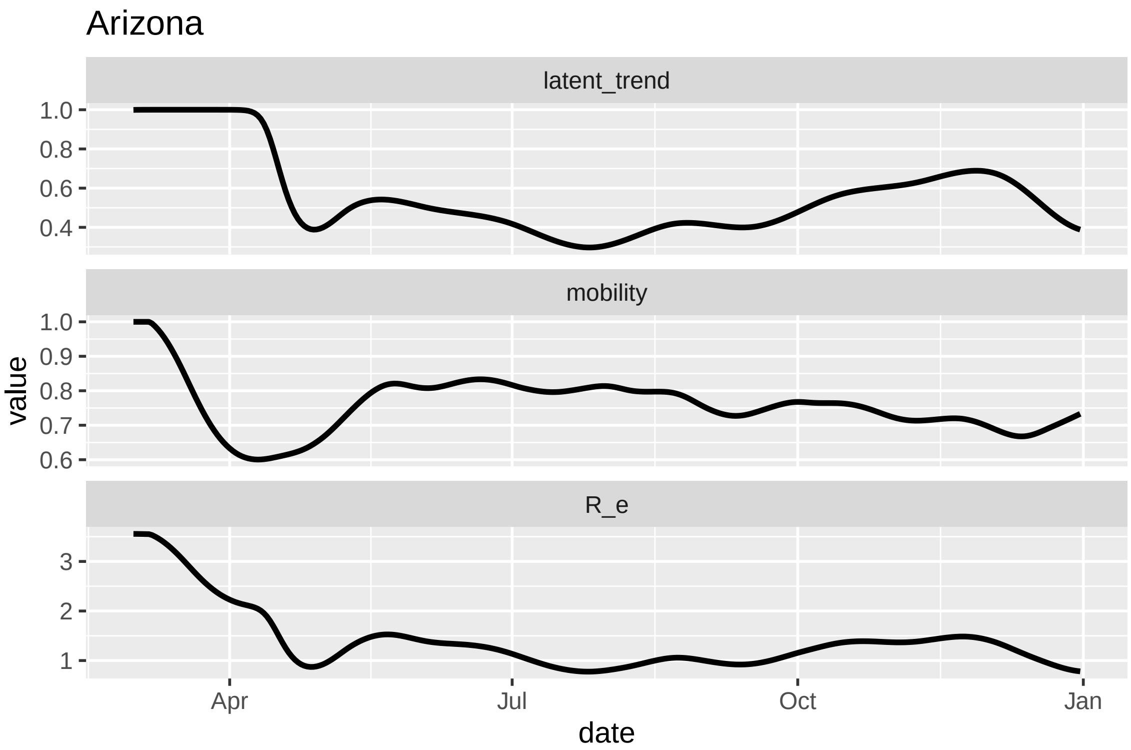
Arizona
latent_trend
mobility
R_e
value
date
Apr
Jul
Oct
Jan

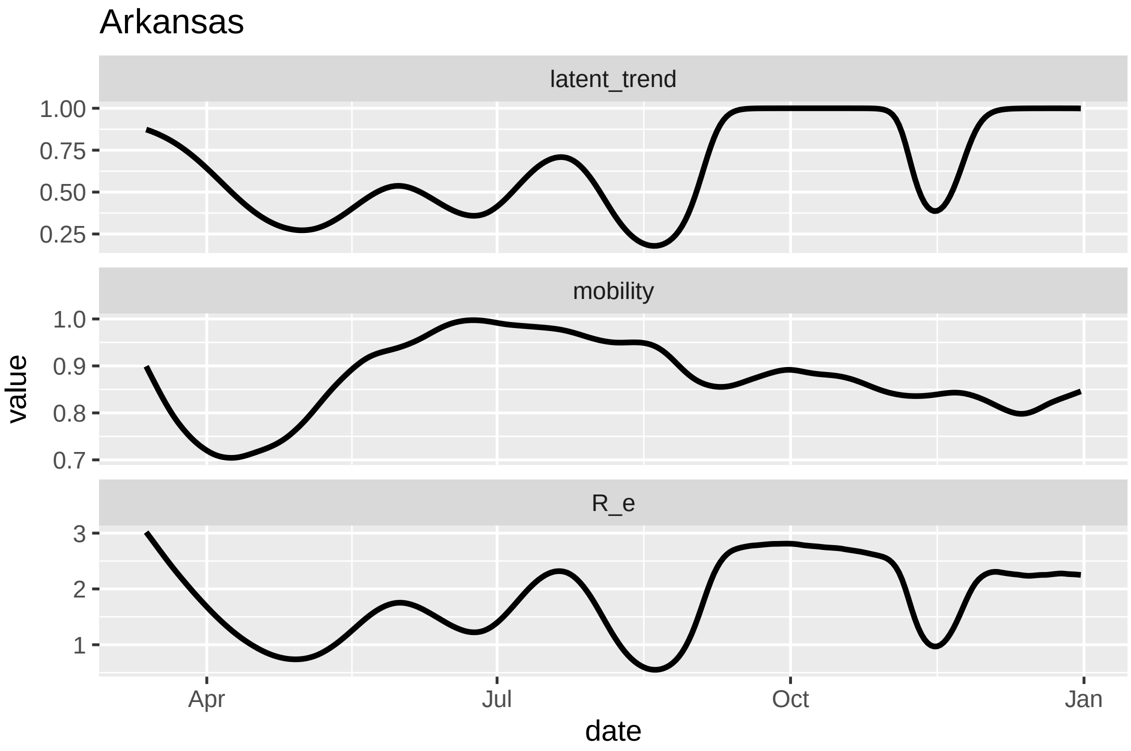
Arkansas
latent_trend
mobility
R_e
value
date
Apr
Jul
Oct
Jan

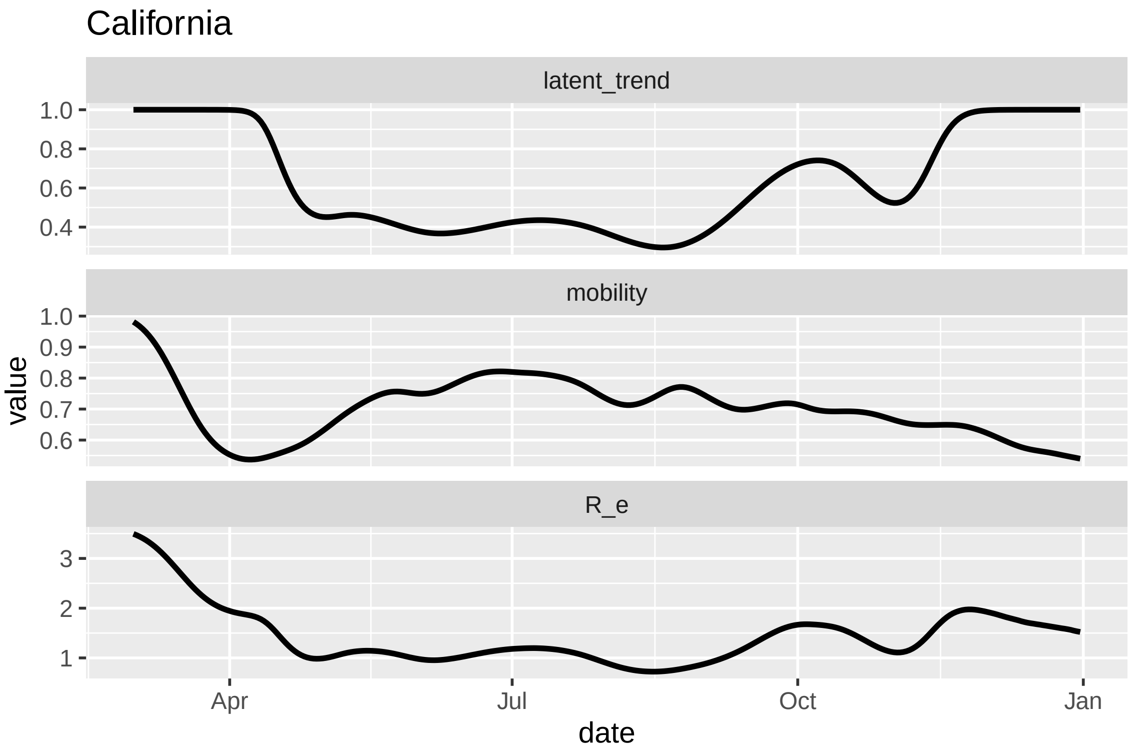
California
latent_trend
1.0
0.8
0.6
0.4
mobility
1.0
0.9
0.8
0.7
0.6
value
R_e
3
2
1
Apr
Jul
Oct
Jan
date

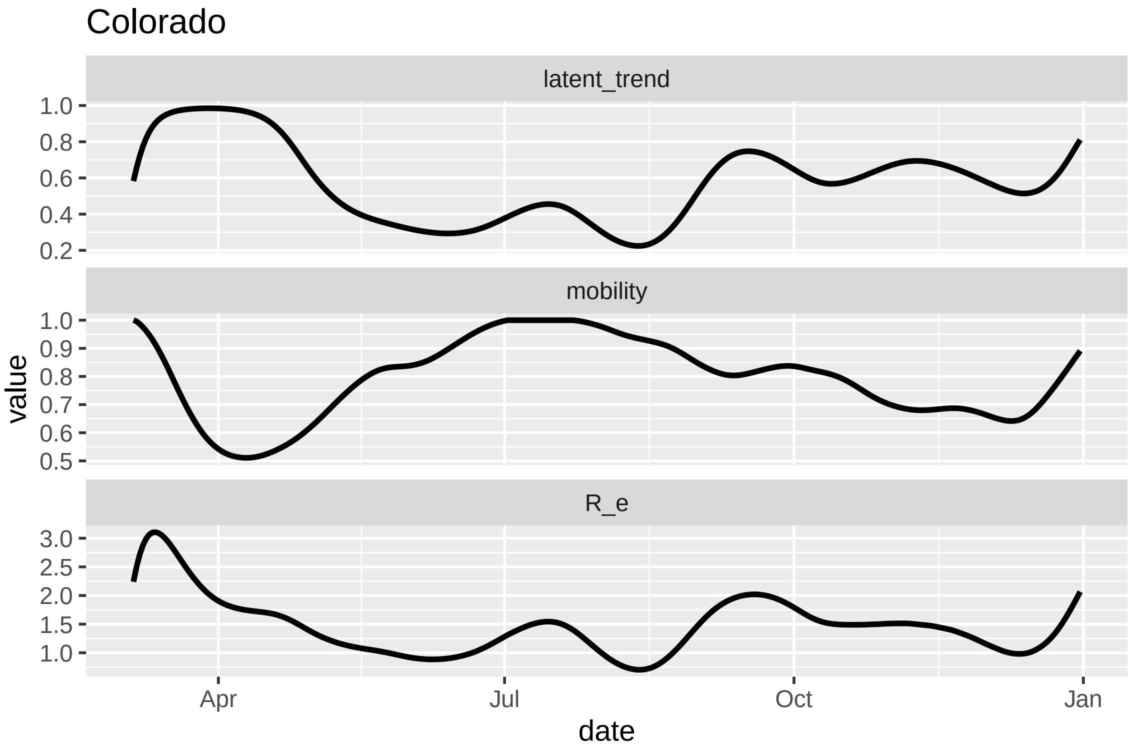
Colorado
latent_trend
1.0
0.8
0.6
0.4
0.2
mobility
1.0
0.9
0.8
0.7
0.6
0.5
value
R_e
3.0
2.5
2.0
1.5
1.0
Apr
Jul
Oct
Jan
date

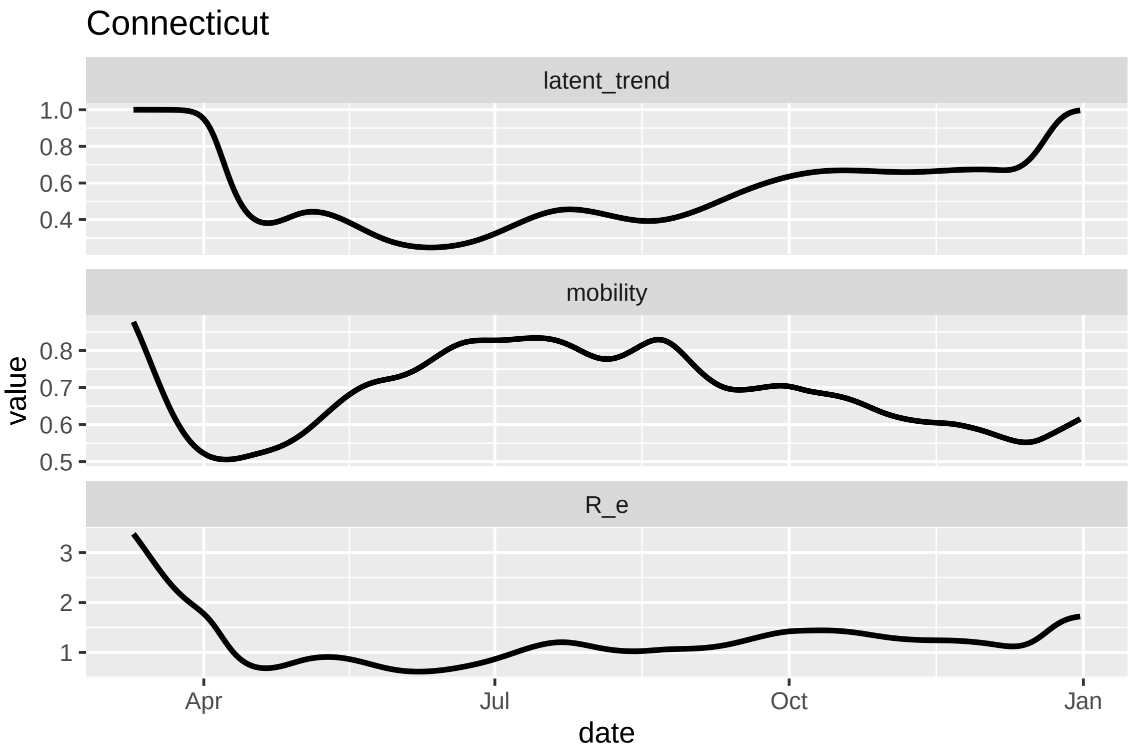
Connecticut
latent_trend
1.0
0.8
0.6
0.4
mobility
0.8
0.7
0.6
0.5
value
R_e
3
2
1
Apr
Jul
Oct
Jan
date

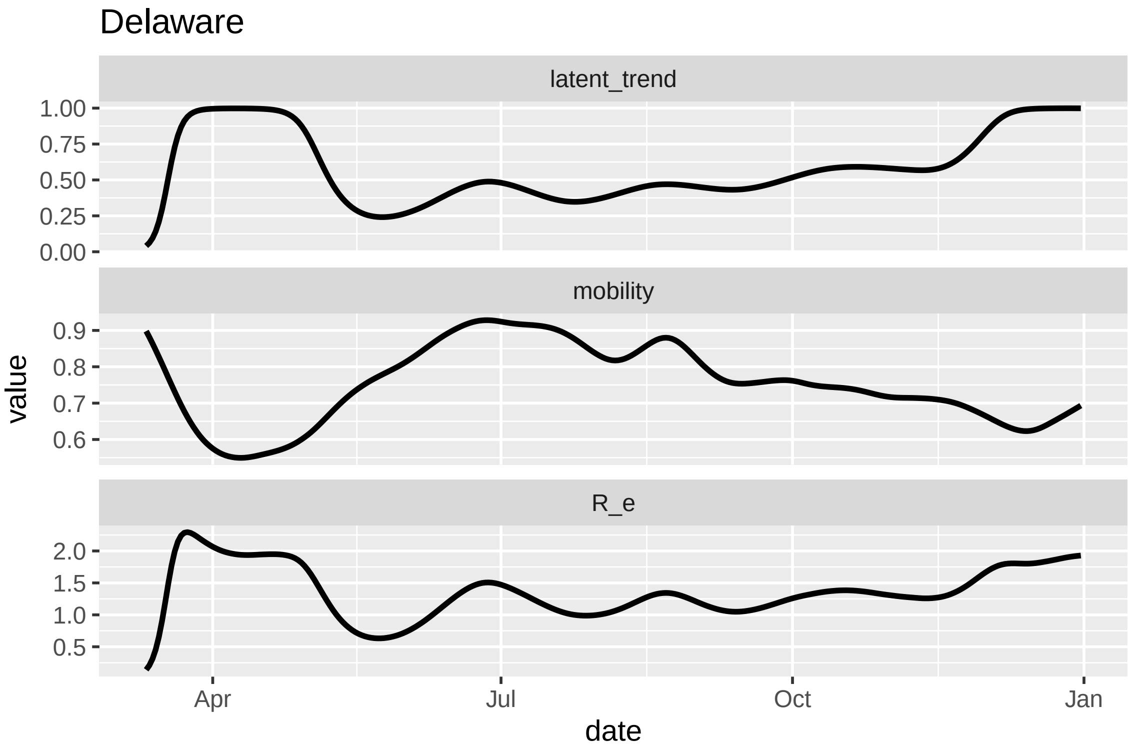
Delaware
latent_trend
1.00
0.75
0.50
0.25
0.00
mobility
0.9
0.8
0.7
0.6
value
R_e
2.0
1.5
1.0
0.5
Apr
Jul
Oct
Jan
date

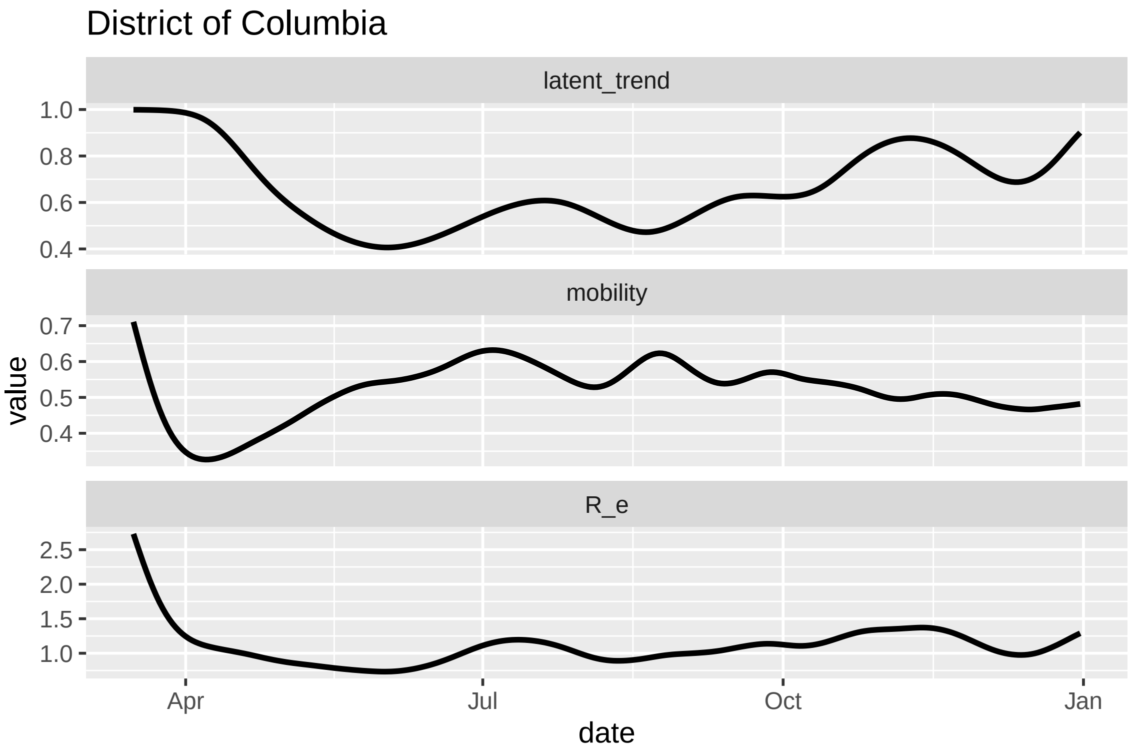
District of Columbia
latent_trend
1.0
0.8
0.6
0.4
mobility
0.7
0.6
0.5
0.4
R_e
2.5
2.0
1.5
1.0
value
Apr
Jul
Oct
Jan
date

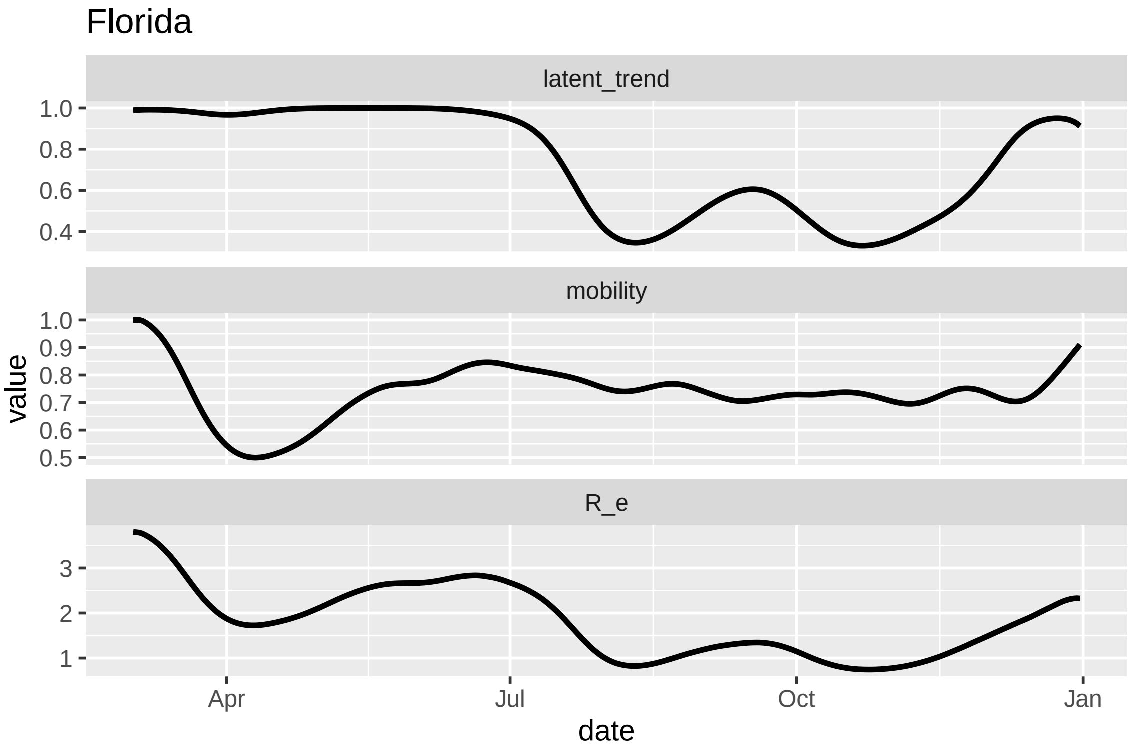
Florida
latent_trend
1.0
0.8
0.6
0.4
mobility
1.0
0.9
0.8
0.7
0.6
0.5
R_e
3
2
1
value
Apr
Jul
Oct
Jan
date

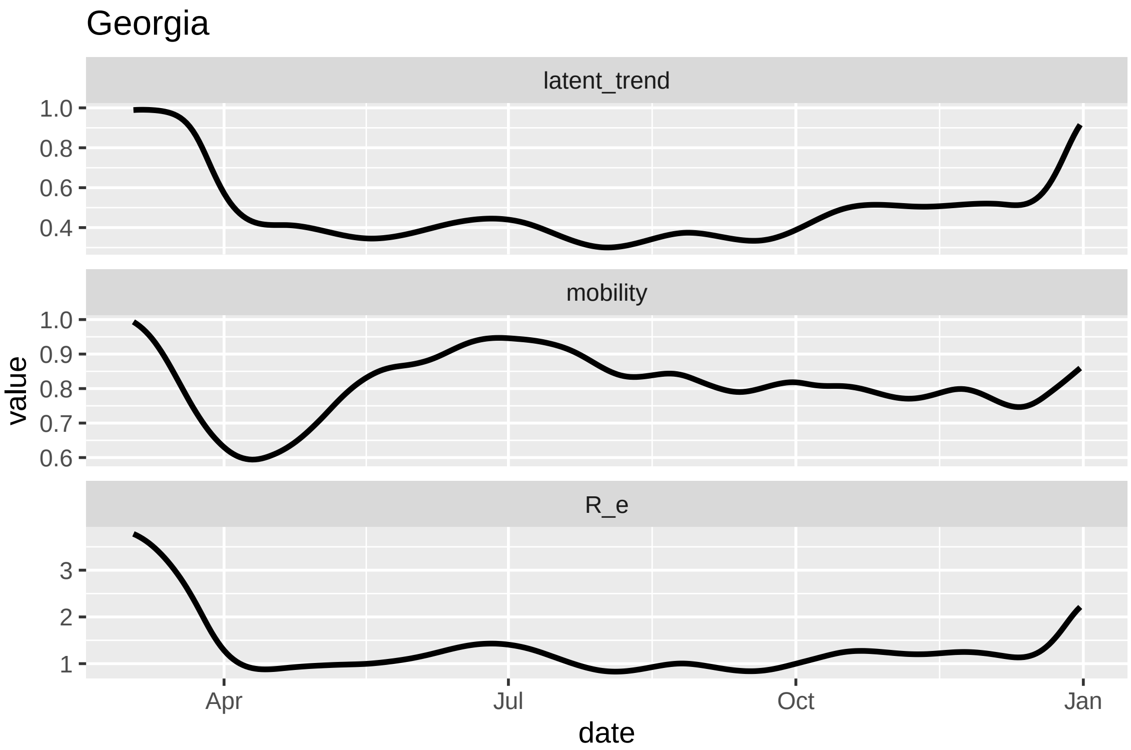
Georgia
latent_trend
1.0
0.8
0.6
0.4
mobility
1.0
0.9
0.8
0.7
0.6
value
R_e
3
2
1
Apr
Jul
Oct
Jan
date

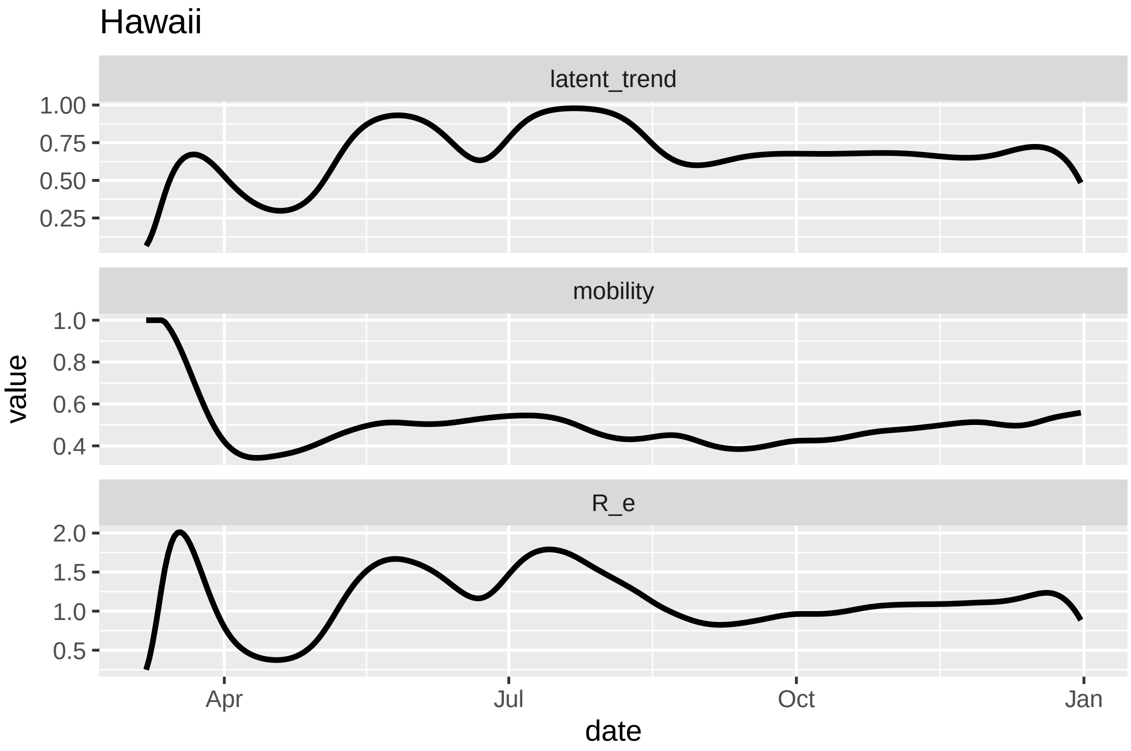
Hawaii
latent_trend
1.00
0.75
0.50
0.25
mobility
1.0
0.8
0.6
0.4
value
R_e
2.0
1.5
1.0
0.5
Apr
Jul
Oct
Jan
date

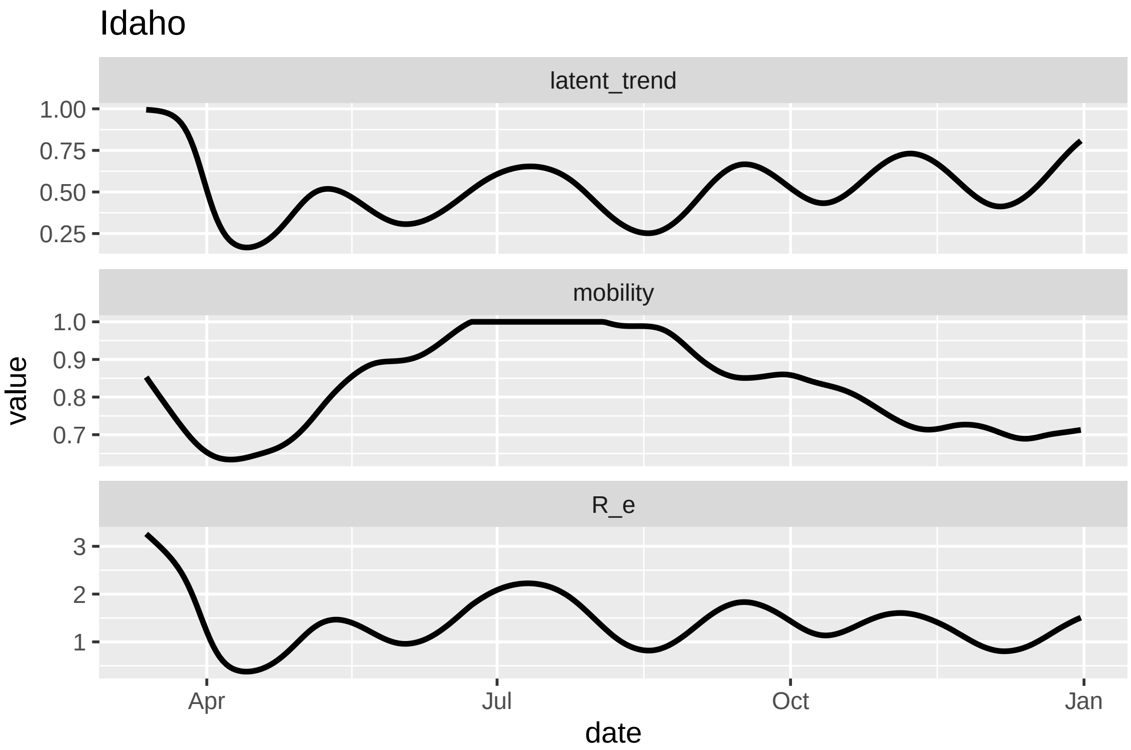
Idaho
latent_trend
1.00
0.75
0.50
0.25
mobility
1.0
0.9
0.8
0.7
value
R_e
3
2
1
Apr
Jul
Oct
Jan
date

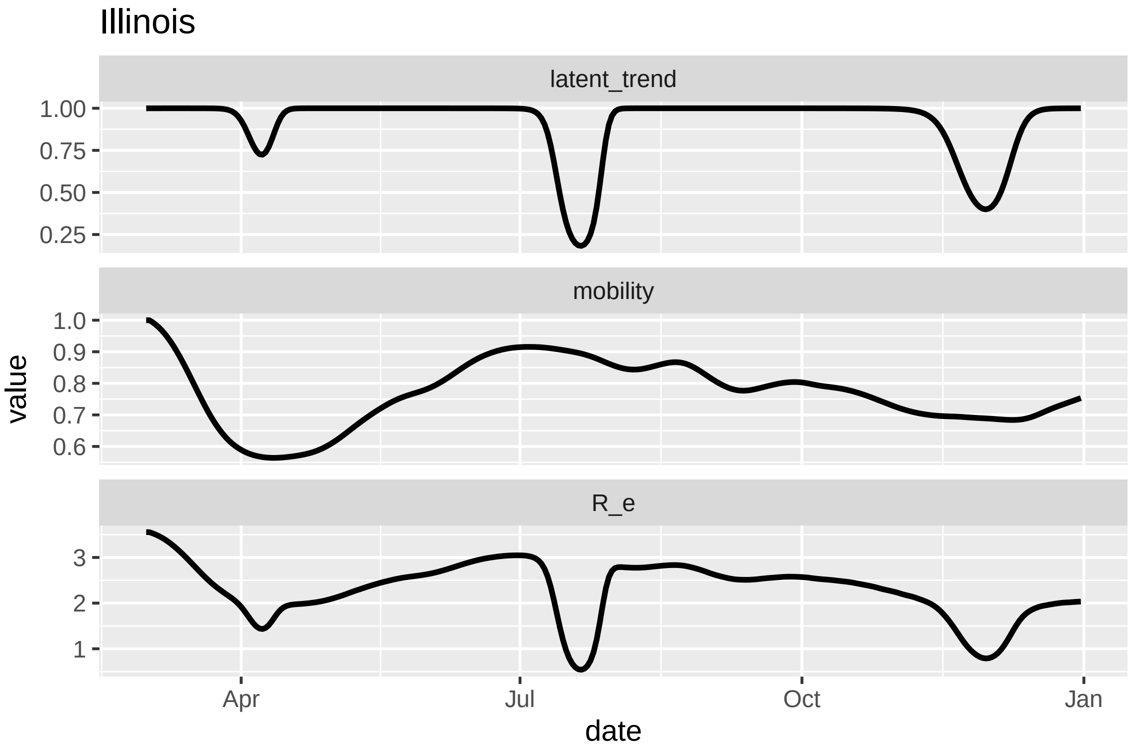
Illinois
latent_trend
1.00
0.75
0.50
0.25
mobility
1.0
0.9
0.8
0.7
0.6
value
R_e
3
2
1
Apr
Jul
Oct
Jan
date

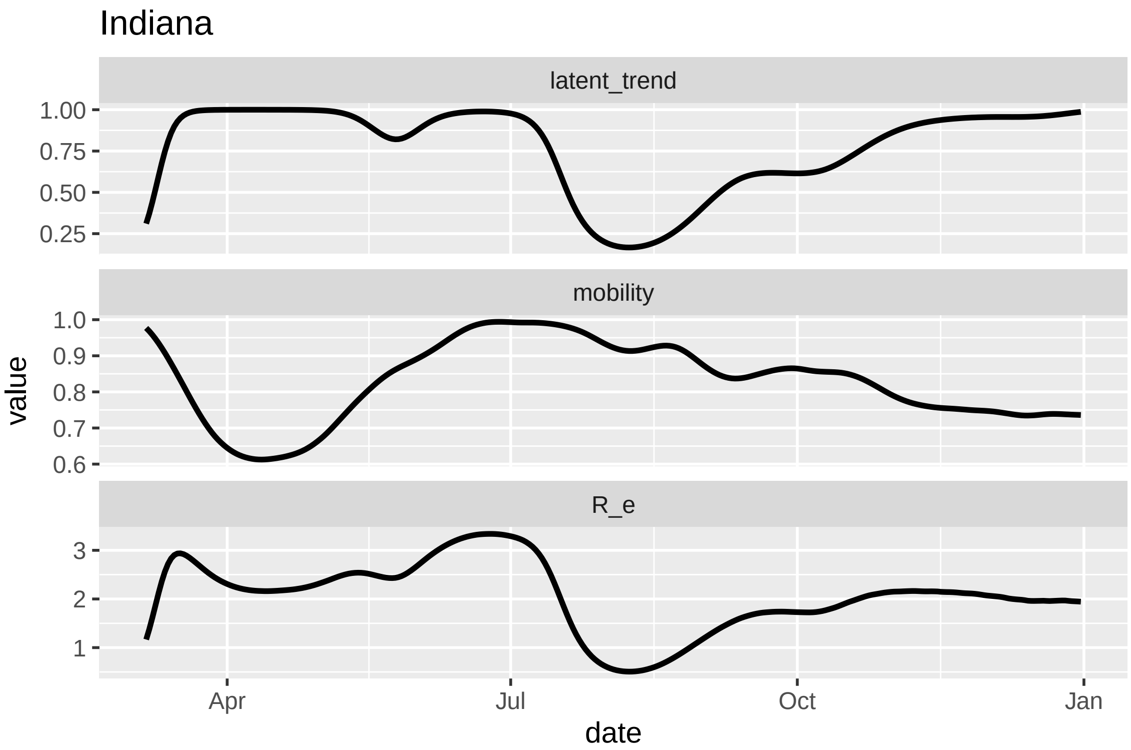
Indiana
latent_trend
1.00
0.75
0.50
0.25
mobility
1.0
0.9
0.8
0.7
0.6
value
R_e
3
2
1
Apr
Jul
Oct
Jan
date

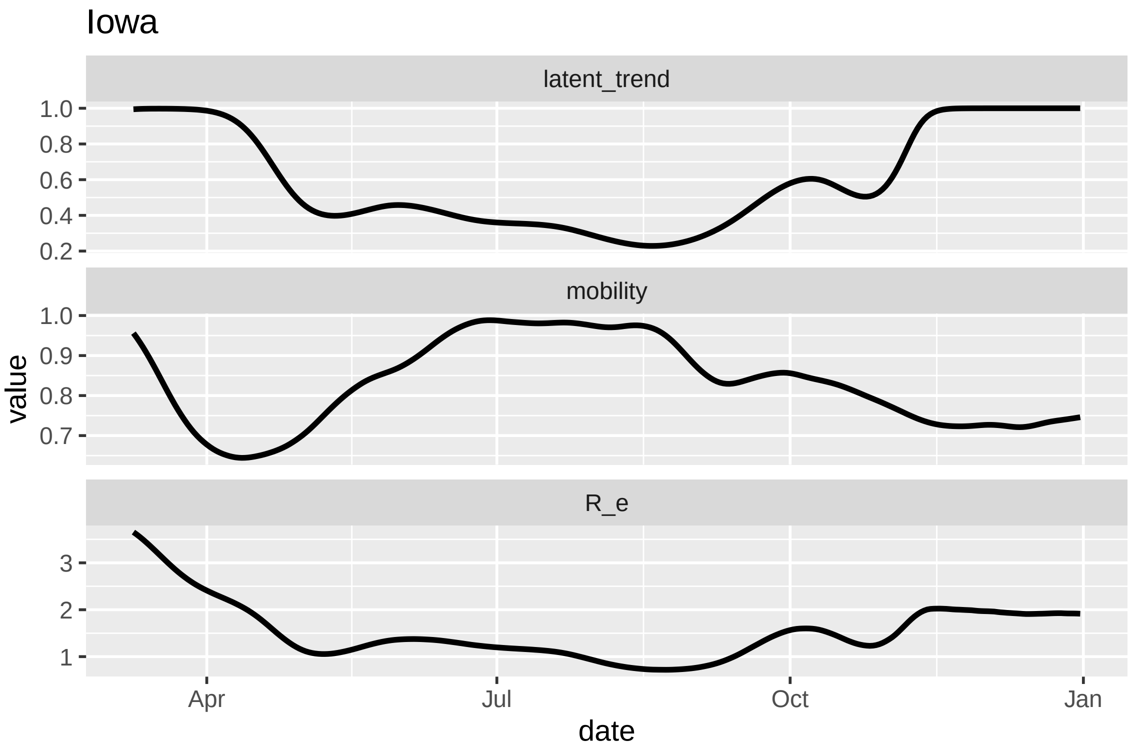
Iowa
latent_trend
1.0
0.8
0.6
0.4
0.2
mobility
1.0
0.9
0.8
0.7
value
R_e
3
2
1
Apr
Jul
Oct
Jan
date

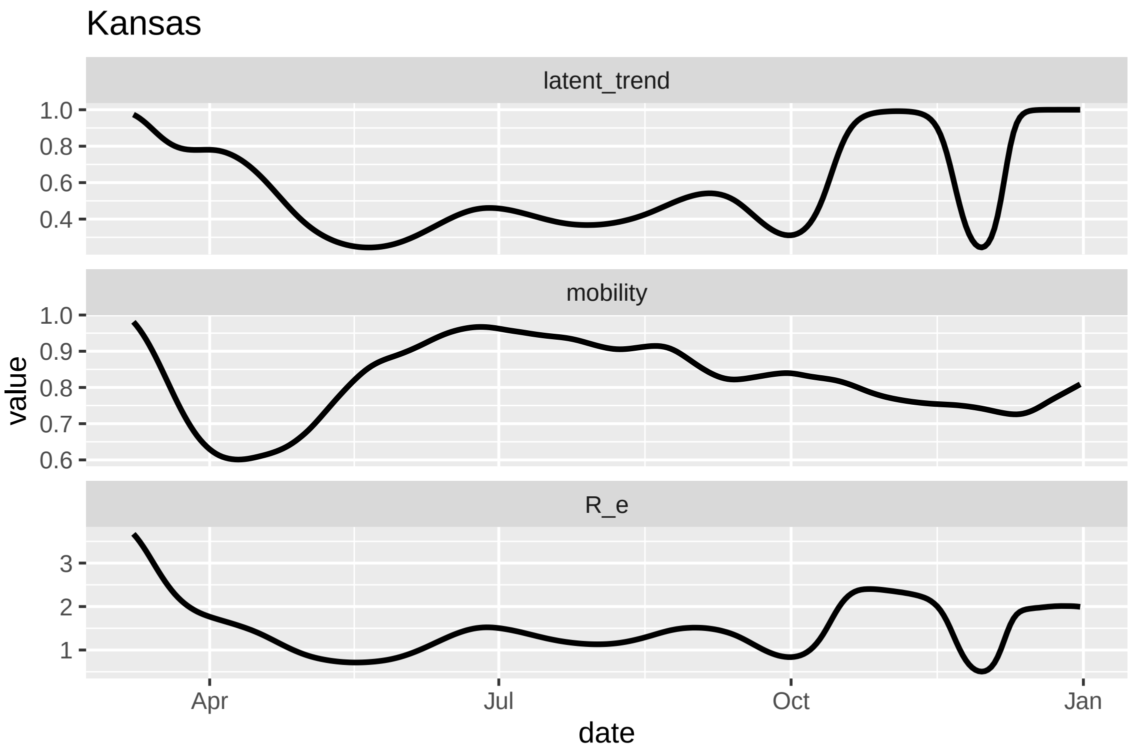
Kansas
latent_trend
1.0
0.8
0.6
0.4
mobility
1.0
0.9
0.8
0.7
0.6
value
R_e
3
2
1
Apr
Jul
Oct
Jan
date

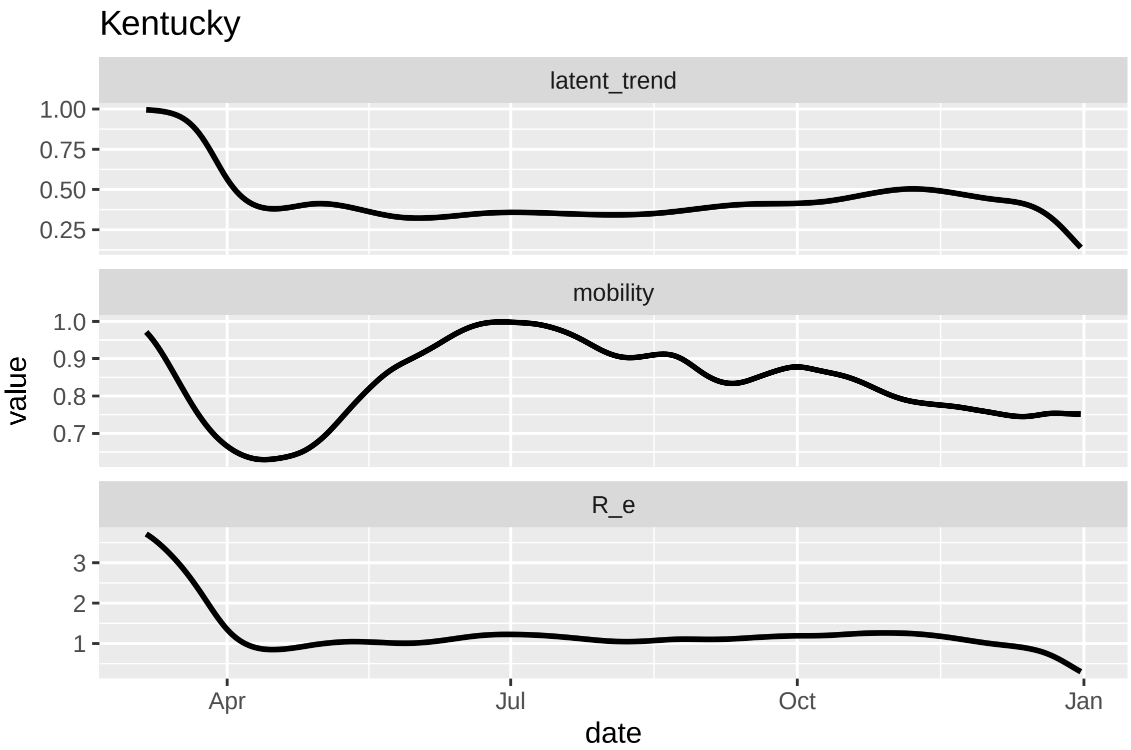
Kentucky
latent_trend
1.00
0.75
0.50
0.25
mobility
1.0
0.9
0.8
0.7
value
R_e
3
2
1
Apr
Jul
Oct
Jan
date

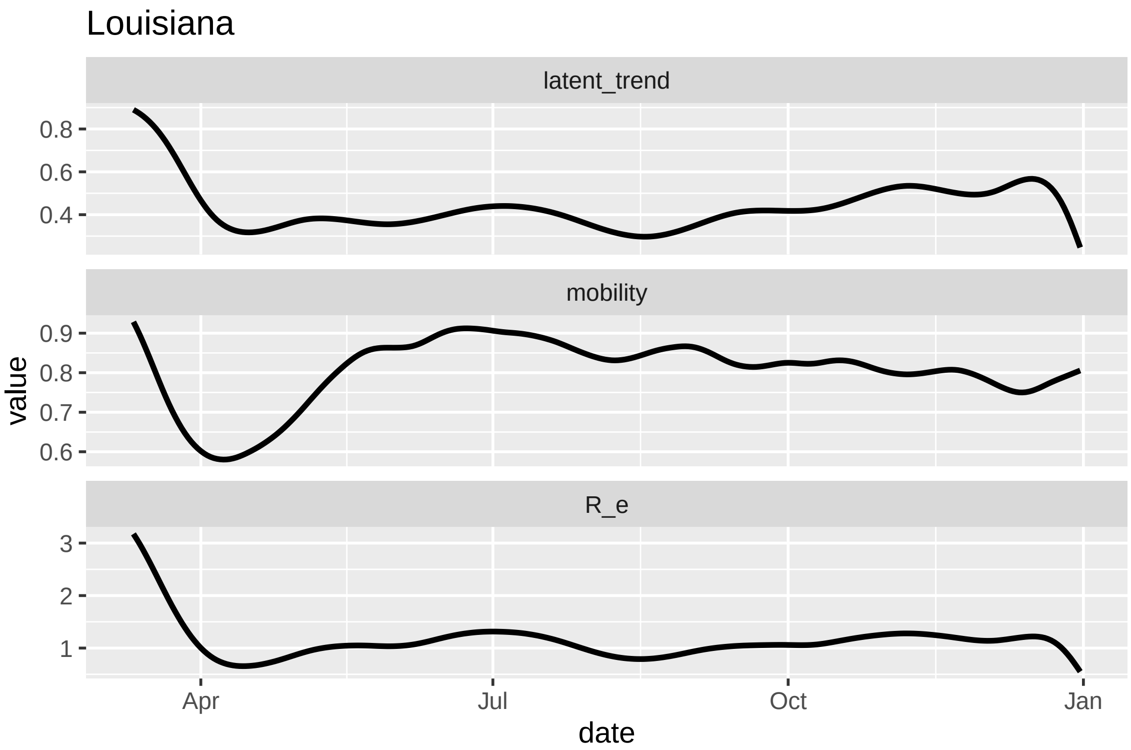

Louisiana
latent_trend
0.8
0.6
0.4
mobility
0.9
0.8
0.7
0.6
value
R_e
3
2
1
Apr
Jul
Oct
Jan
date

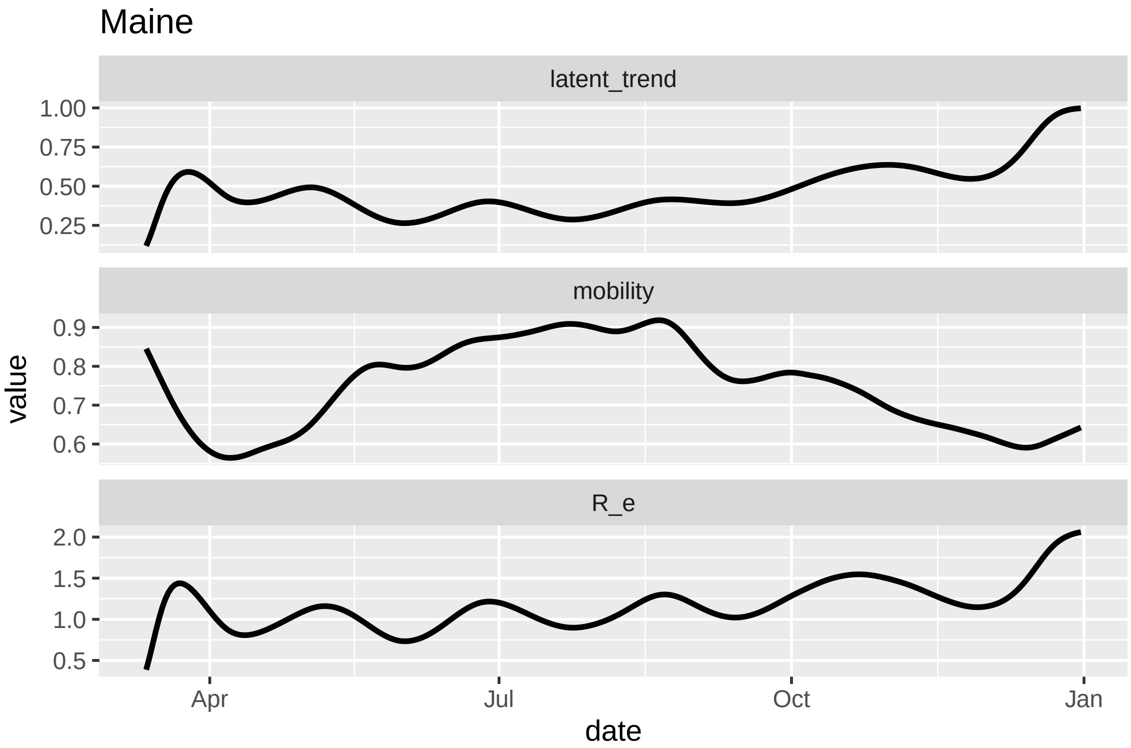

Maine
latent_trend
1.00
0.75
0.50
0.25
mobility
0.9
0.8
0.7
0.6
value
R_e
2.0
1.5
1.0
0.5
Apr
Jul
Oct
Jan
date

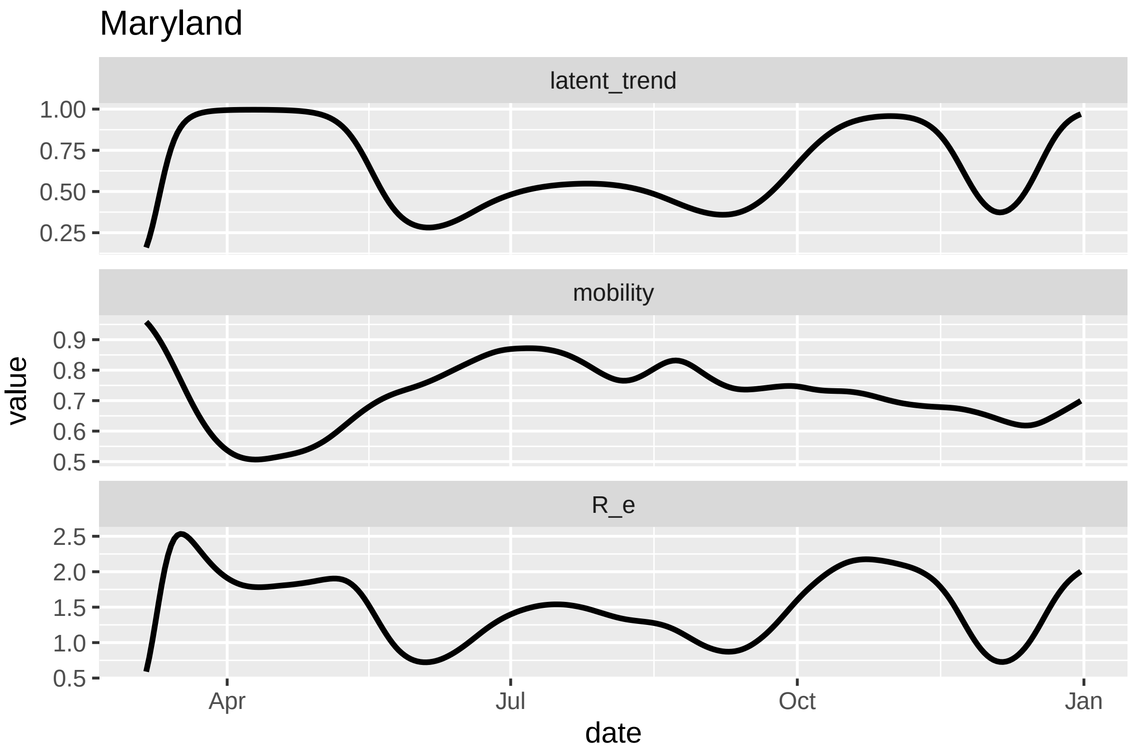
Maryland
latent_trend
mobility
R_e
value
1.00
0.75
0.50
0.25
0.9
0.8
0.7
0.6
0.5
2.5
2.0
1.5
1.0
0.5
Apr
Jul
Oct
Jan
date

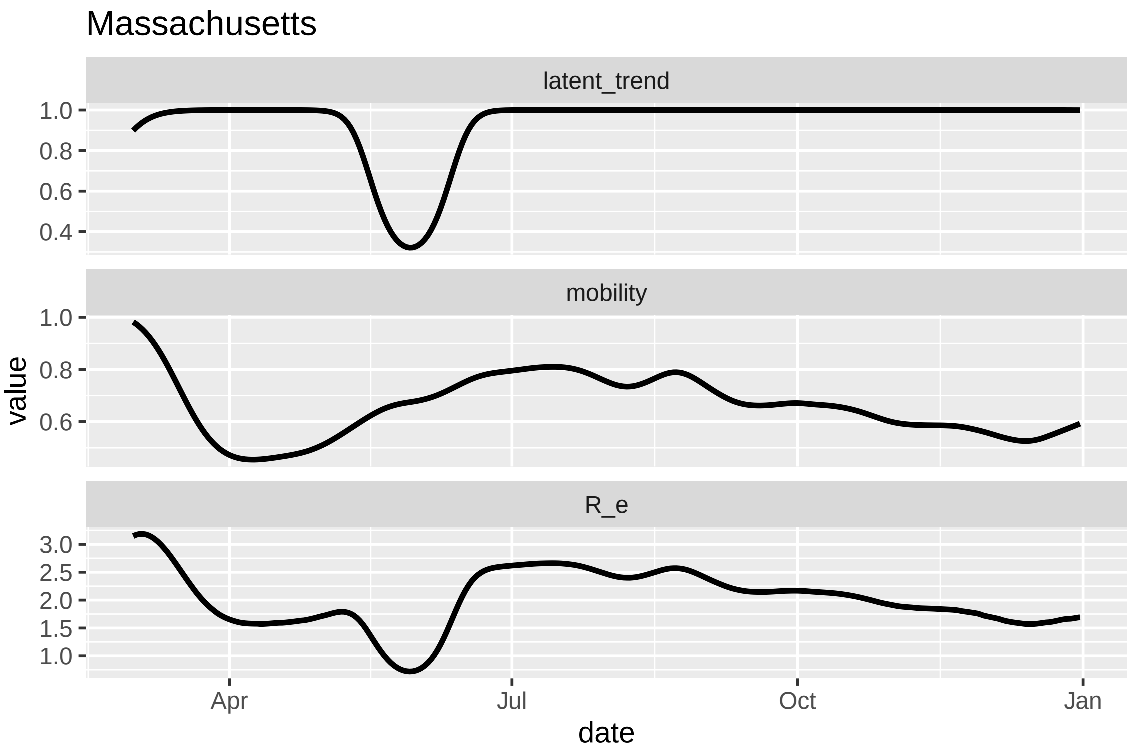
Massachusetts
latent_trend
mobility
R_e
value
1.0
0.8
0.6
0.4
1.0
0.8
0.6
3.0
2.5
2.0
1.5
1.0
Apr
Jul
Oct
Jan
date

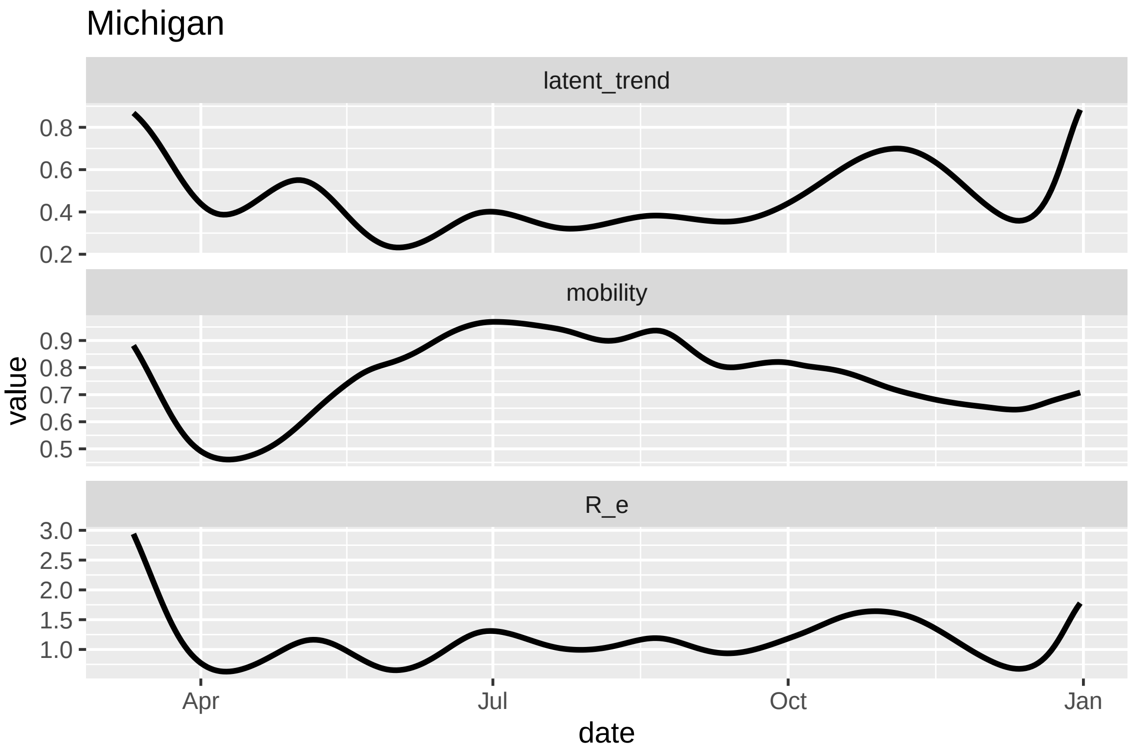
Michigan
latent_trend
mobility
R_e
value
0.2
0.4
0.6
0.8
0.5
0.6
0.7
0.8
0.9
1.0
1.5
2.0
2.5
3.0
Apr
Jul
Oct
Jan
date

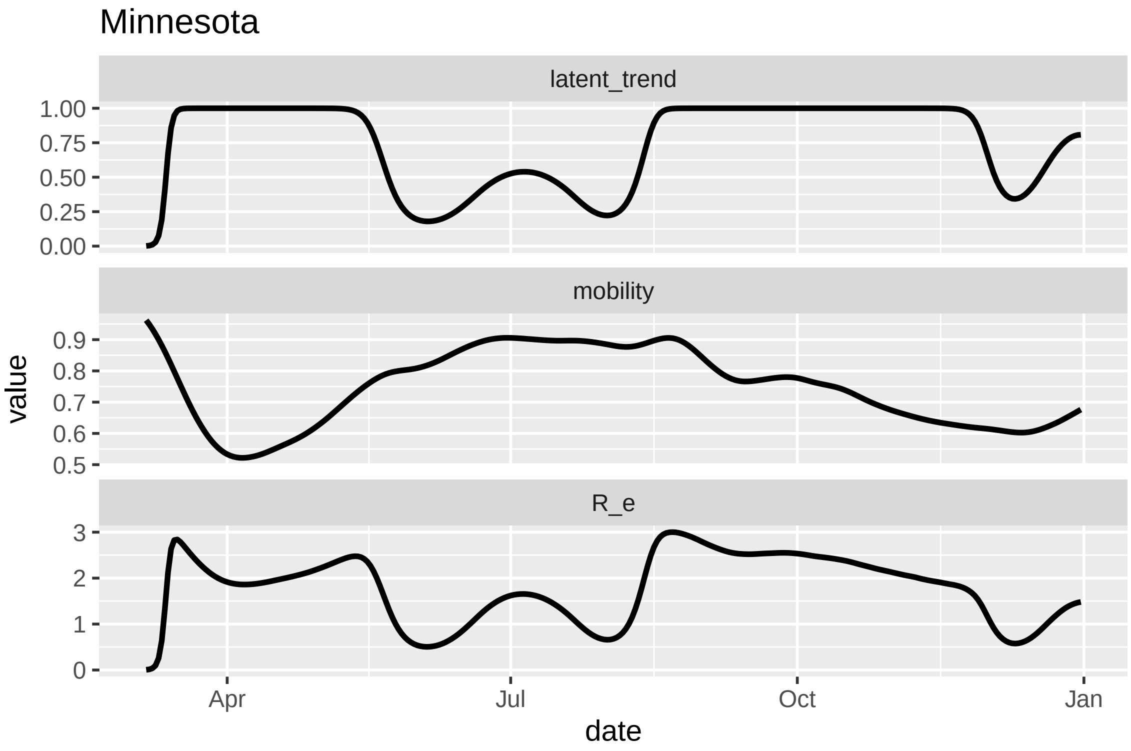
Minnesota
latent_trend
mobility
R_e
value
0.00
0.25
0.50
0.75
1.00
0.5
0.6
0.7
0.8
0.9
0
1
2
3
Apr
Jul
Oct
Jan
date

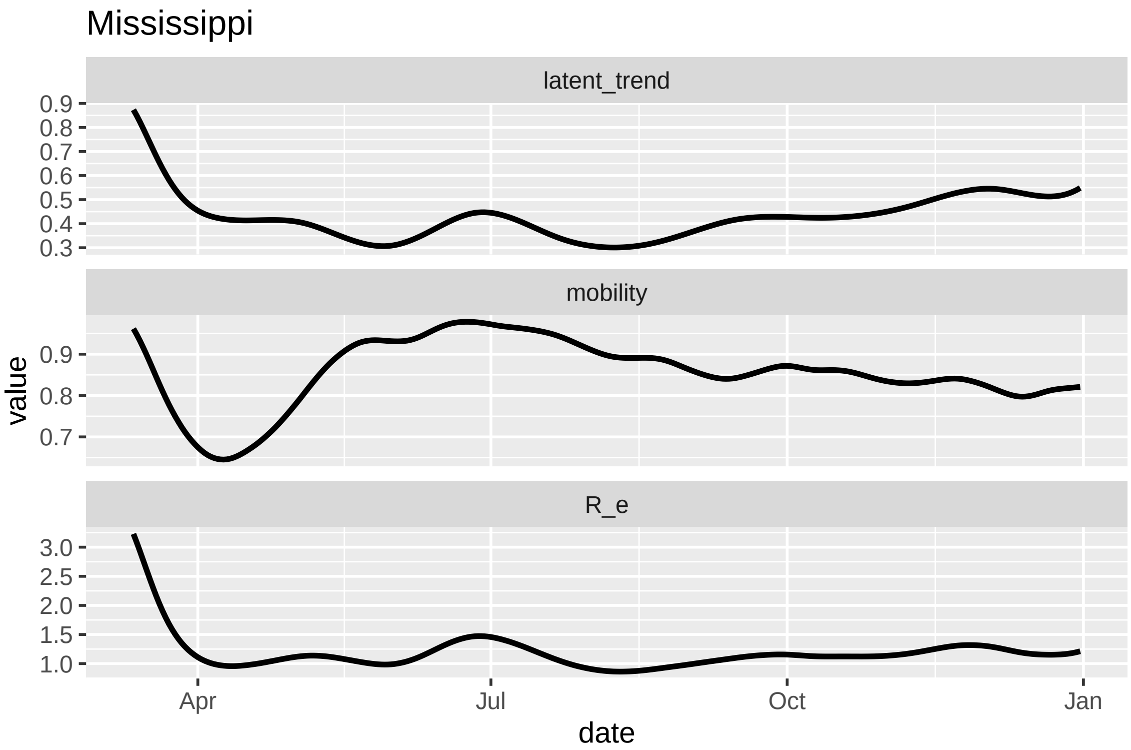
Mississippi
latent_trend
0.9
0.8
0.7
0.6
0.5
0.4
0.3
mobility
value
0.9
0.8
0.7
R_e
3.0
2.5
2.0
1.5
1.0
Apr
Jul
Oct
Jan
date

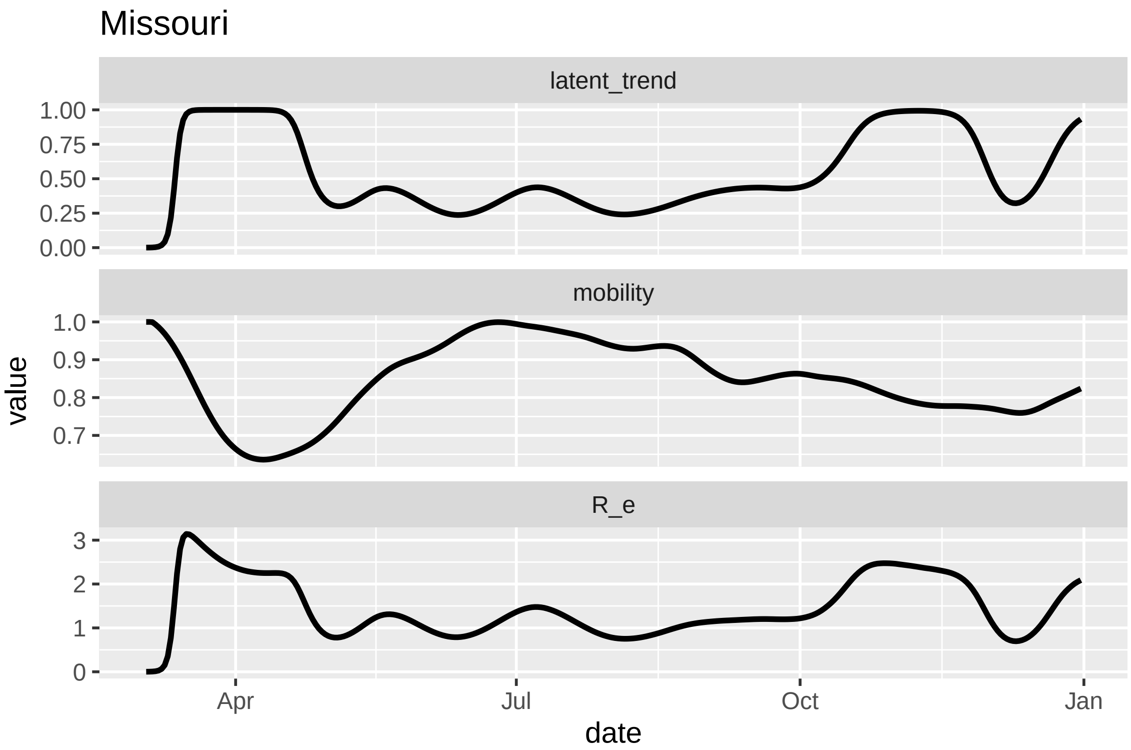
Missouri
latent_trend
1.00
0.75
0.50
0.25
0.00
mobility
value
1.0
0.9
0.8
0.7
R_e
3
2
1
0
Apr
Jul
Oct
Jan
date

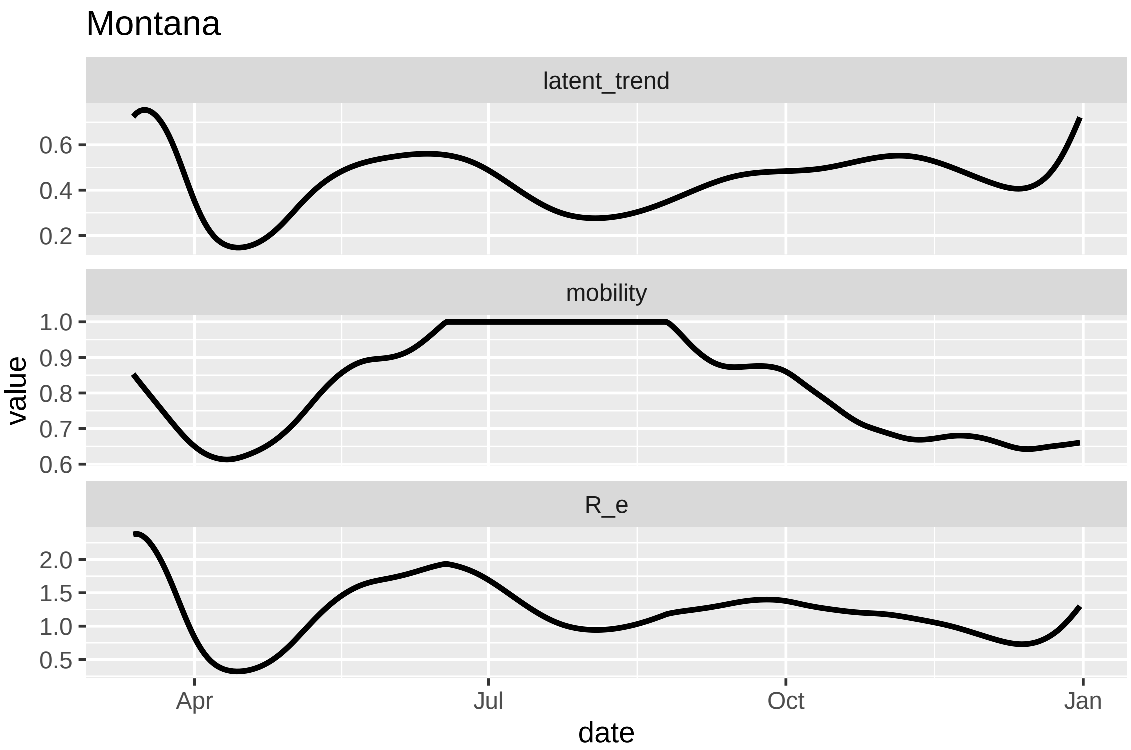
Montana
latent_trend
0.6
0.4
0.2
mobility
1.0
0.9
0.8
0.7
0.6
value
R_e
2.0
1.5
1.0
0.5
Apr
Jul
Oct
Jan
date

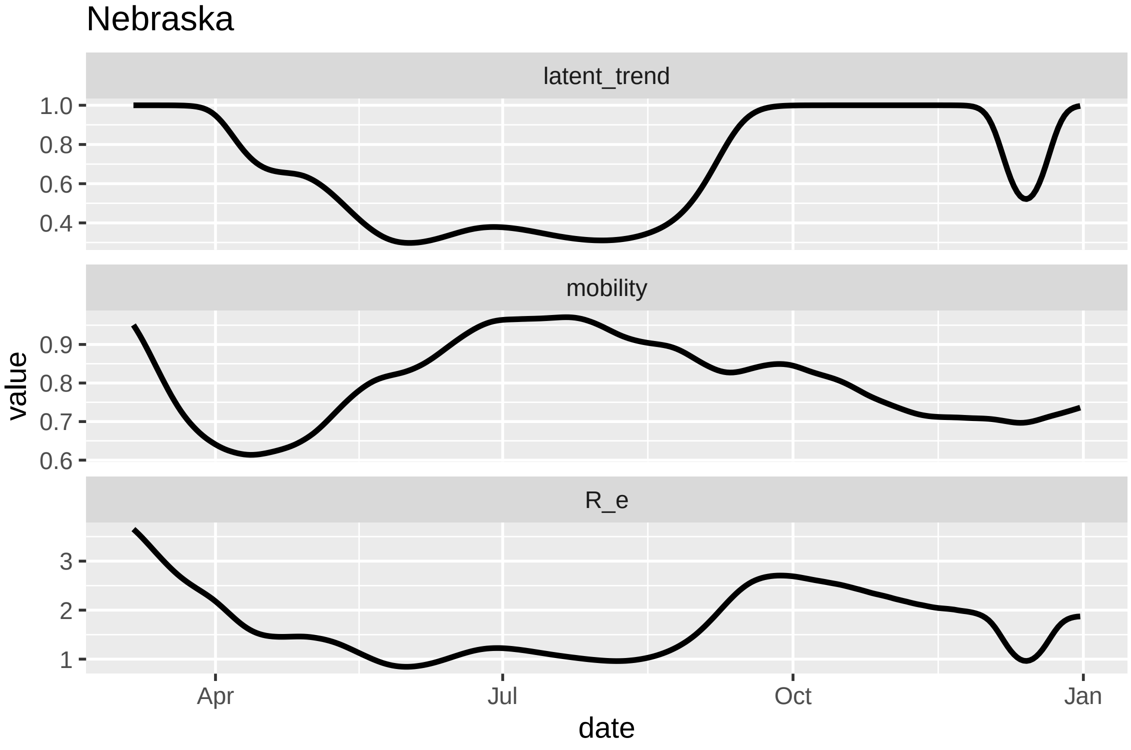
Nebraska
latent_trend
1.0
0.8
0.6
0.4
mobility
0.9
0.8
0.7
0.6
value
R_e
3
2
1
Apr
Jul
Oct
Jan
date

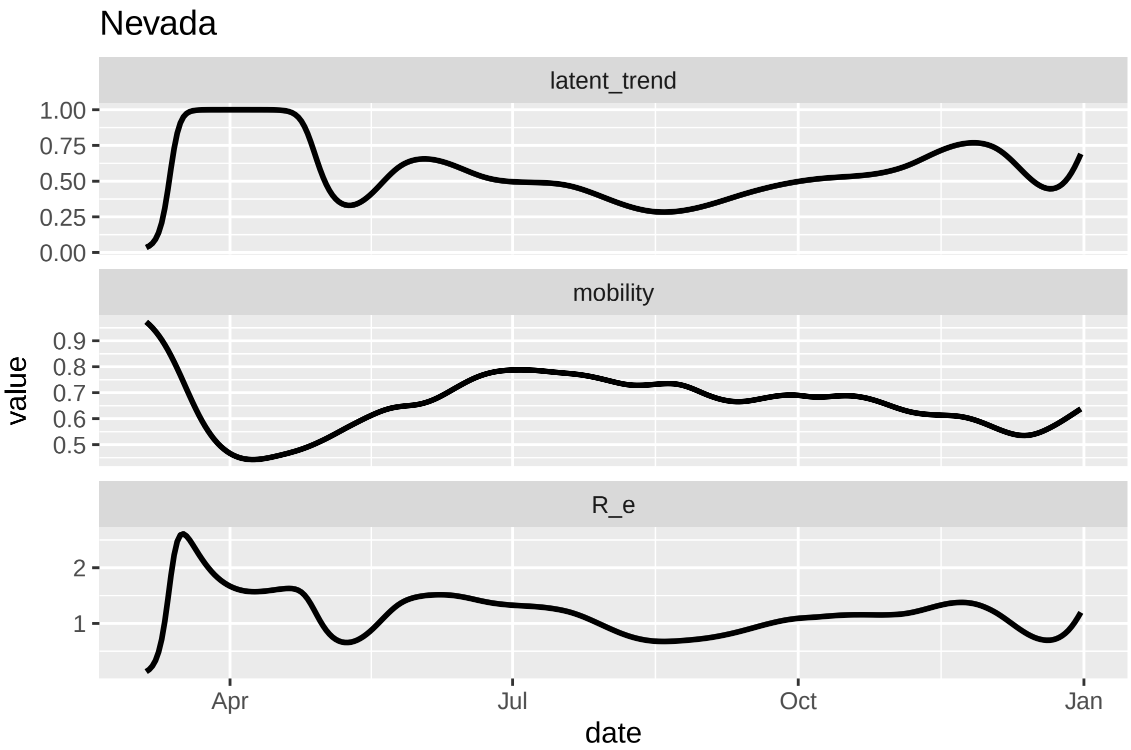
Nevada
latent_trend
1.00
0.75
0.50
0.25
0.00
mobility
0.9
0.8
0.7
0.6
0.5
R_e
2
1
value
Apr
Jul
Oct
Jan
date

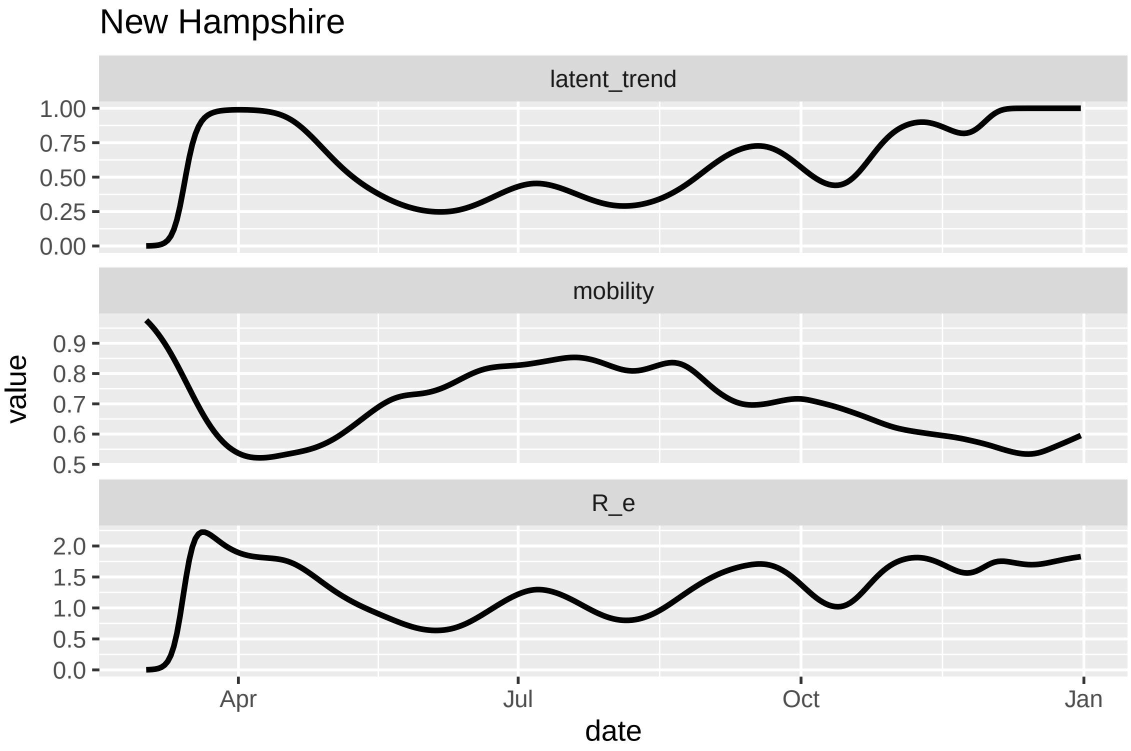
New Hampshire
latent_trend
1.00
0.75
0.50
0.25
0.00
mobility
0.9
0.8
0.7
0.6
0.5
R_e
2.0
1.5
1.0
0.5
0.0
value
Apr
Jul
Oct
Jan
date

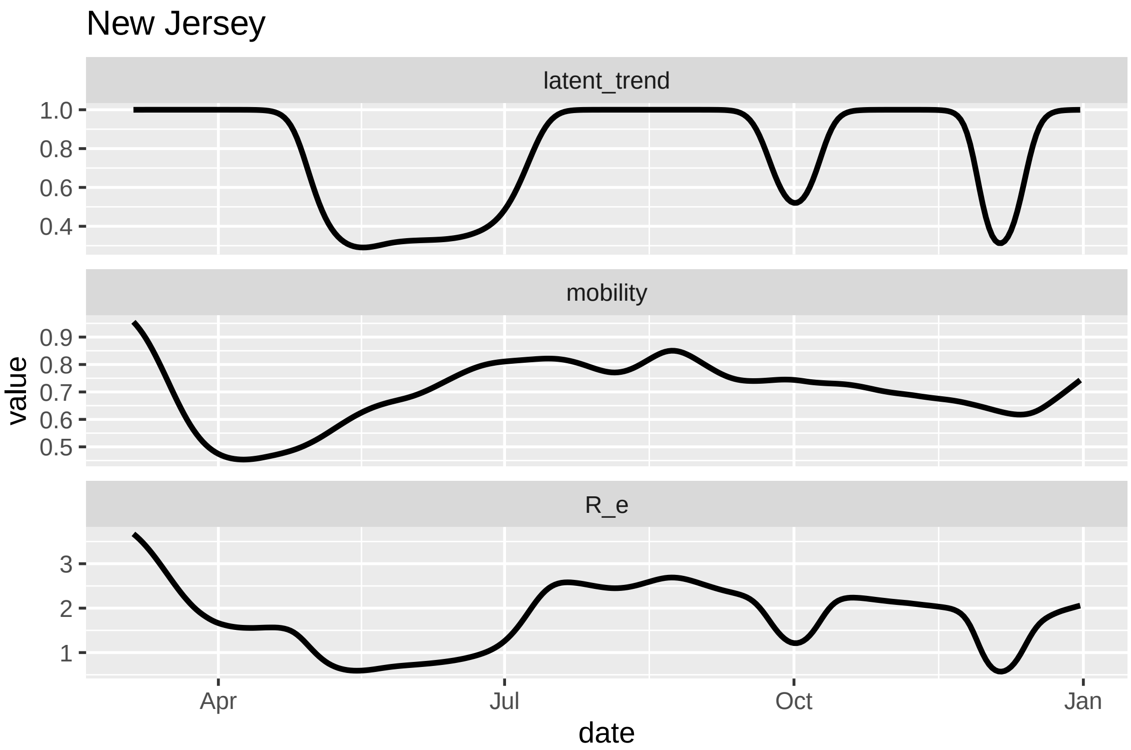
New Jersey
latent_trend
1.0
0.8
0.6
0.4
mobility
0.9
0.8
0.7
0.6
0.5
value
R_e
3
2
1
Apr
Jul
Oct
Jan
date

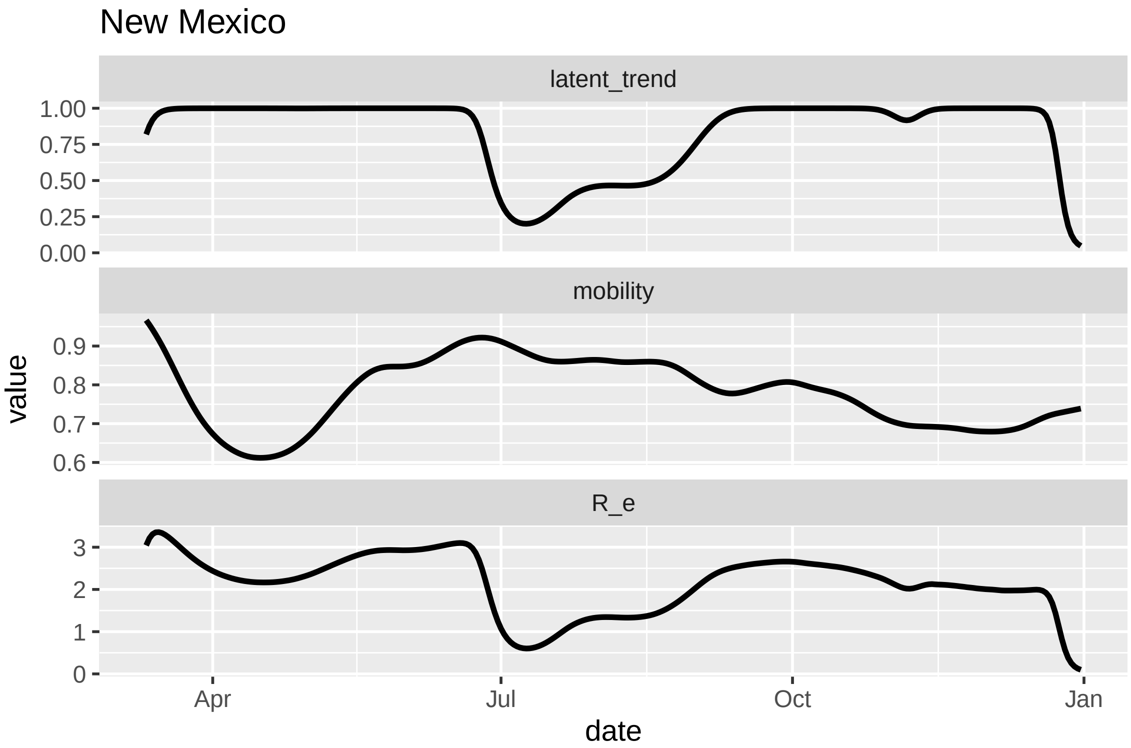
New Mexico
latent_trend
1.00
0.75
0.50
0.25
0.00
mobility
0.9
0.8
0.7
0.6
value
R_e
3
2
1
0
Apr
Jul
Oct
Jan
date

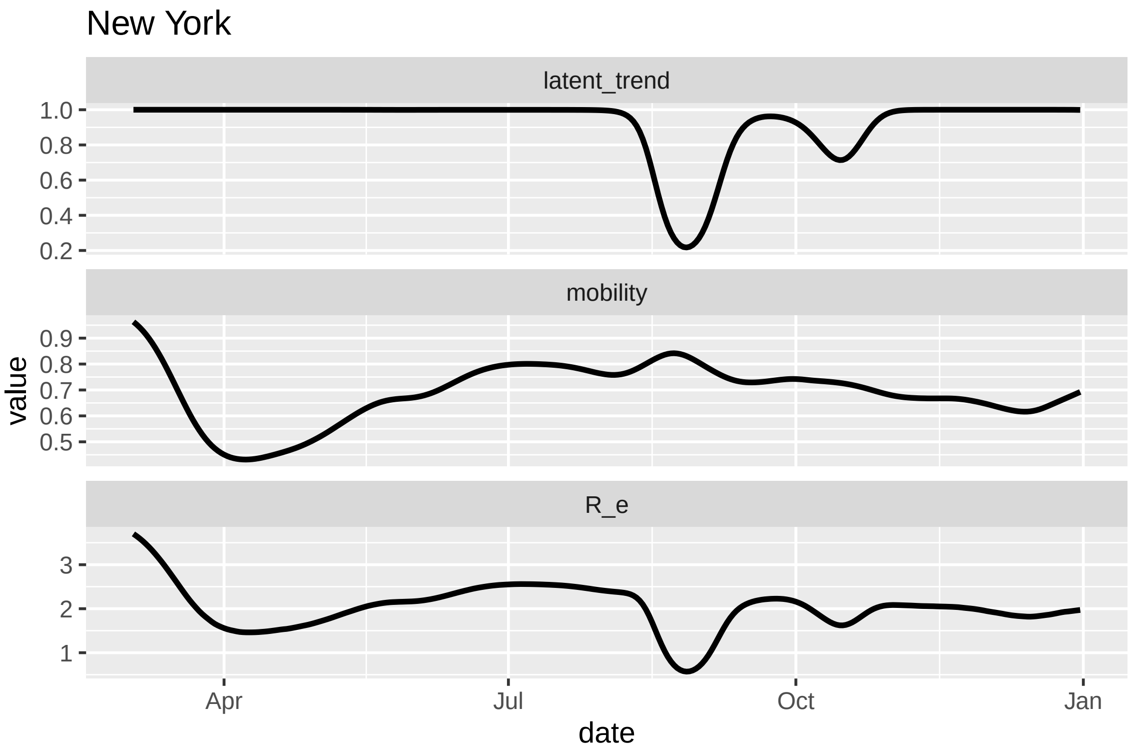
New York
latent_trend
1.0
0.8
0.6
0.4
0.2
mobility
0.9
0.8
0.7
0.6
0.5
value
R_e
3
2
1
Apr
Jul
Oct
Jan
date

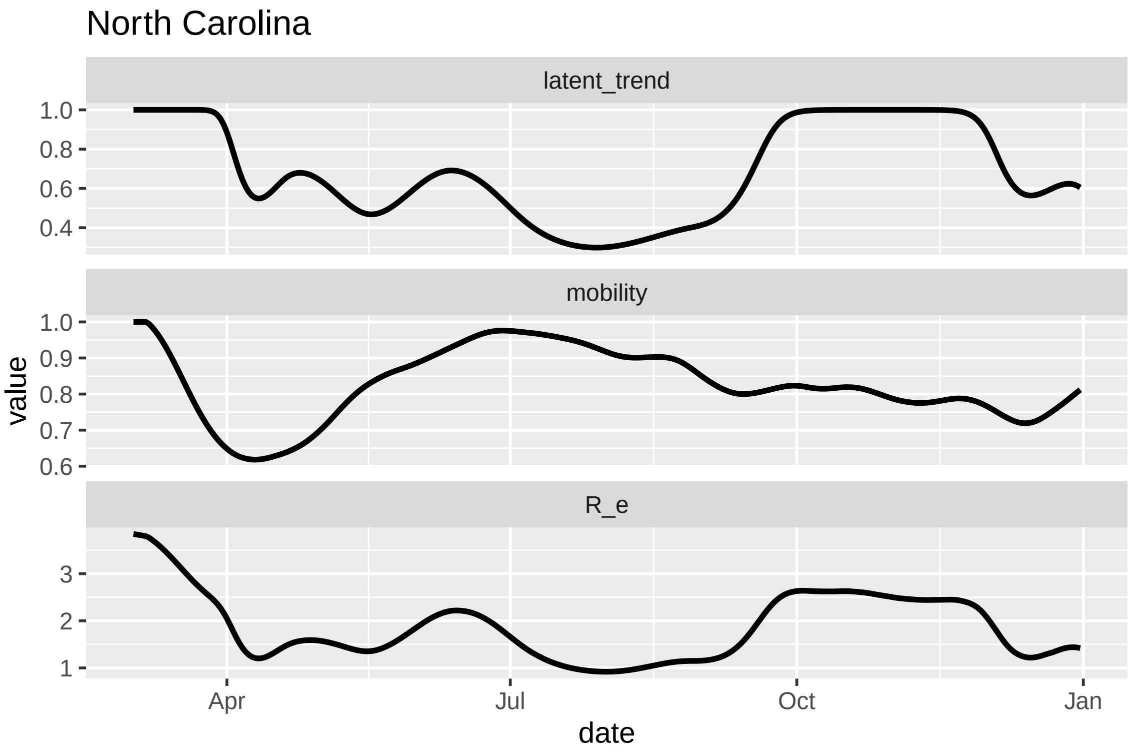
North Carolina
latent_trend
1.0
0.8
0.6
0.4
mobility
1.0
0.9
0.8
0.7
0.6
value
R_e
3
2
1
Apr
Jul
Oct
Jan
date

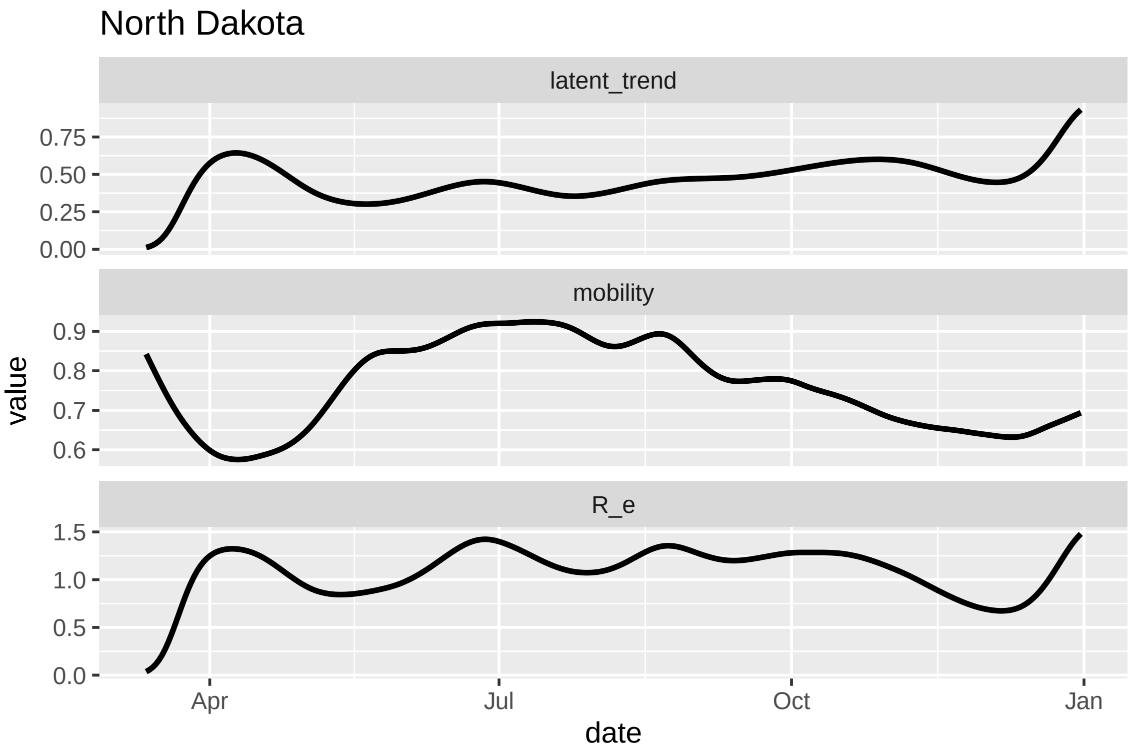
North Dakota
latent_trend
mobility
R_e
value
date
Apr
Jul
Oct
Jan

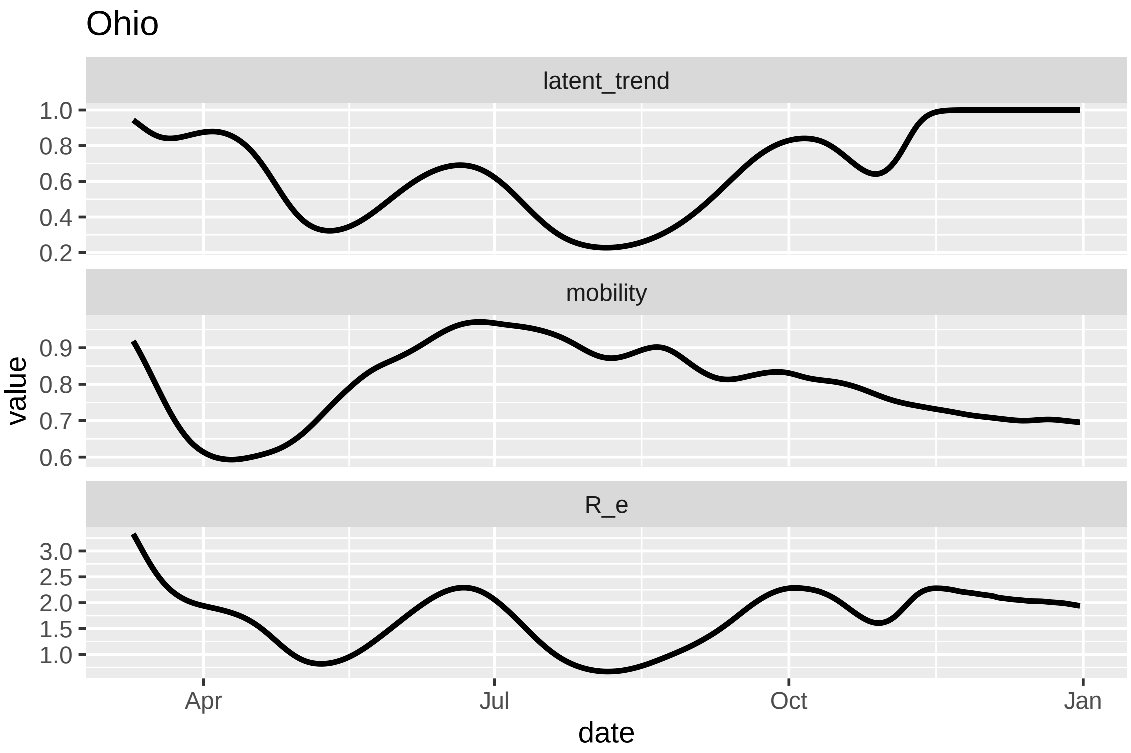
Ohio
latent_trend
mobility
R_e
value
date
Apr
Jul
Oct
Jan

Oklahoma

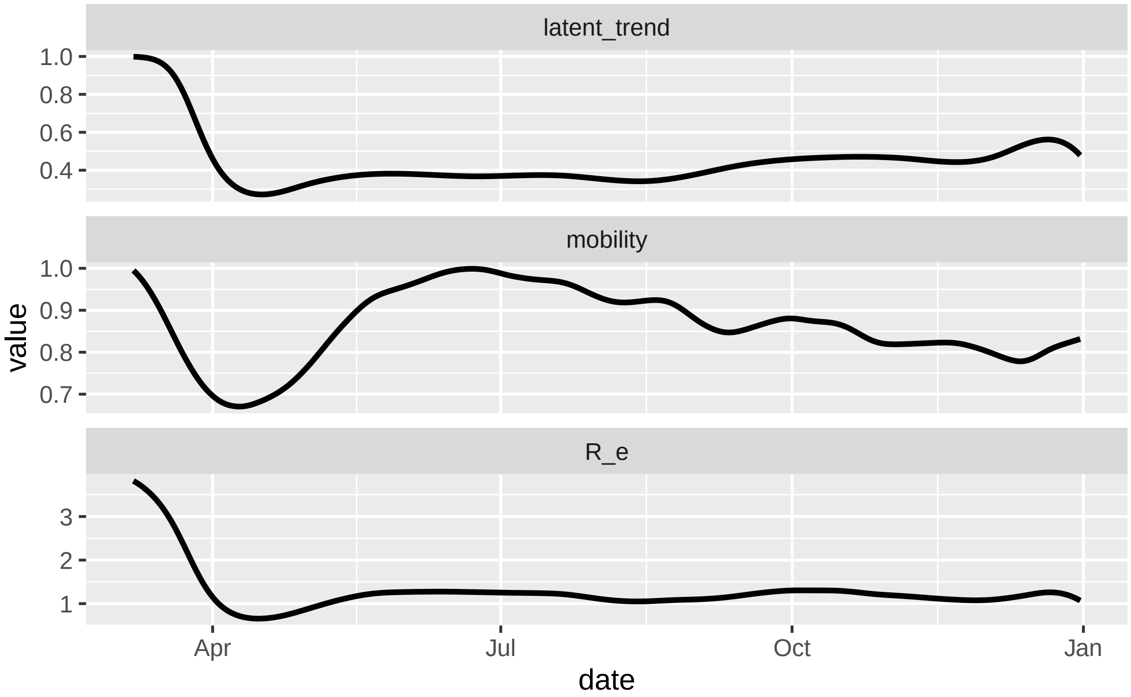

Oregon

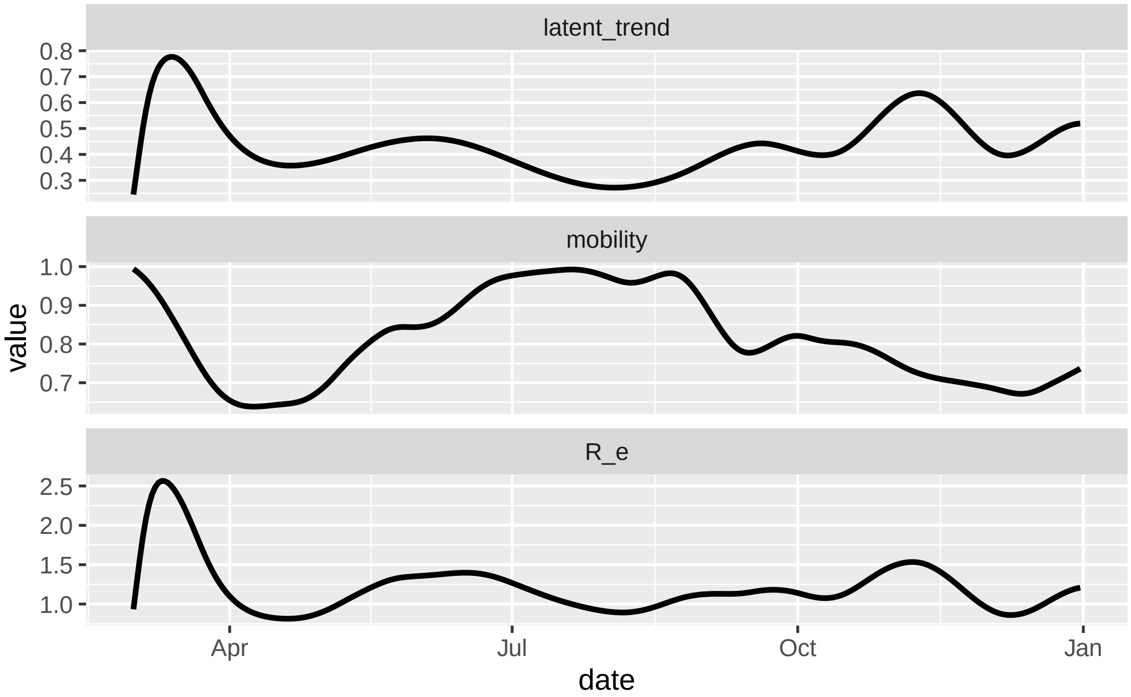

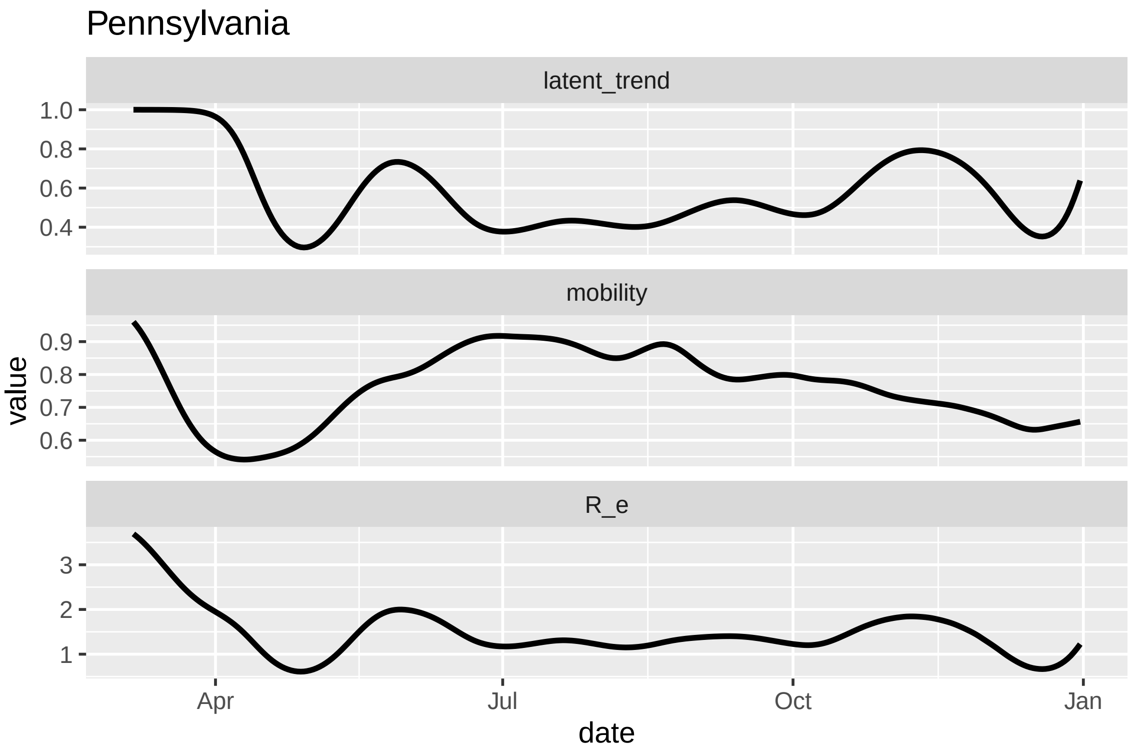
Pennsylvania
latent_trend
mobility
R_e
value
date
Apr
Jul
Oct
Jan

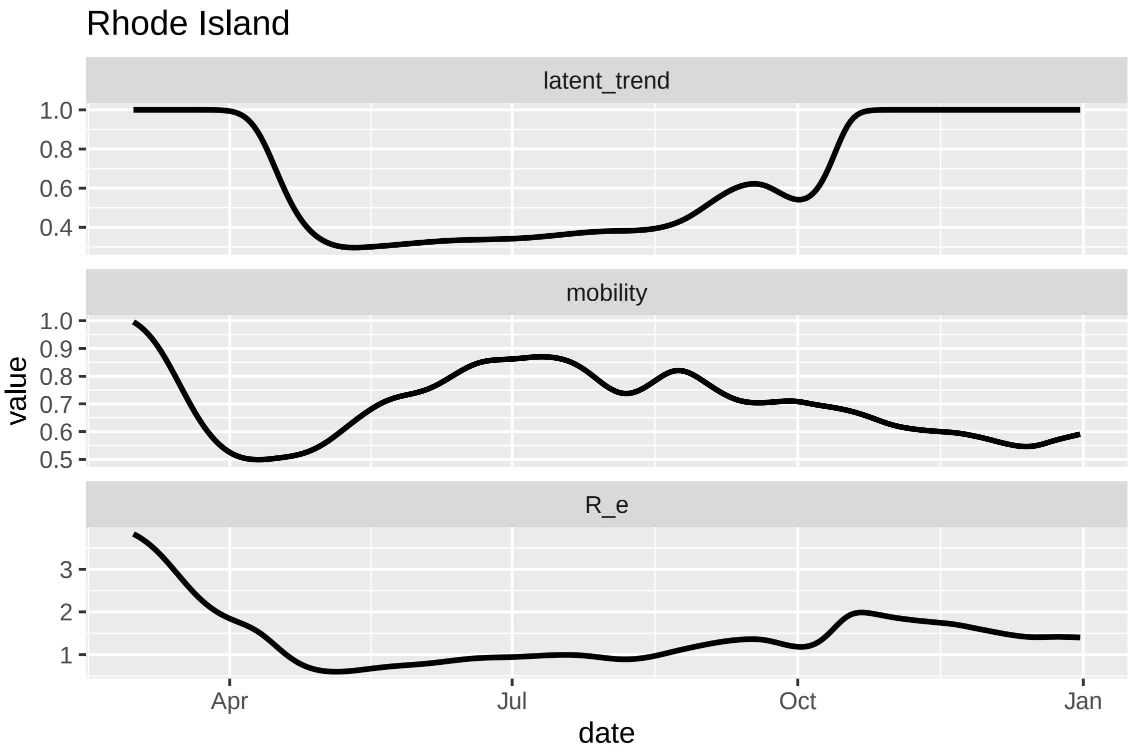
Rhode Island
latent_trend
mobility
R_e
value
date
Apr
Jul
Oct
Jan

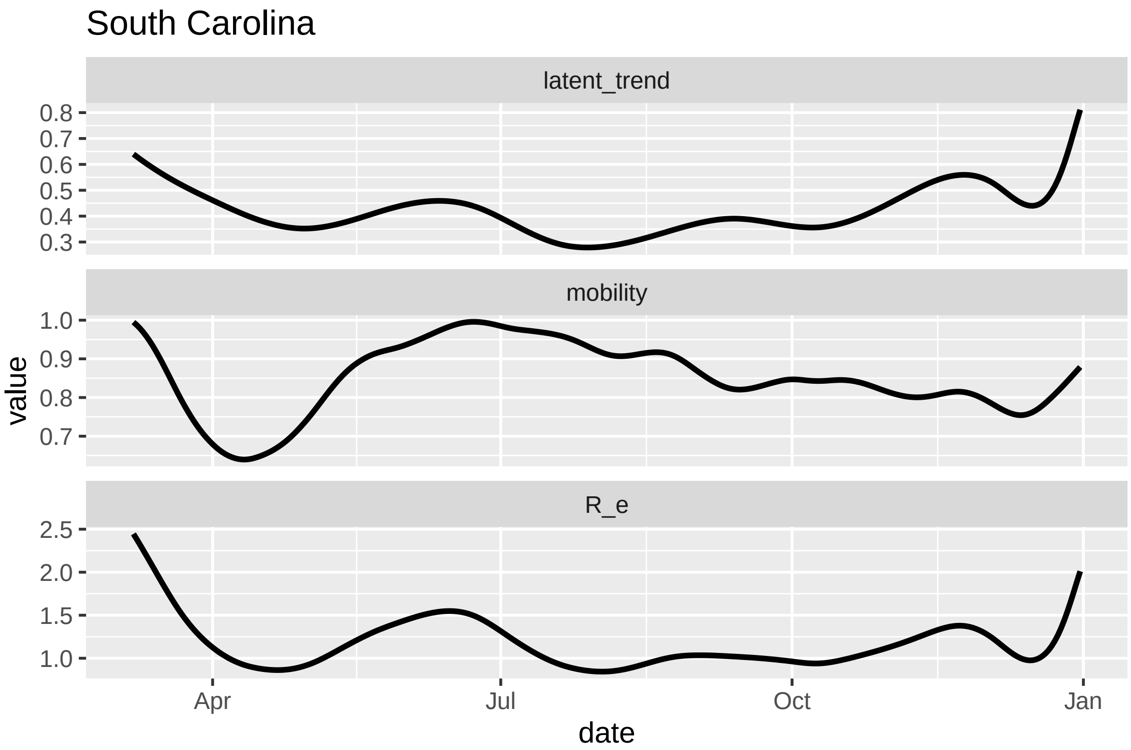
South Carolina
latent_trend
0.8
0.7
0.6
0.5
0.4
0.3
mobility
1.0
0.9
0.8
0.7
R_e
2.5
2.0
1.5
1.0
value
Apr
Jul
Oct
Jan
date

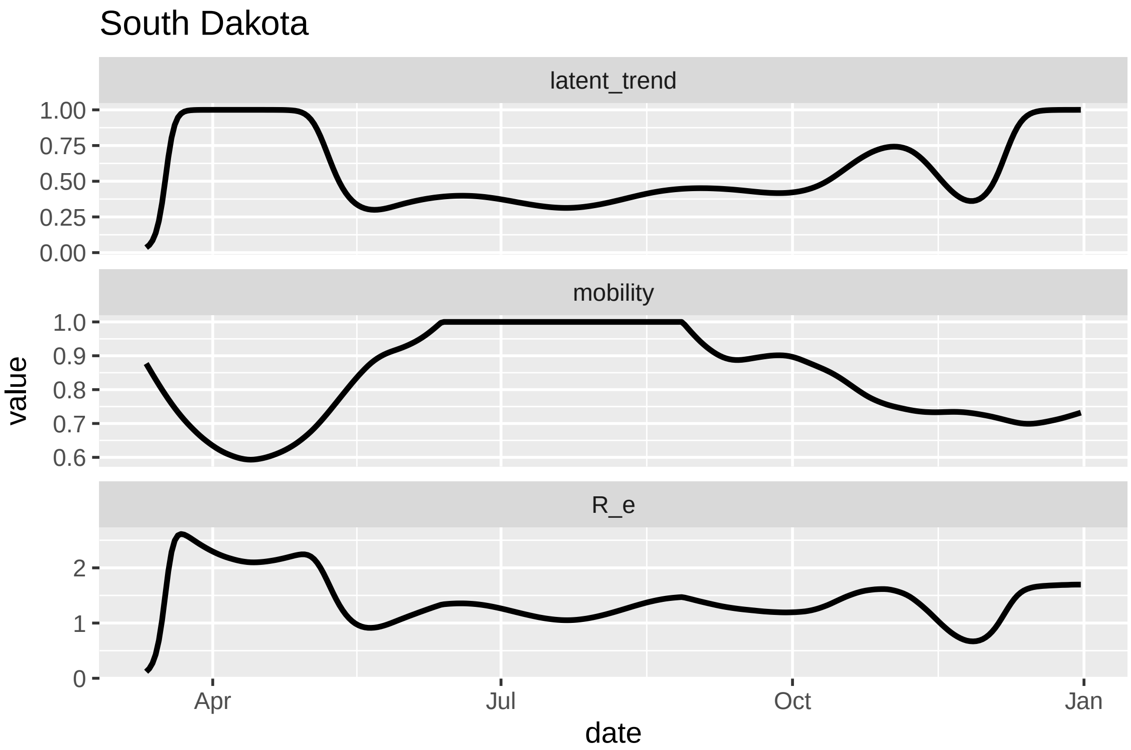
South Dakota
latent_trend
1.00
0.75
0.50
0.25
0.00
mobility
1.0
0.9
0.8
0.7
0.6
R_e
2
1
0
value
Apr
Jul
Oct
Jan
date

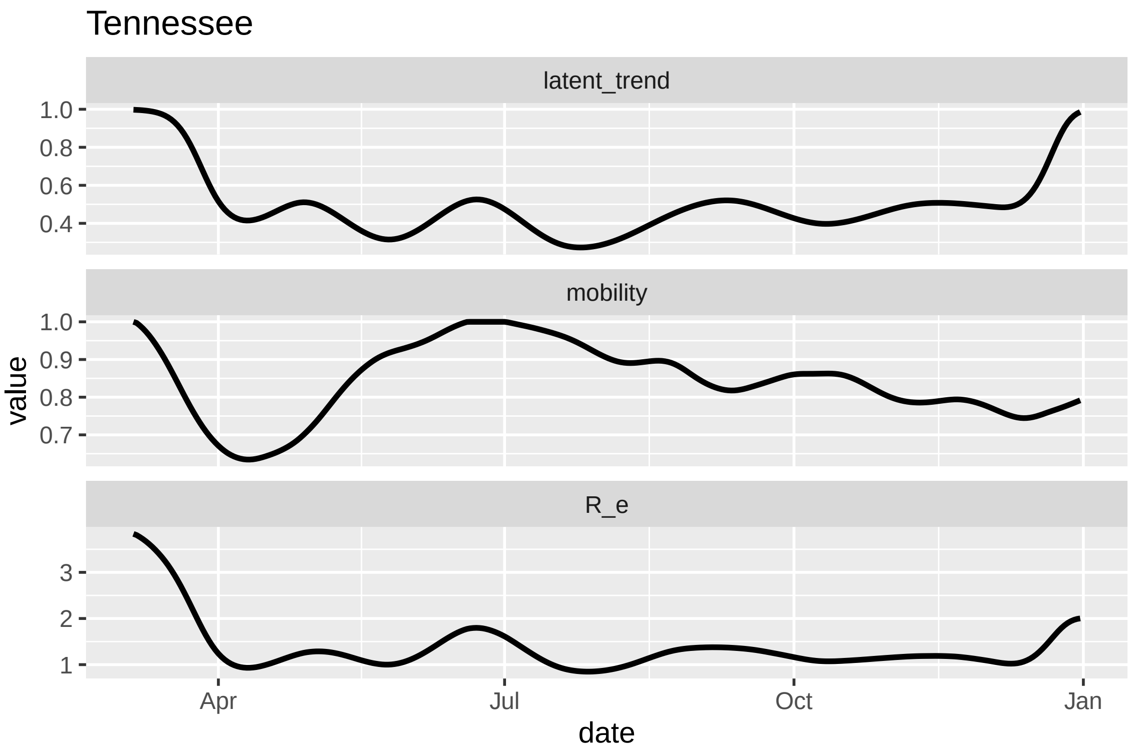

Tennessee
latent_trend
1.0
0.8
0.6
0.4
mobility
1.0
0.9
0.8
0.7
R_e
3
2
1
value
Apr
Jul
Oct
Jan
date

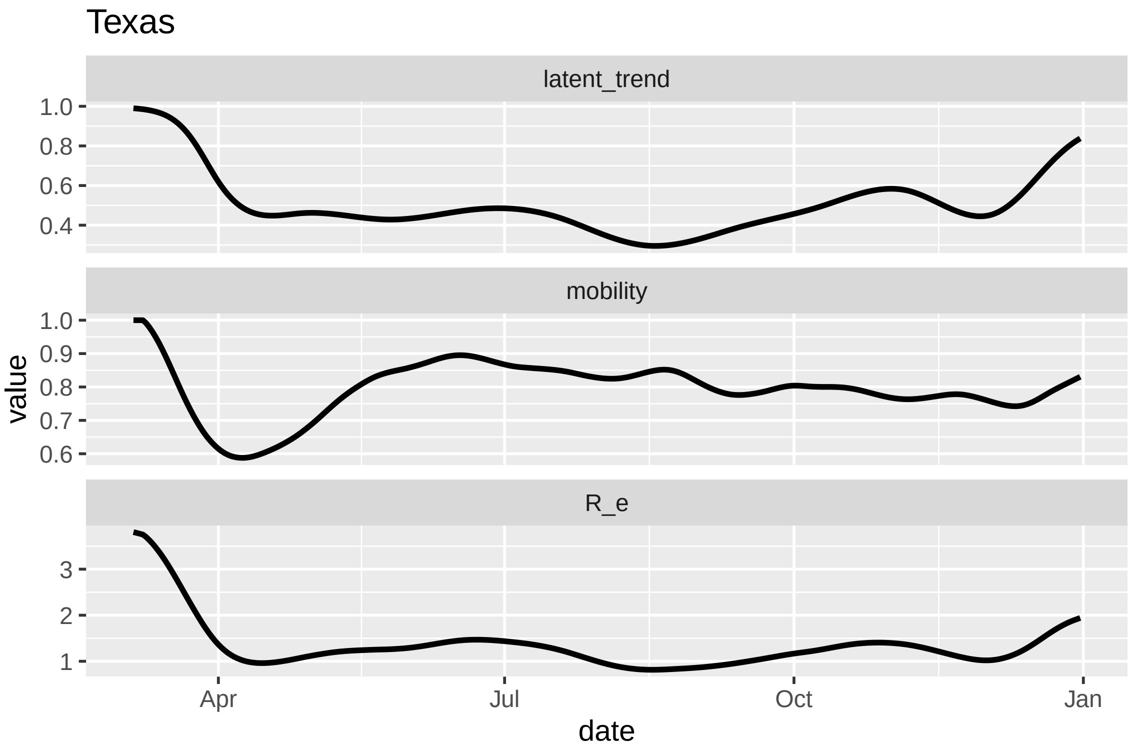

Texas
latent_trend
1.0
0.8
0.6
0.4
mobility
1.0
0.9
0.8
0.7
0.6
R_e
3
2
1
value
Apr
Jul
Oct
Jan
date

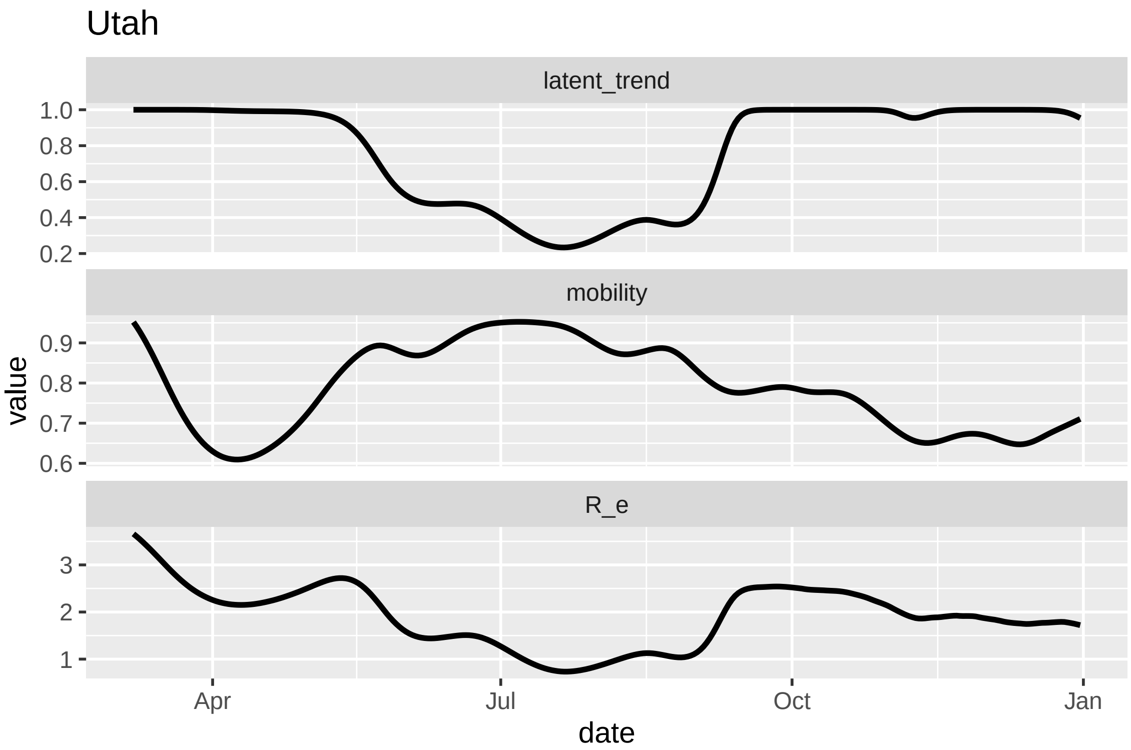
Utah
latent_trend
1.0
0.8
0.6
0.4
0.2
mobility
0.9
0.8
0.7
0.6
value
R_e
3
2
1
Apr
Jul
Oct
Jan
date

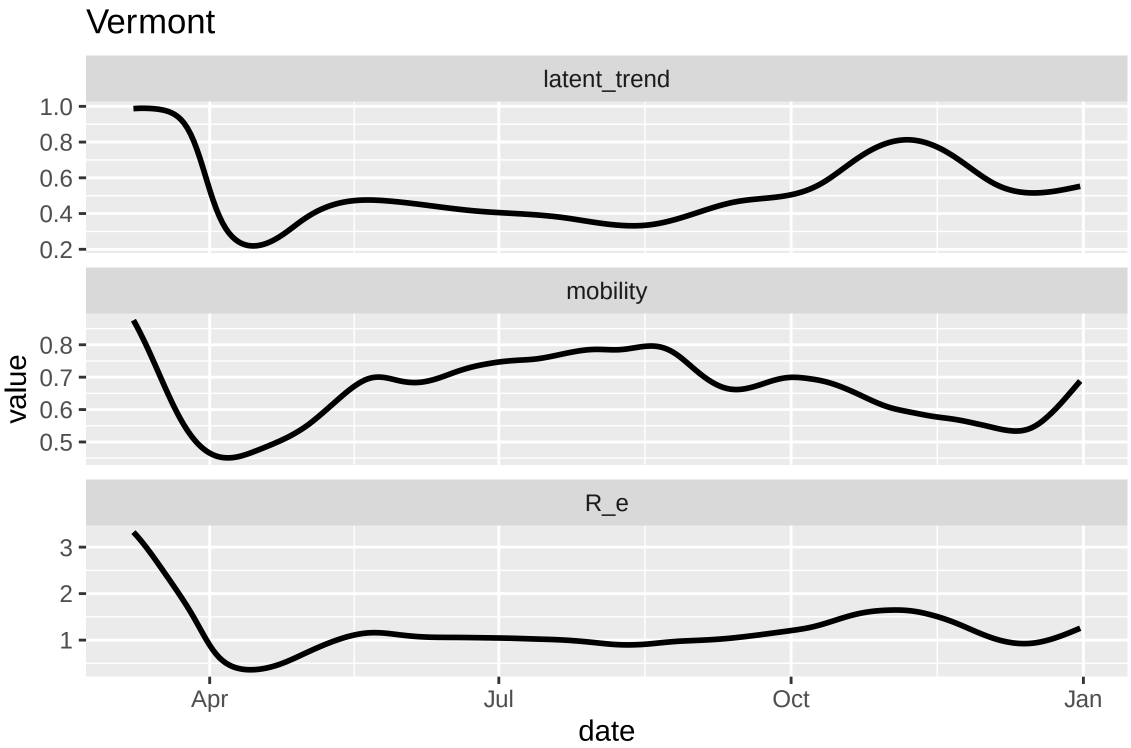
Vermont
latent_trend
1.0
0.8
0.6
0.4
0.2
mobility
0.8
0.7
0.6
0.5
value
R_e
3
2
1
Apr
Jul
Oct
Jan
date

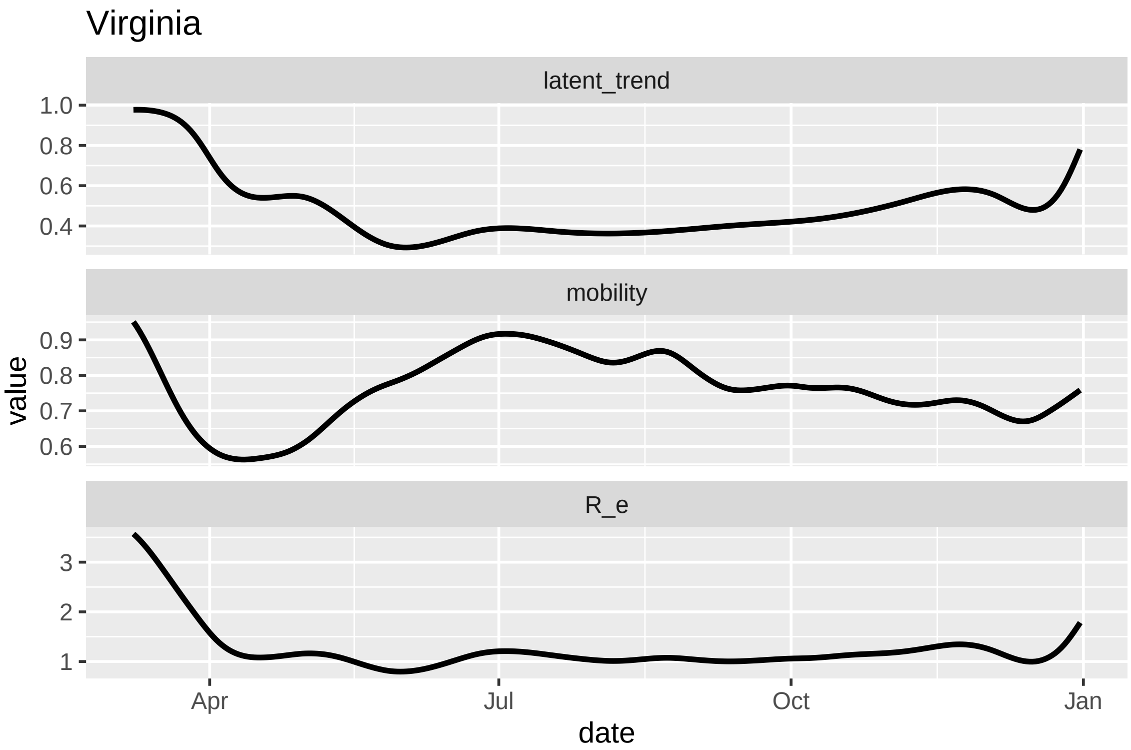
Virginia
latent_trend
1.0
0.8
0.6
0.4
mobility
0.9
0.8
0.7
0.6
value
R_e
3
2
1
Apr
Jul
Oct
Jan
date

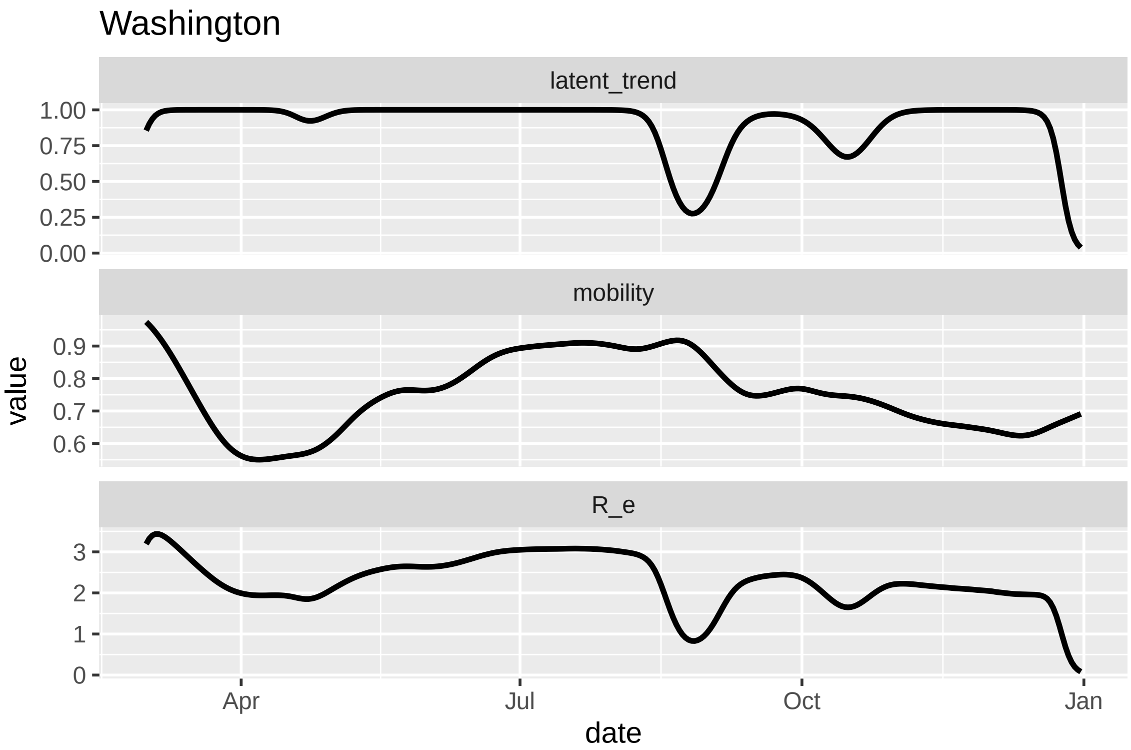
Washington
latent_trend
1.00
0.75
0.50
0.25
0.00
mobility
0.9
0.8
0.7
0.6
value
R_e
3
2
1
0
Apr
Jul
Oct
Jan
date

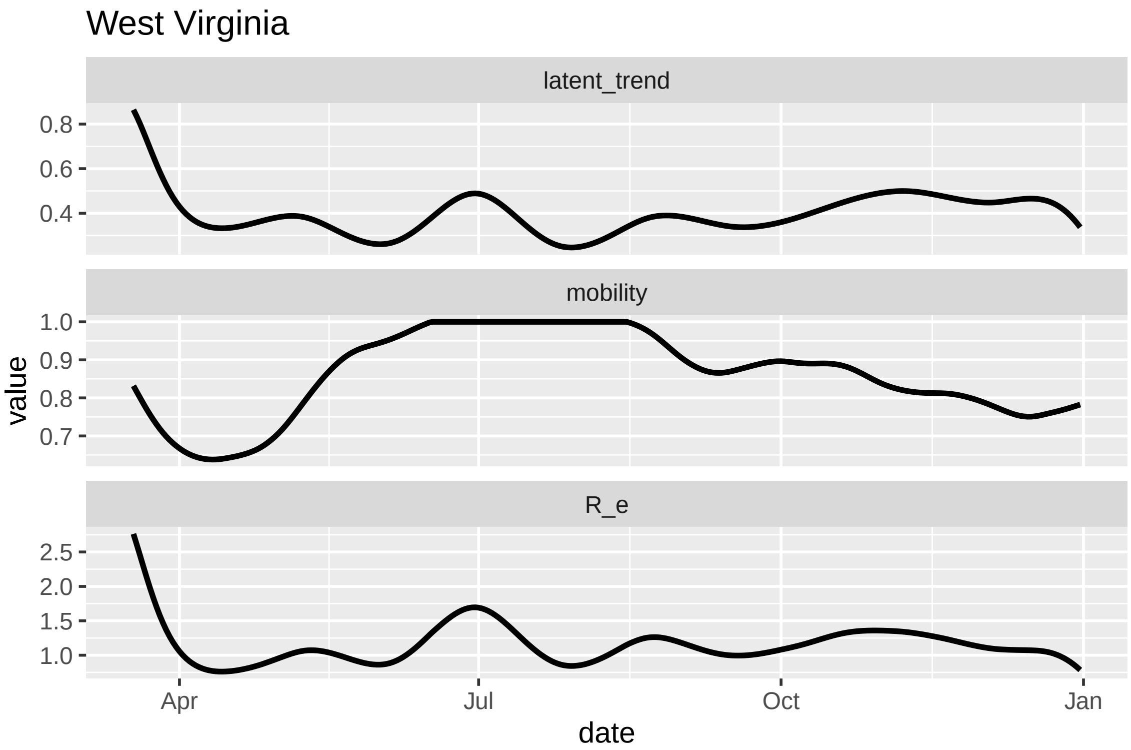
West Virginia
latent_trend
mobility
R_e
value
0.8
0.6
0.4
1.0
0.9
0.8
0.7
2.5
2.0
1.5
1.0
Apr
Jul
Oct
Jan
date

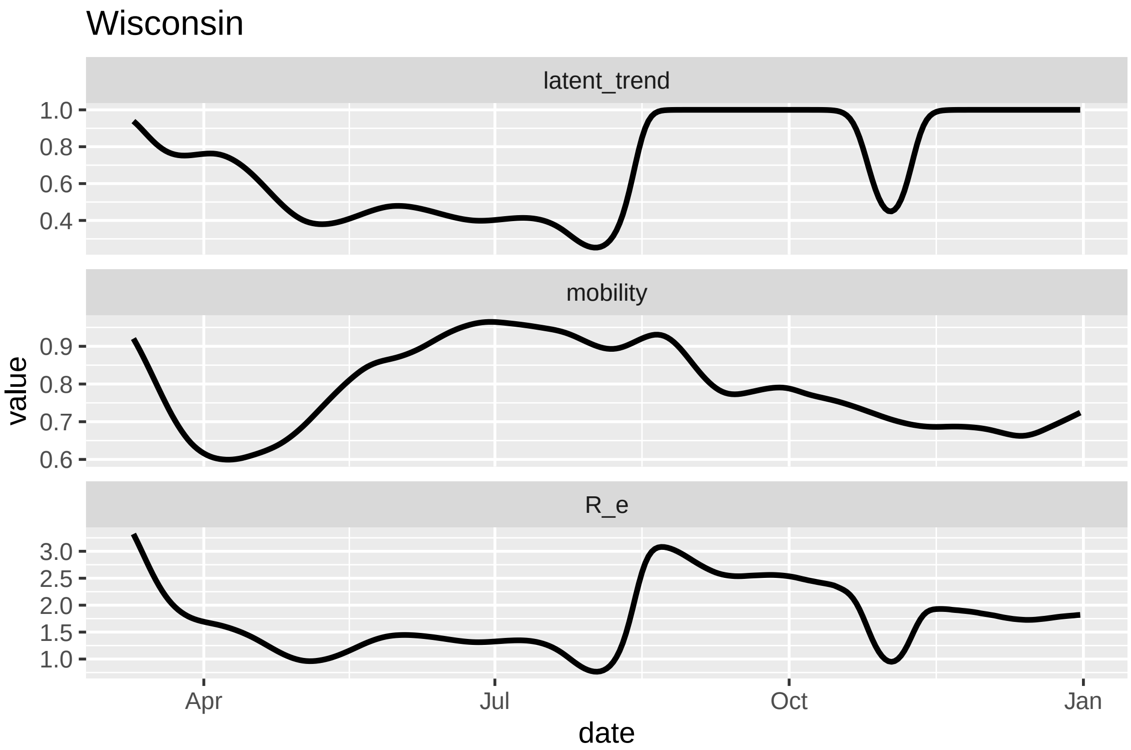
Wisconsin
latent_trend
mobility
R_e
value
1.0
0.8
0.6
0.4
0.9
0.8
0.7
0.6
3.0
2.5
2.0
1.5
1.0
Apr
Jul
Oct
Jan
date

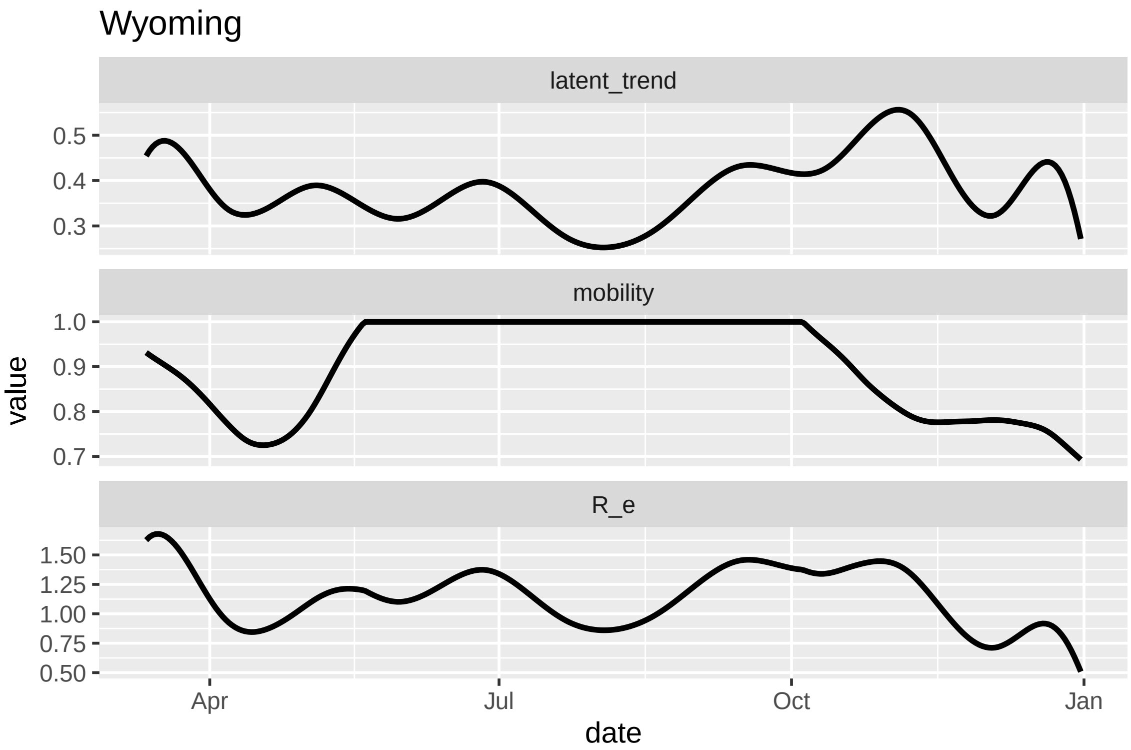
Wyoming
latent_trend
0.5
0.4
0.3
mobility
1.0
0.9
0.8
0.7
R_e
1.50
1.25
1.00
0.75
0.50
value
Apr
Jul
Oct
Jan
date
